# Efficacy, public health impact and optimal use of the Takeda dengue vaccine

**DOI:** 10.1038/s41591-025-03771-y

**Published:** 2025-06-25

**Authors:** Bethan Cracknell Daniels, Neil M. Ferguson, Ilaria Dorigatti

**Affiliations:** MRC Centre for Global Infectious Disease Analysis and the Abdul Latif Jameel Institute for Disease and Emergency Analytics, School of Public Health, Imperial College London, London, UK

**Keywords:** Viral infection, Live attenuated vaccines

## Abstract

Dengue is the most common arboviral infection, causing substantial morbidity and mortality globally. The licensing of Qdenga, a second-generation vaccine developed by Takeda Pharmaceuticals, is therefore timely, but the potential public health impact of vaccination across transmission settings needs to be evaluated. To address this, we characterized Qdenga’s efficacy profile using mathematical models calibrated to published clinical trial data and estimated the public health impact of routine vaccine use. We find that efficacy against both virologically confirmed dengue and hospitalization depends on the infecting serotype, serological status and age. We estimate that vaccination of children aged over 6 years in moderate-to-high dengue transmission settings (average seroprevalence in 9-year-olds > 60%) could reduce the burden of hospitalized dengue by 10–22% on average over 10 years. We find some evidence of a risk of vaccine-induced disease enhancement in seronegative vaccine recipients for dengue serotypes 3 and 4, especially for children under 6 years of age. Because of this, the benefits of vaccination in lower transmission settings are more uncertain, and more data on the long-term efficacy of Qdenga against serotypes 3 and 4 are needed.

## Abstract (web summary)

Using mathematical models calibrated to clinical trial results on vaccine efficacy, this study projects the potential impact of the vaccine at population level, showing the conditions for it to be effective in reducing cases and hospitalizations and helping to design vaccination campaigns.

## Main

With more than half of the world’s population currently at risk of dengue infection, novel control methods, including vaccines, are urgently needed to reduce disease burden and the resulting economic impacts. Historically, developing safe and effective dengue vaccines has been challenging due to the presence of four antigenically distinct dengue serotypes (DENV1–4) that elicit cross-reactive immunity and which can enhance the severity of secondary infections, such that 2–4% result in severe dengue^1^. Suggested mechanisms of disease enhancement include antibody-dependent enhancement, in which poor avidity or insufficient concentrations of neutralizing antibody titers facilitate the entry of DENV into host cells, increasing viral replication^2^. The first licensed dengue vaccine, Dengvaxia, developed by Sanofi Pasteur, was belatedly found to increase the hospitalization risk in dengue-naive (seronegative) vaccine recipients (hazard ratio 1.75, 95% confidence interval (CI): 1.14–2.70)^3^, confirming earlier modeling of the phase III trial of that vaccine, which had highlighted this potential risk^4^. Consequently, Dengvaxia is now indicated for use only in individuals with prior dengue exposure (seropositives) and, due to the absence of an accurate rapid antibody test for dengue to date, it is in limited use. Sanofi Pasteur recently announced that it would cease manufacture of Dengvaxia in 2025. There therefore remains an unmet need for a safe and efficacious dengue vaccine that can be used programmatically without pre-vaccination testing.

Dengue homotypic immunity is thought to be lifelong, whereas heterotypic cross-immunity between serotypes following primary exposure is short-lived and titer dependent^5,6^. High neutralizing antibody titers against dengue are associated with protection, and low-to-moderate antibody titers are associated with an increased risk of severe disease and hospitalization^7–9^. However, an exact titer for protection has not yet been identified, and it is expected that this will depend on the assay used, the infecting serotype (and potentially genotype), and most likely, an individual’s prior exposure to other serotypes and related flaviviruses^10,11^. Nevertheless, vaccine-induced antibody titers correlate with protection at the population level^12^ and are good predictors of disease risk^13^, leading the World Health Organization (WHO) to recommend neutralizing antibody titers as an immunogenicity metric for second-generation dengue vaccines^14^.

Qdenga, a second-generation vaccine developed by Takeda Pharmaceuticals, has recently been approved for use in several countries, including Brazil, where vaccine rollout began in 2024 (ref. ^15^). Qdenga is a tetravalent chimeric live-attenuated vaccine using DENV2 as the backbone for all four serotype components, but substituting DENV1, 3 and 4 pre-membrane and envelope proteins for those serotypes^16^. Qdenga’s efficacy was evaluated in a multi-country phase III trial across Asia and South America that enrolled approximately 21,000 participants aged 4–16 years, who were randomized 2:1 to receive two doses of Qdenga or placebo, 90 days apart^17^. Building on the experience with Dengvaxia, vaccine efficacy (VE) was evaluated stratified by baseline serostatus before vaccination, infecting serotype, age and disease outcome (defined as symptomatic dengue and hospitalization) for all trial participants, at 12 months^17^, 18 months^18^, 24 months^19^, 36 months^20^ and 54 months^21^ after the second dose.

From 1 to 57 months post-first dose, the average VE in the safety population (individuals receiving at least one dose of the vaccine) was estimated at 61.2% (95%CI: 56.0–65.8) against symptomatic virologically confirmed dengue (VCD) and 84.1% (95%CI: 77.8–88.6) against hospitalized VCD over all serotypes and baseline serostatuses^21^. However, VE waned over time, from an average of 80.2% (95%CI: 73.3–85.3) against symptomatic VCD in the per-protocol population (individuals without any major protocol violations, including not receiving both doses of the correct assignment of Qdenga or placebo) in year 1 to 44.7% (95%CI: 32.5–54.7) in year 3. VE also varied by serotype and baseline serostatus, with higher VE in seropositive individuals and against DENV2^20^. Consistent with the VE estimates, neutralizing antibody titers induced by Qdenga were highest and more durable in seropositive individuals and against DENV2^21^, with specific antibody^22^ and T cell^23,24^ responses most strongly elicited against the DENV2 backbone virus.

In baseline seronegative individuals, the phase III trial showed no statistically significant evidence of protection against DENV3 and DENV4, with average VE estimates up to 57 months post-first dose of −15.5% (95%CI: −108.2 to 35.9) and −105.6% (95%CI: −628.7 to 42.0), respectively^21^. During this period, point estimates of VE against hospitalization following a DENV3 infection were negative in seronegative recipients (VE −87.9%, 95%CI: −573.4 to 47.6), although six of 11 DENV3 hospitalizations in seronegative vaccinees occurred in Sri Lanka, where hospitalization tends to be more frequent than in other countries^21^. Additionally, there were two cases of severe dengue in the seronegative vaccine group, both DENV3, and none in the placebo group. Using the WHO 1997 criteria for dengue hemorrhagic fever, there were two cases in the seronegative placebo group (DENV1 and DENV3) and four in the vaccine group (all DENV3). This raises the question of whether these uncertain estimates represent a weak signal of vaccine-associated disease enhancement (defined as an increased risk of symptomatic disease or hospitalization in the vaccine group compared with the control group) in seronegative recipients for DENV3 and DENV4, despite the lack of statistical significance.

While the published VE estimates for Qdenga provide estimates of how VE varies by serotype, age, serostatus and over time, most such estimates are available as stratified by only two of those variables. For instance, no efficacy estimates have been published by both serotype and age to date. To support optimal deployment, it is also important to estimate the public health impact of Qdenga vaccination and evaluate its suitability across different transmission settings. Here, we address these knowledge gaps. We first develop a survival model calibrated to published phase III data to infer how antibody titer dynamics can be translated into estimates of protection. Second, we embed this VE model into a previously published dengue transmission model^4^ to simulate the potential public health impact of programmatic Qdenga use, estimating impacts at both the population and individual levels. This work informed the WHO Scientific Advisory Board of Experts recommendations on dengue vaccines^25^ and the latest WHO position paper on dengue vaccination^26^.

## Results

### Vaccine efficacy against disease and hospitalization

**Fig. 1:**
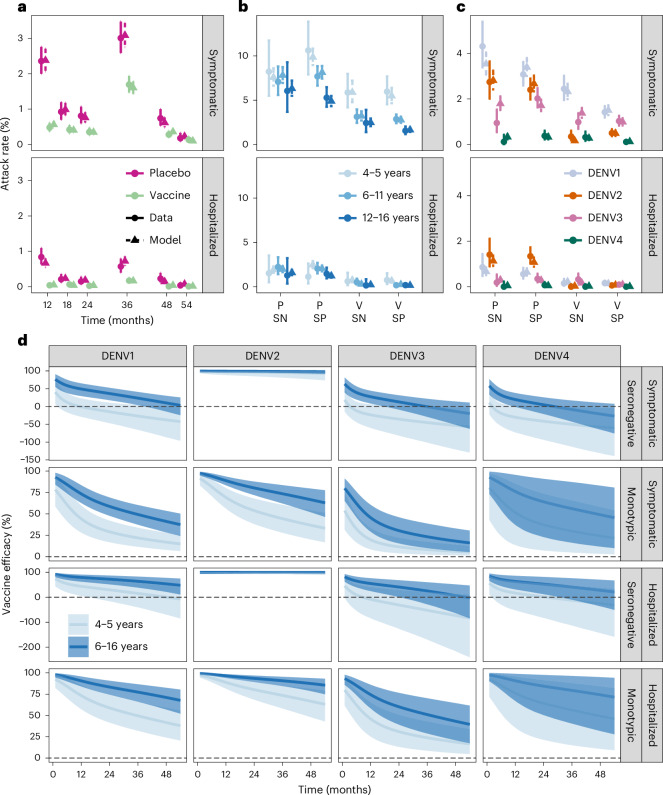
Attack rate and vaccine efficacy estimates. **a**–**c**, Observed and estimated attack rates of symptomatic virologically confirmed disease and hospitalizations in the phase III clinical trial by trial arm and time (**a**), age, serostatus and trial arm (**b**), and serotype, serostatus and trial arm (**c**). Note that the plotted resolution is lower than the data used in the model calibration. The modeled attack rates show the mean (triangle) and 95% credible interval (CrI, dashed line) of the posterior distribution (*n* = 20,000 samples). The observed attack rates show the mean (circle) and 95% exact binomial confidence interval (solid line). **d**, Estimated vaccine efficacy by serotype, serostatus and age group, against symptomatic disease and hospitalization. The solid line represents the mean of the posterior distribution, and the shaded area represents the 95%CrI of the posterior distribution (*n* = 20,000 samples). The dashed horizontal line marks 0 efficacy. Vaccine efficacy in multitypic individuals is shown in Supplementary Fig. 8. P, placebo arm; SN, seronegative; SP, seropositive; V, vaccine arm.

We fitted a Bayesian cohort survival model to all published Qdenga phase III clinical trial data including symptomatic and hospitalized cases (Supplementary Figs. 1a and 2). The model extends the correlates of protection model first proposed by Khoury et al.^27^ for SARS-CoV-2, enabling us to link the neutralizing antibody titers induced by Qdenga (Supplementary Fig. 3) to the disease risk in the vaccine arm compared with the placebo group. Of the 31 model variants explored (Supplementary Fig. 4), the optimal model following model selection reproduced the symptomatic case and hospitalization attack rates observed over the 54 months post-second dose by inferring the relative serotype-, serostatus-, outcome- and age-specific titers required for protection, and testing the hypothesis of potential vaccine-associated enhancement in seronegative individuals. We fit the model to the reported number of cases stratified by trial arm, age group, serotype and baseline serostatus at the finest granularity allowed by the published data (Fig. 1a–c and Supplementary Figs. 5–7). Posterior parameter estimates are listed in Supplementary Table 1.

We estimate high VE against symptomatic and hospitalized DENV2, regardless of age or serostatus (Fig. 1d and Supplementary Fig. 8). In seropositive individuals we estimate more moderate protection against symptomatic disease and hospitalization for the other serotypes. Conversely, for seronegative vaccinated subjects in the 4–5 years age group, we estimate an enhanced risk of symptomatic disease (negative average VE) following DENV1, DENV3 and DENV4 infection, starting from 3–12 months after the second dose, albeit credible intervals (CrIs) always include zero. For DENV3 and DENV4 we also estimate an enhanced risk of hospitalization from 9 months and from 26 months after the second dose in the seronegative 4–5 year age group, respectively. For seronegative children over 6 years old, we estimate positive average VE against symptomatic disease and hospitalization for DENV1 for the entire follow-up period (54 months post-second dose) but negative average VEs were estimated for DENV3 and DENV4 from approximately 3 years post-second dose. The large uncertainties around the VE estimates against hospitalization and for DENV3 and DENV4 are due to the small case numbers observed during the trial. No DENV4 hospitalizations were observed in seronegative individuals in either trial arm, hence the efficacy estimates obtained in the main analysis were informed by the DENV4 symptomatic cases and the DENV1, DENV2 and DENV3 hospitalizations (Methods).

Supplementary Fig. 9 shows the VE estimates as a function of neutralizing antibody titers. In seronegative individuals, for any given titer, we estimate the same VE regardless of the serotype, whereas in monotypic individuals the VE depends on the serotype and the titer. The serostatus-specific mean titers providing 50% protection (in the absence of enhancement) are listed in Supplementary Table 1, with the titers conferring 50% VE to seronegative individuals listed in Supplementary Table 2. Supplementary Fig. 10 shows the VE projections up to year 15 post-vaccination, assuming the same efficacy decay rate estimated for the trial period. VE against symptomatic and hospitalized dengue remains moderate to high against DENV2, especially for seronegative individuals. For the other serotypes, we estimate that the vaccine offers little or no protection against disease or hospitalization in seropositive individuals by year 15. The model predicts that starting from years 5–8 post-second dose, seronegative vaccinated individuals would experience an enhanced risk of symptomatic disease and hospitalization following a DENV1, DENV3 and DENV4 infection, irrespective of age. Note, however, that if a seronegative vaccinated individual has a breakthrough infection, we assume that their protection is the same as that of a vaccinated monotypic individual.

Our model explicitly included a parameter determining the maximum potential level of vaccine-associated disease enhancement, or, in other words the minimum VE, estimated at −84% (95%CrI: −13 to −184). The Bayes factor for the comparison of our model with and without disease enhancement is 13, implying strong but not irrefutable evidence for enhancement (Supplementary Fig. 11) regardless of the prior distribution used, even though the magnitude of the estimated enhancement parameter was influenced by the choice of the prior distribution.

The results of sensitivity analysis on the period of heterotypic immunity, the performance of the test used to classify baseline serostatus, and whether post-secondary infections can lead to clinical disease, all showed similar results to those obtained in the main model (Supplementary Fig. 12). Additional sensitivity analyses showed that assumptions about serotype-specific titers for protection and hospitalization risk could slightly shift VE estimates by serostatus, serotype and outcome compared with the main analysis (Methods and Supplementary Fig. 12). To validate the model, we fitted it to 20 sets of simulated data. All parameters were estimated well (that is, the distribution of the estimated parameter values in blue overlaps with the distribution of the values used to simulate the data in red, Supplementary Fig. 13).

### Population impact of routine Qdenga vaccination

To estimate the impact of Qdenga vaccination, we integrated our fitted VE model (Fig. 1d) into the multi-strain stochastic compartmental model of dengue transmission previously used to investigate the potential impact of Dengvaxia^4^. We explored four hypotheses of the vaccine’s mode of action, combining two assumptions regarding protection (against disease only, VS (Fig. 1d) or also against infection, VI), with two assumptions about the duration of VE decay (up to 5 years, D5, or 15 years, D15) (Supplementary Fig. 10). Given the limited data available^28^, we assumed that protection against infection requires a higher titer than protection against symptomatic disease and that vaccine-associated enhancement applies only to clinical outcomes^8^ (Methods and Supplementary Fig. 14). We examine a range of transmission settings with different forces of infection (FOI, characterized by the average seroprevalence in 9-year-olds (SP9), in line with our previous work^4^). For each transmission setting, vaccine mode of action, coverage level (20%, 40%, 60%, 80%), age of vaccination (6–12 years) and population demography (Brazil and the Philippines), we sampled 200 posterior parameter estimates from our VE model (Fig. 1d) and for each such posterior sample we ran 50 simulations of the dengue transmission model, giving 10,000 simulations in total per scenario (Supplementary Fig. 1b).

**Fig. 2:**
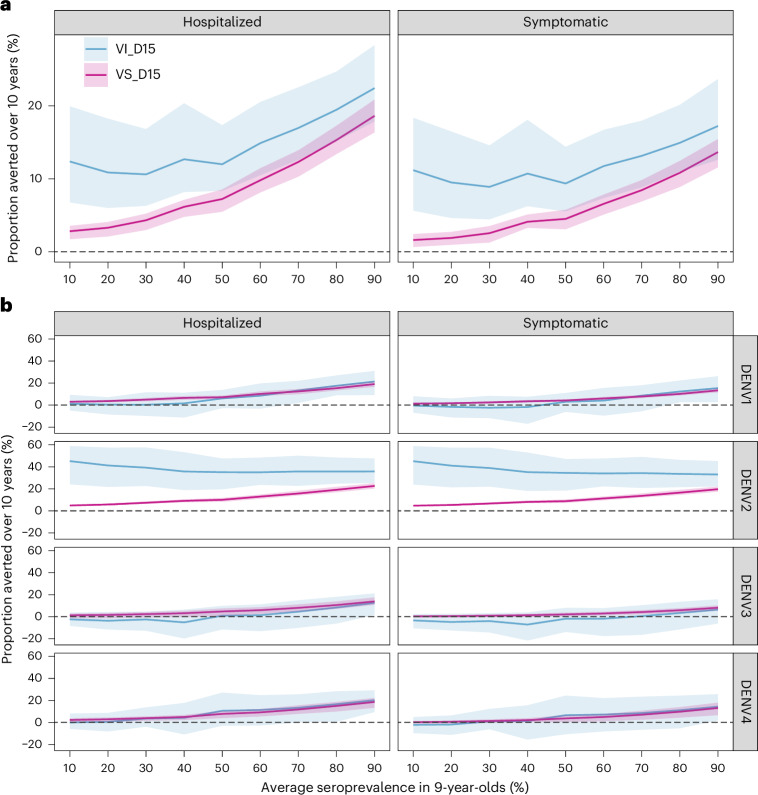
Population-level impact of vaccination. **a**,**b**, Cumulative proportion of hospitalized and symptomatic cases averted over 10 years by transmission setting (expressed as the average seroprevalence in 9-year-olds), assuming efficacy against infection and disease (VI, blue) or only against disease (VS, pink) decaying for 15 years (D15), using 80% coverage across 10 years and the Brazilian demography over all serotypes (**a**) and by serotype (**b**). The dashed horizontal line marks 0 cases averted. The solid lines represent the mean and the shaded regions represent the overall uncertainty (95%CrI) derived from *n* = 10,000 simulations (that is, 200 posterior distribution samples × 50 stochastic simulations per sample, see Methods for details).

Figure 2a shows the population impact, summarized as the total proportion of cases averted over 10 years, assuming the Brazilian demography, 80% vaccination coverage in 6-year-olds, and VE waning for 15 years (scenarios VS_D15 and VI_D15). Supplementary Figs. 15–17 show that the expected impacts assuming the Philippines’ demography and VE waning for 5 years are similar to those for the scenario presented in the main analysis (Fig. 2a). Under the VS assumption, the population-level impact increases as the transmission intensity increases, while under the VI assumption the population-level impact is similar across all transmission settings with <50% SP9 (Fig. 2a). Regardless of the transmission setting or VE against infection, the population impact is modest, with the mean proportion of symptomatic cases prevented over 10 years ranging from 1.6% to 13.7% under the VS scenario and from 8.9% to 17.2% under the VI scenario, depending on transmission intensity. The mean proportion of hospitalizations averted is slightly higher, rising to 22.4% (95%CrI: 17.8–28.3) assuming the VI scenario in the highest transmission settings.

Figure 2b shows the population impact by serotype, highlighting the potential for small negative impacts against DENV1, DENV3 and DENV4 in low-to-moderate transmission settings. In other words, in low-to-moderate transmission settings, most of the positive impact of vaccination at the population level in the VI and VS scenarios can be attributed to preventing DENV2 cases (Fig. 2b). We estimate the maximum excess of symptomatic cases under the VI scenario to be −0.5% (95%CrI: −6.9 to 8.0) to −2.4% (95%CrI: −11.8 to 8.4) for DENV1, −1.8% (95%CrI: −17.6 to 7.7) to −7.2% (95%CrI: −21.7 to 3.9) for DENV3, and −1.9% (95%CrI: −11.3 to 6.3) to −2.2% (95%CrI: −9.7 to 4.9) for DENV4, depending on the transmission settings. Also under the VI scenario, we estimate a risk of excess hospitalizations only for DENV3, ranging from −2.4% (95%CrI: −8.4 to 3.7) to −5.2% (95%CrI: −19.8 to 6.4). Under the VS scenario, the average population impacts are always positive, although the lower bounds of the 95%CrI are sometimes negative. In settings with 60% SP9, we estimate that 2.9% (95%CrI: 1.1–4.5) and 4.9% (95%CrI: 1.1–7.7) of DENV3 and DENV4 symptomatic cases would be averted under the VS scenario, compared with −1.8% (95%CrI: −17.6 to 7.7) and 7.1% (95%CrI: −8.2 to 22.1) of cases under the VI scenario.

**Fig. 3:**
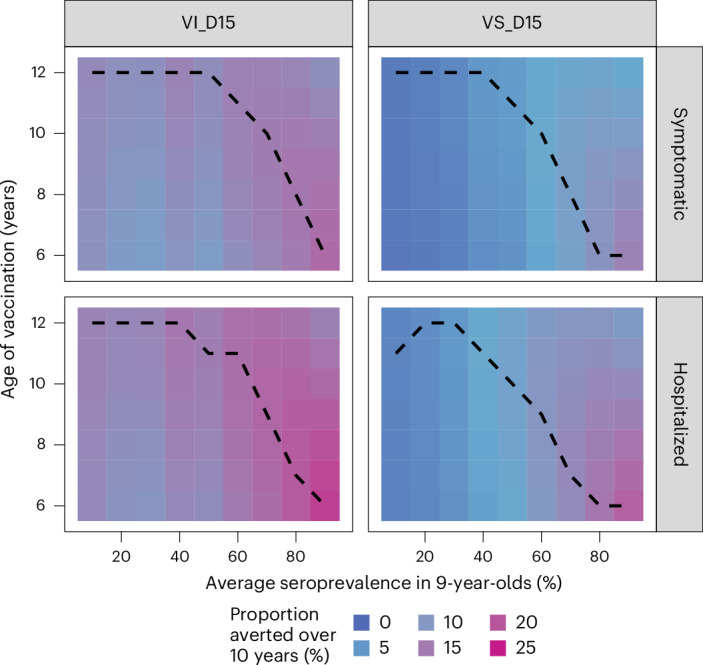
Impact of the age at vaccination on the proportion of cases averted at the population level by transmission setting. The colors show the cumulative proportion of hospitalized and symptomatic cases averted over 10 years by transmission setting (expressed as the average seroprevalence in 9-years-olds), and vaccine mechanism assuming vaccination at ages 6–12 years and the Brazilian demography. The dashed line shows the optimal age of vaccination for each transmission intensity. VI_D15, scenario assuming efficacy against infection and disease decaying for 15 years post-vaccination; VS_D15, scenario assuming efficacy only against disease decaying for 15 years post-vaccination.

Figure 3 and Supplementary Fig. 18 present the population impact by age of vaccination in Brazil and the Philippines, respectively. In low-to-moderate transmission settings the impact is relatively insensitive to vaccination age (given that we did not consider ages >12 years for vaccination) (Fig. 3). In moderate-to-high transmission settings, the optimal age of vaccination decreases from age 11 to 6 as SP9 increases from 50% to 90% (Fig. 3). Under the VS assumption, reductions in vaccination coverage from 80% to 20% reduce impact proportionately (that is, by 75%) regardless of the transmission setting, whereas under the VI assumption, reducing coverage from 80% to 20% reduces the impact by between 68% to 74%, depending on the transmission setting (Supplementary Figs. 19 and 20).

We ran sensitivity analyses on the effects of the choice of VE model, the period of heterotypic immunity, the seasonality of dengue transmission, and the relative transmissibility of different serotypes on the population impact (see Methods for more details). The impact estimates obtained in these sensitivity analyses are consistent with the results obtained in the main analysis, especially under the VS scenario (Supplementary Fig. 21). The sensitivity analyses applied to the VI scenario tended to result in slightly lower central estimates of population impacts compared with the main analysis.

### Individual-level impact of routine Qdenga vaccination

**Fig. 4:**
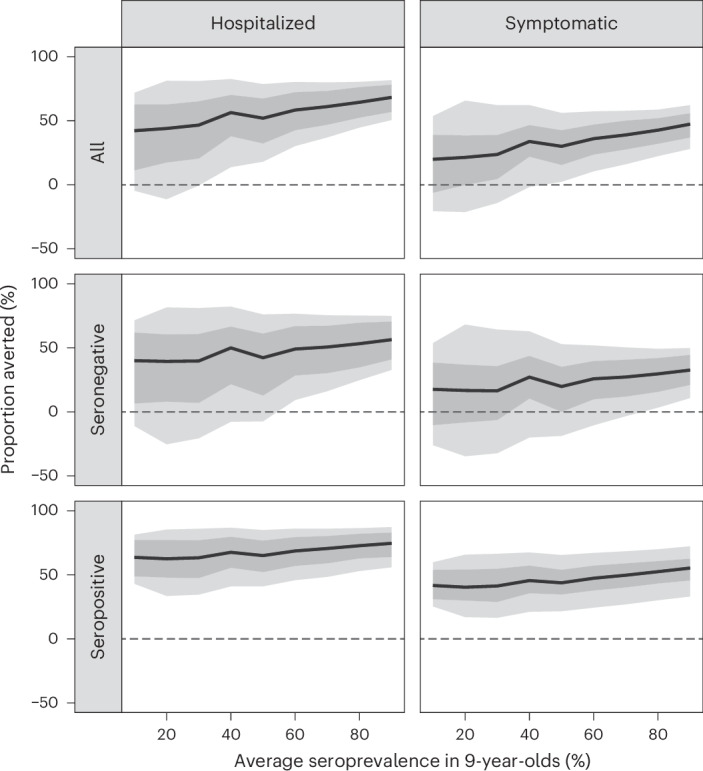
Individual-level impact of vaccination. Proportion of hospitalized and symptomatic cases averted in the first vaccinated cohort of 6-year-olds over 10 years by transmission setting, expressed as the average seroprevalence in 9-year-olds assuming a vaccination coverage of 80% using model VS_D15 (efficacy against disease decaying for 15 years post-vaccination) and the Brazilian demography. The impact is shown overall (all) and among baseline seropositive and seronegative vaccinees. The dashed horizontal line marks 0 cases averted. The solid lines represent the mean; the light shading represents the overall uncertainty (95%CrI), derived from *n* = 10,000 simulations (that is, 200 posterior distribution samples × 50 stochastic simulations per sample, see Methods for details); and the dark shading represents the parameter uncertainty (95%CrI), derived from 200 posterior distribution samples.

**Fig. 5:**
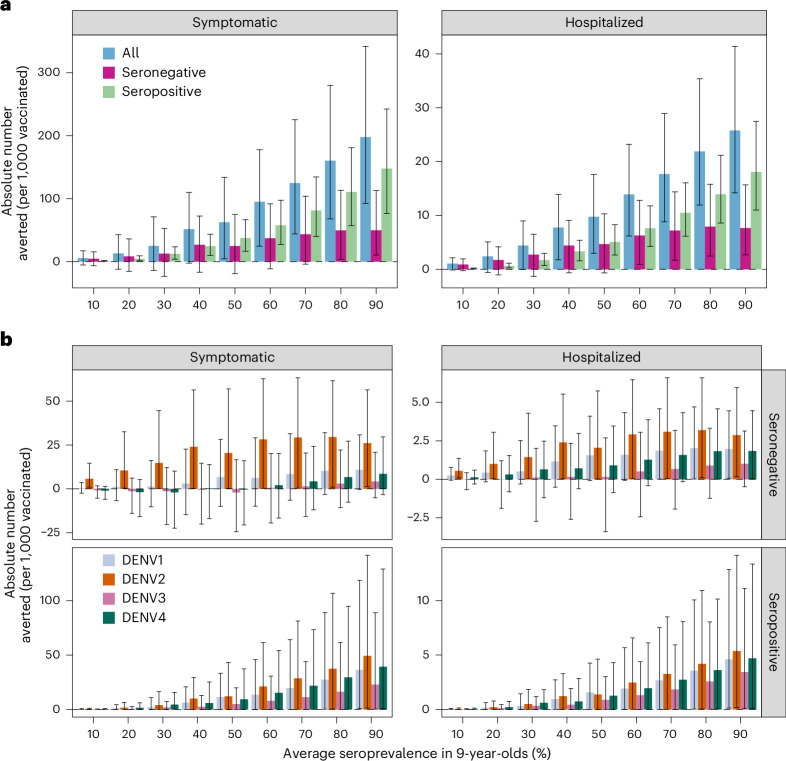
Cases averted by vaccination per 1,000 vaccinated children. Absolute number of symptomatic cases and hospitalizations averted over 10 years post-second dose in the first vaccinated cohort of 6-year-olds per 1,000 fully vaccinated persons by transmission setting, expressed as the average seroprevalence in 9-year-olds, assuming model VS_D15 (efficacy against disease decaying for 15 years post-vaccination), the Brazilian demography and that 9% of symptomatic cases are hospitalized. **a**, Cases averted in the entire vaccinated cohort (all; blue), and among the baseline seropositive (green) and baseline seronegative (pink) vaccinees. **b**, Cases averted by serotype and serostatus. The bars represent the mean, and the error bars represent the overall uncertainty (95%CrI) derived from *n* = 10,000 simulations (that is, 200 posterior distribution samples × 50 stochastic simulations per sample, see Methods for details).

While the overall average population impact is always positive, the individual benefits and risks of vaccination (measured as the proportion of cases averted in the first vaccinated cohort over 10 years) present a more complex picture (Fig. 4 and Supplementary Figs. 22–24). The mean individual impact is positive, with 20–47% and 42–68% of symptomatic cases and hospitalizations averted in vaccinated individuals over 10 years, respectively. Seropositive individuals always benefit from vaccination, with 40–55% and 63–75% of symptomatic and hospitalized cases averted, respectively. Conversely, while the mean impact is positive for seronegative vaccinees, negative impacts in low-to-moderate transmission settings are possible, as demonstrated by the negative lower bound of the 95% uncertainty intervals (Fig. 4), reflecting both the uncertainty in the VE estimates (95%CrI) (Fig. 1d) and in the circulating serotypes across the simulations (stochastic uncertainty; see Methods for details). Figure 5a shows that overall, vaccination is expected to avert 95 cases (95%CrI: 25–178) and 14 hospitalizations (95%CrI: 6–23) (assuming that 9% of symptomatic cases are hospitalized) per 1,000 vaccinated individuals in the 60% SP9 transmission setting, primarily due to the prevention of cases in seropositive individuals. In seronegative individuals, symptomatic cases are averted almost entirely against DENV2 (Fig. 5b), regardless of the transmission setting. Negative DENV3 impacts are more likely than positive impacts in seronegative individuals in transmission settings with <60% SP9, although the maximum average number of excess cases is low, with up to −2 (95%CrI: −24 to 17) and 0 (95%CrI: −3 to 2) DENV3 symptomatic cases and hospitalizations averted per 1,000 vaccinated, under the VS scenario (and similar under the VI scenario).

### Population screening

A potential strategy to mitigate the risk in seronegative recipients (Fig. 4) is pre-vaccination serological screening, as recommended for Dengvaxia^14^. Assuming that everyone eligible for vaccination is screened with a diagnostic test with 94.7% specificity and 89.6% sensitivity^29^, and either the VI or VS mode of vaccine action, we find that pre-vaccination screening reduces the population impact of vaccination on symptomatic disease and hospitalizations by 29–33% in the highest transmission setting and 82–85% in the lowest transmission setting (Supplementary Figs. 25 and 26). As a result, the proportions of symptomatic and hospitalized cases averted over 10 years are <15% across all transmission settings when implementing pre-vaccination screening. This is driven by the loss of protection against DENV1 and DENV2 in seronegative recipients (Fig. 5b). The impact of pre-vaccination screening on vaccinated individuals is shown in Supplementary Figs. 27 and 28. Overall, screening increases the individual impact, given that a higher proportion of the vaccinated individuals are seropositive. In the seronegative individuals who are vaccinated due to imperfect test specificity, screening does not change the proportion of cases averted.

### Impact of vaccination on serotype dynamics

**Fig. 6:**
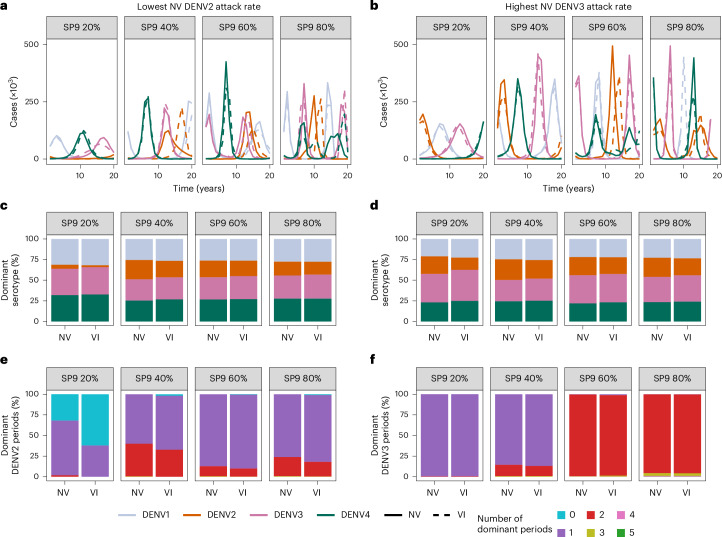
Impact of vaccination on serotype dynamics. **a**–**f**, For the *n* = 1,000 simulations with the lowest DENV2 burden (**a**,**c**,**e**) and the highest DENV3 burden (**b**,**d**,**f**) in the absence of vaccination, across transmission settings (SP9, average seroprevalence in 9-year-olds) with (VI) and without (NV) vaccination, we show example serotype-specific (color) transmission dynamics with (dashed line) and without vaccination (solid line) (**a**,**b**), the mean proportion of time that each serotype (color) is dominant (**c**,**d**), and the proportion of simulations in which DENV2 (**e**) and DENV3 (**f**) become the dominant serotype for the specified number of seasons (colors), over a 20-year period since the start of vaccination.

Given the imbalanced serotype-specific efficacy profile (Fig. 1d) and population impacts (Fig. 2b), we investigated the extent to which vaccination assuming the VI scenario (in which vaccination affects transmission) could affect serotype-specific dengue dynamics. Specifically, we tested whether the introduction of Qdenga could eliminate DENV2 in settings with low DENV2 circulation and favor the circulation of the other serotypes; and whether it could increase the dominance of DENV3 in settings with high DENV3 circulation. We investigated changes in the serotype-specific dynamics among the 1,000 simulations that, in the absence of vaccination, had the lowest and highest DENV2 and DENV3 incidences, respectively. Figure 6a,b shows example transmission dynamics with and without vaccination and demonstrates how, under the VI assumption, vaccination is expected to change outbreak peaks in both timing and magnitude, especially in settings with higher transmission intensities.

In low-to-moderate transmission settings with limited DENV2 circulation, we expect vaccination to reduce DENV2 dominance, with a lesser impact in higher transmission settings (Fig. 6c,e). Conversely, in low transmission settings with high DENV3 circulation, the introduction of Qdenga may increase DENV3 dominance, but in high transmission settings this effect is modest (Fig. 6d), and the number of DENV3-dominant periods over 20 years remains largely unchanged by vaccination (Fig. 6f). Taken together, these results suggest that under the VI mode of vaccine action, routine vaccination in moderate-to-high transmission settings would only minimally alter the serotype-specific dynamics that would be observed in the absence of vaccination.

## Discussion

By combining correlates of protection model of VE with a mechanistic dengue transmission model, we show that Qdenga has an efficacy profile and predicted public health impact that varies with age, serostatus and infecting serotype. We estimate that Qdenga is highly protective against DENV2 regardless of serostatus, and moderately protective against the other three serotypes in seropositive individuals, with evidence of potential enhancement in DENV3 and DENV4 disease risk in seronegative subjects. Notably, we found that this risk is greatest in seronegative children aged 4–5 years at vaccination. Despite this imperfect efficacy profile, our estimates suggest that the introduction of Qdenga could reduce the burden of hospitalized dengue in high transmission intensity settings by up to approximately 20% over the first 10 years of routine programmatic use. These findings support the recent WHO recommendations on the use of Qdenga in children aged 6 years and above^25,26^.

Qdenga VE estimates stratified by serotype and age have not yet been reported by the phase III trial investigators^17–21^. However, our model-derived estimates agree with early clinical studies and previous work on the functional immune response elicited by Qdenga, which suggested that DENV2 is the dominant replicating serotype and driver of homotypic immunity^22,23,30^. We find that Qdenga protects against symptomatic disease caused by DENV1, DENV3 and DENV4 in seronegative children aged 4–5 years for 3–12 months, after which we estimate a potential risk of disease enhancement. In seronegative children aged 6–16 years, we estimate the initial period of partial protection to be at least 27 months. Notably, given that we assume that the order of infection and vaccination does not affect immunity (that is, a seronegative vaccinee with a single breakthrough infection achieves the same protection level as a monotypic vaccinee), any potential enhanced disease risk estimated here applies only until a seronegative individual’s first breakthrough infection. Therefore, in high transmission settings, not only are more individuals seropositive at vaccination, but seronegative individuals are also more likely to experience a breakthrough infection sooner, when VE is higher, and the risk of disease enhancement is lower.

We cannot rule out a potential risk of enhancement in any seronegative subjects, but we found that VE was higher and the risk of enhancement lower in children 6 years and over compared with those under 6, independent of their serostatus. Age dependencies in VE were also found in the analysis of the Dengvaxia clinical trial data^31,32^, and although the reasons for this age dependency are not yet fully understood, these results suggest reduced antibody and cell-mediated memory responses to vaccines in young children^33,34^.

The uncertainty around VE and potential risks for seronegative recipients highlight the need for continued monitoring of Qdenga’s efficacy profile and collection of additional data in post-licensure studies^25,35^, including case-cohort studies in endemic countries, focusing on DENV3 and DENV4 hospitalizations and severe cases in seronegative individuals. The collection of serological data through future trials would also address gaps in our understanding of the vaccine’s efficacy against infection, which we have shown is important for estimating the population-level impact of dengue vaccines. Finally, Takeda are currently evaluating the use and timing of an additional booster dose^26^, and it will be important to assess whether an extra vaccine dose might mitigate the potential risks that we have identified.

Our analysis shows that population impact and individual benefit–risk depend on setting-specific transmission intensity and dominant circulating serotypes. In low transmission settings the average impact in seronegative individuals is positive, but the positive impact is driven almost entirely by preventing DENV2 cases and is counterbalanced by lower (and potentially negative) impacts against DENV3 and DENV4. However, in higher transmission intensity settings (>60% SP9), the lower 95% credible bound on individual impact remains positive. Avoiding potentially negative individual impacts therefore requires Qdenga vaccination strategies targeted at high transmission settings, using recent FOI estimates^36^ and optimized vaccination ages. Pre-vaccination screening^37^ is expected to reduce the (already modest) population impact by approximately 30–80% depending on the transmission setting, and considering test limitations^38^, additional costs, logistical challenges and the experience with Dengvaxia^39^; pre-vaccination screening for Qdenga is currently not recommended by the WHO^26^.

In early 2024 Brazil became the first country to roll out Qdenga^15^ and our estimates suggest that, assuming 80% coverage, in a high transmission setting (60% SP9) Qdenga could avert 95 (95%CrI: 25–178) symptomatic cases and 14 (95%CrI: 6–23) hospitalized cases, per 1,000 children vaccinated. Careful monitoring of the Brazilian program will be important to evaluate program effectiveness, impact and safety.

Given the general lack of serotype-specific transmission intensity estimates, we assumed that all serotypes were approximately equally transmissible in the main model. However, during Qdenga’s phase III trial, spanning 4.5 years across eight countries in Southeast Asia and South America, the four serotypes (DENV1, DENV2, DENV3 and DENV4) accounted for 42.9%, 31.4%, 21.8% and 3.9% of cases, respectively. In a sensitivity analysis, we assumed that DENV1 and DENV2 are more transmissible than DENV3 and DENV4. This resulted in slightly lower impacts than in the main model because a more transmissible DENV2 is harder to control, reducing the vaccine’s impact against that serotype. Additionally, assuming lower transmissibility of DENV3 and DENV4 reduces the population-level impacts due to lower population immunity against these serotypes, meaning that a higher proportion of vaccine recipients are seronegative at vaccination. More generally, we note that DENV3 has recently re-emerged in several South American and Asian countries after its absence over several years, suggesting a need for caution in assuming that past serotype dominance trends will continue in the future. There is a need to better characterize the fundamental transmissibility and severity of the four serotypes. Critically, despite the estimated serotype efficacy imbalance, our modeling suggests that even widespread programmatic use of Qdenga will, at most, cause minor changes in patterns of serotype dominance in moderate-to-high transmission settings.

Although modeling cannot substitute for the lack of data, our biologically motivated approach to VE modeling enabled us to share model parameters across strata, increasing statistical power, especially for DENV4, for which there were limited cases and very few hospitalizations. Although absolute dengue correlates of protection are yet to be identified, our analysis indicates that mean antibody titer kinetics can explain the efficacy of Qdenga, suggesting that neutralizing titers are a surrogate marker for protection (or risk) from symptomatic and hospitalized dengue at the population level^8,9,13^. However, there are several assumptions and limitations to our VE model. We needed to assume different threshold titers for protection for seronegative and seropositive vaccine recipients, suggesting that qualitative differences in the humoral response and potentially other immune functions, such as cellular immunity, play an important role^22,23,40^. The serotype-specific titers conferring 50% protection estimated in this study are assay (and probably laboratory) specific and depend on the reference virus used in the study. In other words, while our estimates represent correlates of protection for the clinical trial, these do not represent universal correlates of protection in other settings.

Without individual-level data we could not investigate the link between individual antibody trajectories and vaccine-induced protection, nor could we investigate how boosting of antibody titers due to subclinical infections and homotypic dengue re-exposure during the phase III trial^13^ affected our VE estimates. Notably, our VE model assumes that efficacy decays monotonically as titers decay, whereas previous work on natural infections showed that clinical risk peaks at low-to moderate titers and decreases with very low or no titers^8^. While our model may overestimate the risk of disease in seronegative vaccinees with very low or no antibody titers, this overestimation of the risk applies only until the first breakthrough infection (when we assumed the same protection as a monotypic vaccinee). Additionally, we show that the population impact of vaccination is consistent when assuming VE decays for either 5 or 15 years before plateauing.

When estimating VE, we assumed a constant serotype-specific FOI within each trial reporting interval (which were between 6 and 12 months in duration). Although it is likely that the FOI changed seasonally within the reporting intervals, the lack of data at finer temporal resolution poses limitations to the extent to which we could relax this assumption. We were unable to account for the impact of cross-immunity from other flaviviruses^41–44^ on our VE estimates, although results from the clinical trial indicate that prior flavivirus vaccination did not impact Qdenga’s performance^45^. The use of hospitalization as a clinical endpoint is an imperfect surrogate measure for disease severity because protocols varied widely between countries, most noticeably in Sri Lanka, where 68% of cases in the placebo arm were hospitalized compared with the trial average of 25% (ref. ^20^). This, combined with country-specific heterogeneities in the circulating serotypes, meant that we were unable to account for different country-specific rates of dengue disease. Standardized trial definitions of dengue fever, severe dengue and hospitalization will be important for evaluating and comparing future dengue vaccines as new candidates are developed and licensed. Reporting trial data stratified by country would also help to address potential confounding and to estimate serotype-specific differences in severity.

The VE model developed in our study is easily adaptable to estimate and compare the efficacy of other dengue vaccines, such as the live-attenuated Butantan-DV vaccine currently under evaluation in Brazil^46^. The transmission model presented in this study can similarly be used to assess the potential impact of other dengue interventions and combinations of interventions, including new dengue vaccines, *Wolbachia*^47^ and antivirals^48^, supporting the WHO recommendation to consider dengue vaccination as part of an integrated strategy to control dengue^26^. It will be important to evaluate how combinations of interventions will affect transmission dynamics, and hence the impact of vaccination.

In conclusion, this study finds evidence of high efficacy of Qdenga vaccination against DENV2 and against the other serotypes in seropositive individuals, resulting in modest reductions in dengue cases and hospitalizations across different transmission intensity settings. Conversely, except for DENV2, we found evidence for a potential risk of enhancement in seronegative individuals, especially in those aged 4–5 years at vaccination. The analysis presented in this paper informed the recommendations recently published in the WHO position paper^26^ on dengue vaccination and demonstrates how modeling can help translate clinical trial data and complex efficacy profiles into population impact estimates, thereby optimizing vaccination deployment to minimize individual risk and maximize public health benefit.

## Methods

### Data

Published phase III clinical trial data are available for 12 months^17^, 18 months^18^, 24 months^19^, 36 months^20^ and 54 months^21^ after the second dose. From these publications we extracted the number of symptomatic VCD cases (${N}_{{{\rm{symp}}}}$) and the number of VCD cases leading to hospitalization (${N}_{{{\rm{hosp}}}}$) at the finest stratification available, in Excel (version 16.75).

Per-protocol outcomes and population sizes for the Qdenga phase III clinical trial are shown in Supplementary Fig. 2. ${N}_{{{\rm{symp}}}}$ was published by the trial arm $v$ (1 for placebo, 2 for vaccinated), classified baseline serostatus $b$ (−, seronegative; +, seropositive), and infecting serotype $k$ (1, 2, 3, 4 for DENV1–4, respectively), within each reporting interval $d$ (1, 1–12 months; 2, 13–18 months; 3, 19–24 months; 4, 25–36 months; 5, 37–48 months; 6, 49–54 months). The number of hospitalized cases, ${N}_{{{\rm{hosp}}}}$, was published by trial arm, baseline serostatus and infecting serotype at 1–24 months (safety set, individuals who received at least one dose of the vaccine or placebo), as well as for the last three reporting intervals. ${N}_{{symp}}$ and ${N}_{{{\rm{hosp}}}}$ were both published by trial arm, baseline serostatus and age group $j$ (1, 4–5 years; 2, 6–11 years; 3, 12–16 years), for the first four reporting intervals $d$ (that is, up to 36 months), and by age group and infecting serotype for reporting intervals 1–12 months and 13–24 months. Limited country-specific data were published, therefore all data and model estimates presented here are for the whole trial population across all countries.

The trial populations by baseline serostatus and trial arm were reported for each time interval, ${{N}_{{{\rm{pop}}}}}_{{bv}}(d)$, with the population further broken down by age group $j$ up to 36 months (Supplementary Fig. 2g,h). Given that only the first VCD case is reported in the trial, we right-censor cases at the end of each reporting interval $d$ and reduce the monitored population in the next time interval accordingly (that is, ${{N}_{\rm{{{surv}}}}}_{{bv}}(j,d)={N}_{\rm{{{po}{p}}}_{{bv}}}(j,d)-{N}_{\rm{{{{symp}}}}_{{bv}}}(j,d-1)$). Age-specific data were not published after month 36 of the trial, therefore we estimate the age-specific trial populations at 37–48 months and 49–54 months, assuming the same age-specific probability of loss to follow-up as the first 36 months of the trial.

### Cohort survival model of vaccine efficiency

To estimate VE we calibrated a Bayesian cohort survival model to the above data (Supplementary Fig. 1a). We assumed that an individual may be infected up to four times, and any infection could be symptomatic. We did not track further infections among individuals who had a detected infection. The model was solved at monthly time intervals (*t* = 1–54).

#### Fitting the initial conditions

Let ${p}_{{kj}}$ denote the probability of exposure to serotype $k(=\mathrm{1,2,3,4})$ in age group $j$ prior to the start of the trial $({t=T}_{0})$. We estimate ${p}_{k3}$ (the probability of exposure in 12–16 years). For the other two age groups, we estimate the cumulative probabilities of exposure to any serotype and scale serotype-specific probabilities to match the distribution of serotype-specific exposure in age group 3. Let ${h}_{k3}$ be the serotype-specific hazard of exposure in age group 3:

1 $${h}_{k3}=-\mathrm{ln}\left(1-{p}_{k3}\right)$$

The serotype-specific hazards in age groups $j=1$ and $j=2$ are therefore given by:

2 $${h}_{{kj}}=\frac{{h}_{k3}}{{\sum }_{k}{h}_{k3}}* -\mathrm{ln}\left(1-{p}_{j}\right)$$

Finally, the serotype-specific probabilities of exposure in age groups $j=1$ and $j=2$ are:

3 $${{p}_{{kj}}=1-e}^{{-h}_{{kj}}}$$

To aid identifiability of both the FOI and probability of symptoms, we constrain the probability of past exposure $p$ by the estimated FOI during the trial. Let ${\lambda }_{m}$ be the average monthly FOI during the trial (irrespective of serotype). The cumulative probability of exposure in the oldest age group ${p}_{m}$ is therefore:

4 $${p}_{m}=1-{e}^{\left(-14* 12* {\lambda }_{m}\right)}$$

where 14 is the mean age in the oldest age group and the factor of 12 reflects the time unit of months used in our analysis. We therefore set a beta prior for ${p}_{k3}$ with a mean ${p}_{m}$ and variance $\mathrm{var}=0.02* {p}_{m}(1-{p}_{m})$. A value of 0.02 was chosen to ensure that the prior was informative. We reparametrized the beta distribution to obtain standard shape parameters ${\rm{{shape}}}1$ and ${\rm{{shape}}}2$.

Let $$c$$ denote the number of serotypes to which an individual had been exposed (0, 1, 2, 3, 4). The probability that an individual has been exposed to serotypes $$\Omega$$ within set $${h}_{c}$$, defined as $${h}_{0}=\{\varnothing \}$$, $${h}_{1}=\left\{\left\{1\right\},\left\{2\right\},\left\{3\right\},\{4\}\right\}$$, $${h}_{2}=\left\{\left\{\mathrm{1,2}\right\},\left\{\mathrm{1,3}\right\},\left\{\mathrm{1,4}\right\},\left\{\mathrm{2,3}\right\},\left\{\mathrm{2,4}\right\},\{\mathrm{3,4}\}\right\}$$, $${h}_{3}=\left\{\left\{\mathrm{1,2,3}\right\},\left\{\mathrm{1,2,4}\right\},\right.$$$$\left.\left\{\mathrm{1,3,4}\right\},\{\mathrm{2,3,4}\}\right\}$$ and $${h}_{4}=\left\{\{\mathrm{1,2,3,4}\}\right\}$$ is given by:

5 $${{pE}}_{\Omega }\left(j{,T}_{0}\right)=\prod _{k\in \Omega }{p}_{{kj}}\prod _{{k}^{{\prime} }\notin \Omega }\left(1-{p}_{{k}^{{\prime} }j}\right)$$

Accounting for the imperfect test sensitivity, ${{\rm{sens}}}$, and specificity, ${{\rm{spec}}}$, of the microneutralization test used to classify individuals as seronegative (−) or seropositive (+) at baseline^49^, the probability of being classified as seropositive at baseline is:

6 $${p{\rm{SP}}}\left(j{,T}_{0}\right)=\left(1-{{\rm{spec}}}\right)p{E}_{{{\varnothing }}}\left(j,{T}_{0}\right)+{{\rm{sens}}}\left(\mathop{\sum }\limits_{c=1}^{4}\sum _{\Omega \in {h}_{c}}p{E}_{\Omega }\left(j,{T}_{0}\right)\right)$$

We estimate ${p{\rm{SP}}}\left(j{,T}_{0}\right)$ by fitting the number of baseline seropositive individuals by age group $j$ across both trial arms, ${N}_{{{\rm{SP}}}}\left(j,{T}_{0}\right)$ to the observed data, assuming a binomial distribution, and the observed number of participants ${N}_{{{\rm{pop}}}}\left(j,{T}_{0}\right)$ in age group $j$ over both the vaccine and placebo arms:

7 $$\begin{array}{c}{N}_{{{\rm{SP}}}}\left(j,{T}_{0}\right) \sim {{\rm{Binomial}}}\left({N}_{{{\rm{pop}}}}\left(j,{T}_{0}\right),{p{\rm{SP}}}\left(j{,T}_{0}\right)\right)\end{array}$$

#### Estimating vaccine efficacy

We used a modified form of the correlates of protection model first proposed by Khoury et al.^27^ (to model VE for SARS-CoV-2 vaccines) to link the imputed neutralizing antibody titer ${n}_{{ck}}\left(t\right)$ against serotype $k$ (Supplementary Fig. 3) with the risk ratio (comparing vaccinated and unvaccinated individuals) of symptomatic disease ${{{\rm{RR}}}_{{{\rm{symp}}}}}_{{cvkj}}\left(t\right)$:

8 $$\begin{array}{c}{{{\rm{RR}}}_{{{\rm{symp}}}}}_{{cvkj}}\left(t\right)=\frac{1+{L}_{{ck}}}{1+{\left(\frac{{n}_{{ck}}\left(t\right)}{{{e}^{{{\beta }_{{{\rm{symp}}}}}_{j}}* n}_{{50}_{{ck}3}}}\right)}^{{w}_{{ck}}}}\,\,\,{{\rm{if}}\; v}=2\end{array}$$

By definition, ${{{\rm{RR}}}_{{{\rm{symp}}}}}_{{cvkj}}=1$ if $v=1$. Here, ${L}_{{ck}}$ is the maximum potential vaccine-induced disease enhancement with serotype $k$ associated with vaccination. This is assumed to be zero for all individuals with one or more previous DENV exposures ($1\le c\le 4$), such that the maximum ${{\rm{RR}}}_{{symp}}$ is 1 for all baseline seropositive vaccinees. We estimate ${L}_{{ck}}$ (≥0) for vaccinated individuals with no prior DENV exposure ($c=0$), such that their maximum ${{\rm{RR}}}_{{{\rm{symp}}}}$ may be >1. The parameter ${n}_{{50}_{{ck}3}}$ represents the neutralizing titer conferring 50% protection against disease (in the absence of enhancement) for the oldest age group $(j=3)$, which is scaled by ${e}^{\;{{\beta }_{{{\rm{symp}}}}}_{j}}$ for individuals in the younger age groups, $j\in \{\mathrm{1,2}\}$. Finally, ${w}_{{ck}}$ controls the steepness of the relationship between neutralizing titer and the risk ratio.

Similarly, the risk of being hospitalized with dengue for a participant in the vaccination arm of the trial relative to one in the control arm is:

9 $${{{\rm{RR}}}_{{{\rm{hosp}}}}}_{{cvkj}}(t)=\frac{1+{\tau }_{k}* {L}_{{ck}}}{1+{\left(\frac{{n}_{{ck}}\left(t\right)}{{e}^{-{\alpha_{{ck}}}}* {e}^{{{\beta }_{{{\rm{hosp}}}}}_{j}}* {n}_{{50}_{{ck}3}}}\right)}^{{w}_{{ck}}}}\,\,\,{{\rm{if}}\,v}=2$$

This is the same model as used for the symptomatic VCD endpoint in equation (8) above with two modifications: first, the maximum level of vaccine-induced enhancement for symptomatic VCD, ${L}_{{ck}}$, is scaled by the multiplier ${\tau }_{k}$; and second, the neutralizing titer that gives 50% protection from hospitalization, ${n}_{50}$ is assumed to be proportional to that for VCD, but scaled by the factor ${e}^{-{\alpha }_{{ck}}}$. In addition, the variation of efficacy with age (represented by the parameter ${{\beta }_{{{\rm{hosp}}}}}_{j}$) is allowed to be different from that for the symptomatic VCD endpoint.

Note that because ${{\rm{RR}}}_{{{\rm{symp}}}}$ and ${{\rm{RR}}}_{{{\rm{hosp}}}}$ are dependent on the cumulative number of DENV exposures at month $t$, not the number of exposures prior to vaccination, we are assuming that the order of vaccination and infection does not matter. Therefore, for example, a baseline monotypic vaccinee escaping infection up to month $t$ has the same disease risk as a baseline seronegative vaccinee with a single breakthrough infection prior to month $t$.

We assume that the vaccine-induced neutralizing antibody titers are characterized by an initial period of fast decay, with half-life ${{hs}}_{b}$ (decay rate $\pi {1}_{b}=-{\mathrm{ln}}\left(2\right)/{{hs}}_{b}$), followed by a period of slow decay with half-life ${hl}$ (decay rate $\pi 2=-{\mathrm{ln}}\left(2\right)/{hl}$):

10 $$\begin{array}{c}{n}_{{bk}}\left(t\right)={n}_{{0}_{{bk}}}\frac{{e}^{\left({\pi 1}_{b}t+{\pi }_{2}t{s}_{b}\right)}+{e}^{\left(\pi 2t+{\pi 1}_{b}{t}{s}_{b}\right)}}{{e}^{\left({\pi 1}_{b}t{s}_{b}\right)}+{e}^{\left(\pi 2t{s}_{b}\right)}}\end{array}$$

Here, ${n}_{{0}_{{bk}}}$ is the fitted initial neutralizing antibody titer to serotype $k$, $b$ is the baseline serostatus prior to vaccination ($b\in \{-,+\}$), $t{s}_{b}$ is the time at which decay switches from fast to slow, and $t$ is the month elapsed since the second dose.

#### Likelihood

Within each trial reporting period $d$, we assume a constant serotype-specific FOI over time, ${\lambda }_{k}\left(d\right)$. Given that the model is solved at monthly intervals $t$, we set ${\lambda }_{k}\left(t\right)={\lambda }_{k}\left(d\right)$ for all months $t$ within the reporting period $d$.

For each month $t$, we calculate the probability of surviving that month without infection, given as ${e}^{-{\sum }_{k\notin \Omega }{\lambda }_{k}\left(t\right)}$ (Supplementary Fig. 1a). The probability of being seronegative at the start of month $t+1$ is therefore:

11 $${p{E}_{\varnothing }}_{{bv}}\left(j,t+1\right)={e}^{-\mathop{\sum }\limits_{k=1}^{4}{\lambda }_{k}\left(t\right)}{p{E}_{\varnothing }}_{{bv}}\left(j,t\right)$$

The probability of having been infected by the set of serotypes $\Omega$ by month $t+1$ can be represented as the sum of those who were already exposed to the serotype combination in set $\Omega$ and who escaped infection by all serotypes not in $\Omega$ during month $t$, plus those with previous exposure to all serotypes in set $\Omega$ excluding serotype $k$ (denoted ${\Omega }_{{\rm{\backslash k}}}$) who had an undetected infection to serotype $k$ during month $t-12$ (so that individuals may not be re-infected within the 12 month period of heterotypic cross-immunity, and censoring individuals with detected infections following the date of detection^20,21^):

12 $$\begin{array}{l}{{pE}}_{\Omega {bv}}\left(j,t+1\right)={e}^{-{\sum }_{k\notin \Omega }{\lambda }_{k}\left(t\right)}{{pE}}_{\Omega {bv}}\left(j,t\right)\\\qquad+\sum _{k\in \Omega }\left(1-{{{\rm{RR}}}_{{{\rm{symp}}}}}_{{cvkj}}\left(t-12\right){p}_{\rm{{{sym}{p}}}_{{ck}}}\right)\\\qquad\left(1-{{\rm{e}}}^{-{\lambda }_{k}\left(t-12\right)}\right){{pE}}_{{\Omega }_{{\rm{\backslash k}}}{bv}}\left(j,t-12\right)\end{array}$$

where $(1-{{\rm{e}}}^{-{\lambda }_{k}\left(t-12\right)}){{pE}}_{{\Omega }_{{\rm{\backslash k}}}{bv}}\left(j,t-12\right)$ is the incidence of infection with serotype $k$ during month $t-12$ in individuals previously exposed to all serotypes in set $\Omega$ excluding $k$. The probability of developing symptoms upon infection is denoted ${p}_{\rm{{{sym}{p}}}_{{ck}}}$. The second term in equation (12) represents the incidence of asymptomatic infection in the 12 months prior. Let $\gamma$ = the probability of symptoms during a secondary infection, ${\rho }_{k}$ = the serotype-specific risk of symptoms during a primary infection relative to a secondary infection, and $\varphi$ = the risk of symptomatic disease during a post-secondary infection compared with a primary infection. Thus, ${p}_{{{\rm{sym}{p}}}_{{ck}}}={\rho }_{k}\gamma \,$ for primary infections (for $c=0$), it equals *γ* for secondary infections (for $c=1$), and it equals ${\rho }_{k}\gamma \varphi$ for post-secondary infections (for $2\le c\le 3$).

The incidence of primary infection is:

13 $${{{\rm{Inc}}}}_{\Omega {bvk}}\left(j,t\right)=\left(1-{{\rm{e}}}^{-{\lambda }_{k}\left(t\right)}\right){{pE}}_{{{\varnothing }}{bv}}\left(j,t\right)$$

The incidence of secondary infection is:

14 $${{{\rm{Inc}}}}_{\Omega {bvk}}\left(j,t\right)=\left(1-{{\rm{e}}}^{-{\lambda }_{k}\left(t\right)}\right)\mathop{\sum }\limits_{k}{{pE}}_{{\Omega }_{{\rm{\backslash k}}}{bv}}\left(j,t\right)$$

where ${{pE}}_{{\Omega }_{{\rm{\backslash k}}}{bv}}\left(j,t\right)$ is the probability of having prior exposure to serotypes in set $\Omega \in {h}_{1}=\left\{\left\{1\right\},\left\{2\right\},\left\{3\right\},\left\{4\right\}\right\}$, not including serotype $k$.

The incidence of tertiary infection is:

15 $${{{\rm{Inc}}}}_{\Omega {bvk}}\left(j,t\right)=\left(1-{{\rm{e}}}^{-{\lambda }_{k}\left(t\right)}\right)\mathop{\sum }\limits_{k}{{pE}}_{{\Omega }_{{\rm{\backslash k}}}{bv}}\left(j,t\right)$$

where $$\Omega \in {h}_{2}=\left\{\left\{1,2\right\},\left\{1,3\right\},\left\{1,4\right\},\left\{2,3\right\},\left\{2,4\right\},\{3,4\}\right\}$$.

The incidence of quaternary infection is:

16 $${{{\rm{Inc}}}}_{\Omega {bvk}}\left(j,t\right)=\left(1-{{\rm{e}}}^{-{\lambda }_{k}\left(t\right)}\right)\mathop{\sum }\limits_{k}{{pE}}_{{\Omega }_{{\rm{\backslash k}}}{bv}}\left(j,t\right)$$

where $$\Omega \in {h}_{3}=\left\{\left\{1,2,3\right\},\left\{1,2,4\right\},\left\{1,3,4\right\},\{2,3,4\}\right\}$$

The incidence of disease and hospitalization, respectively, are:

17 $$\begin{array}{c}{{{{\rm{Symp}}}}_{\Omega {bvk}}\left(j,t\right)={p}_{{{\rm{sym}{p}}}_{{ck}}}{{{\rm{RR}}}_{{{\rm{symp}}}}}_{{cvkj}}\left(t\right){{\rm{Inc}}}}_{\Omega {bvk}}(j,t)\end{array}$$

18 $$\begin{array}{c}{{{{\rm{Hosp}}}}_{\Omega {bvk}}\left(j,t\right)={{p}_{{{\rm{hosp}}}}}_{{ck}}{p}_{{{\rm{sym}{p}}}_{{ck}}}{{{\rm{RR}}}_{{{\rm{hosp}}}}}_{{cvkj}}\left(t\right){{\rm{Inc}}}}_{\Omega {bvk}}\left(j,t\right)\end{array}$$

Here, ${{p}_{{{\rm{hosp}}}}}_{{ck}}$ is the probability of hospitalization. Let ${\delta }_{k}=$ the probability that a symptomatic case due to serotype $k$ is hospitalized and $\epsilon$ = the risk of hospitalization in secondary cases, compared with primary or post-secondary infections. Thus, ${{p}_{{{\rm{hosp}}}}}_{{ck}}={\delta }_{k}\epsilon$ when $c=1$, and ${\delta }_{k}$ otherwise.

Summing over the serotype combinations, the total symptomatic case and hospitalization incidence are:

19 $$\begin{array}{c}{{{\rm{Symp}}}}_{{bvk}}\left(j,t\right)=\mathop{\sum}\limits_{\Omega \in \{h_{0},{{\rm{h}}}_{1},{{\rm{h}}}_{2},{{\rm{h}}}_{3}\}}{{\rm{Symp}}}_{\Omega {bvk}}\left(j,t\right)\end{array}$$

20 $$\begin{array}{c}{{{\rm{Hosp}}}}_{{bvk}}\left(j,t\right)=\mathop{\sum}\limits_{\Omega \in \{h_{0},{{\rm{h}}}_{1},{{\rm{h}}}_{2},{{\rm{h}}}_{3}\}}{{\rm{Hosp}}}_{\Omega {bvk}}\left(j,t\right)\end{array}$$

The monthly symptomatic and hospitalization incidences are aggregated to match the months in each trial reporting period $d$, and the expected number of symptomatic and hospitalized cases are calculated as:

21 $$\begin{array}{c}{{S}_{{bvk}}\left(j,d\right)={{\rm{Symp}}}}_{{bvk}}\left(j,d\right){{N}_{{surv}}}_{v}\left(j,d\right)\end{array}$$

22 $$\begin{array}{c}{{H}_{{bvk}}\left(j,d\right)={{\rm{Hosp}}}}_{{bvk}}\left(j,d\right){{N}_{{surv}}}_{v}\left(j,d\right)\end{array}$$

where ${{N}_{{{\rm{surv}}}}}_{v}\left(j,d\right)$ is the observed population size in reporting period $d$, accounting for censoring of symptomatic cases in the previous time intervals. The observed total number of symptomatic cases and hospitalizations at time interval $d$ are assumed to follow binomial distributions:

23 $$\begin{array}{c}{N}_{{{\rm{symp}}}}\left(d\right) \sim {{\rm{Binomial}}}\left({N}_{{{\rm{surv}}}}\left(d\right),{pS}\left(d\right)\right)\end{array}$$

24 $$\begin{array}{c}{N}_{{{\rm{hosp}}}}\left(d\right) \sim {{\rm{Binomial}}}\left({N}_{{{\rm{surv}}}}\left(d\right),{pH}\left(d\right)\right)\end{array}$$

where $${pS}\left(d\right)=\sum _{b}\sum _{v}\sum _{k}\sum _{j}{S}_{{bvk}}(j,d)/{N}_{{{\rm{surv}}}}\left(d\right)$$ and $${pH}\left(d\right)=\sum _{b}\sum _{v}\sum _{k}\sum _{j}$$$${H}_{{bvk}}(j,d)/{N}_{{{\rm{surv}}}}\left(d\right)$$.

For each published time interval *d*, the expected distribution of symptomatic cases (${{pS}}_{{bvk}}\left(d\right)$) or hospitalizations (${{pH}}_{{bvk}}\left(d\right)$) in baseline serostatus $b$, due to serotype $k$, in trial arm $v$, relative to the total number of symptomatic cases (${{\rm{cum}}S}\left(d\right)=\sum _{b}\sum _{v}\sum _{k}\sum _{j}{S}_{{bvk}}(j,d)$) or hospitalizations (${{\rm{cum}}H}\left(d\right)=\sum _{b}\sum _{v}\sum _{k}\sum _{j}{H}_{{bvk}}(j,d)$), is given by:

25 $$\begin{array}{c}{{pS}}_{{bvk}}\left(d\right)=\frac{\sum _{j}{S}_{{bvk}}(j,d)}{{{\rm{cum}}S}\left(d\right)}\end{array}$$

26 $$\begin{array}{c}{{pH}}_{{bvk}}\left(d\right)=\frac{\sum _{j}{H}_{{bvk}}(j,d)}{{cumH}\left(d\right)}\end{array}$$

Equally, ${{pS}}_{{bv}}\left(j,d\right)$ and ${{pH}}_{{bv}}\left(j,d\right)$, the expected proportion of symptomatic cases and hospitalizations, respectively, in baseline serostatus $b$, trial arm $v$, age group $j$ are given by:

27 $$\begin{array}{c}{{pS}}_{{bv}}\left(j,d\right)=\frac{\sum _{k}{S}_{{bvk}}(j,d)}{{{\rm{cum}}S}\left(d\right)}\end{array}$$

28 $$\begin{array}{c}{{pH}}_{{bv}}\left(j,d\right)=\frac{\sum _{k}{{{\rm{Hosp}}}}_{{bvk}}(j,d)}{{{\rm{cum}}H}\left(d\right)}\end{array}$$

Finally, ${{pS}}_{k}\left(j,d\right)$ and ${{pH}}_{k}\left(j,d\right)$, the expected proportion of symptomatic cases or hospitalizations, respectively, due to serotype $k$ in age group $j$ are given by:

29 $$\begin{array}{c}{{pS}}_{k}\left(j,d\right)=\frac{\sum _{b}\sum _{v}{S}_{{bvk}}(j,d)}{{{\rm{cum}}S}\left(d\right)}\end{array}$$

30 $$\begin{array}{c}{{pH}}_{k}\left(j,d\right)=\frac{\sum _{b}\sum _{v}{{{\rm{Hosp}}}}_{{bvk}}(j,d)}{{{\rm{cum}}H}\left(d\right)}\end{array}$$

The expected probabilities of observing symptomatic disease and hospitalized cases given in equations (25)–(30) are assumed to follow multinomial distributions:

31 $$\begin{array}{c}{{N}_{{{{{\rm{symp}}}}}}}_{{bvk}}\left(d\right) \sim {{{{\rm{Multinomial}}}}}\left({{pS}}_{{bvk}}\left(d\right)\right)\end{array}$$

32 $$\begin{array}{c}{{N}_{{{\rm{symp}}}}}_{{bv}}\left(j,d\right) \sim {{\rm{Multinomial}}}\left({{pS}}_{{bv}}\left(j,d\right)\right)\end{array}$$

33 $$\begin{array}{c}{{N}_{{{\rm{symp}}}}}_{k}\left(j,d\right) \sim {{\rm{Multinomial}}}\left({{pS}}_{k}\left(j,d\right)\right)\end{array}$$

34 $$\begin{array}{c}{{N}_{{{\rm{Hosp}}}}}_{{bvk}}\left(d\right) \sim {{\rm{Multinomial}}}\left({{pH}}_{{bvk}}\left(d\right)\right)\end{array}$$

35 $$\begin{array}{c}{{N}_{{{\rm{Hosp}}}}}_{{bv}}\left(j,d\right) \sim {{\rm{Multinomial}}}\left({{pH}}_{{bv}}\left(j,d\right)\right)\end{array}$$

36 $$\begin{array}{c}{{N}_{{{\rm{Hosp}}}}}_{k}\left(j,d\right) \sim {{\rm{Multinomial}}}\left({{pH}}_{k}\left(j,d\right)\right)\end{array}$$

#### Model variants and sensitivity analyses

To explore whether the data supported varying parameters by baseline serostatus, serotype, outcome and age group, we considered multiple simpler nested models than those outlined above. Model variants were compared visually and quantitatively using the log-likelihood and the expected log-predictive density, estimated using the Watanabe–Akaike information criterion, to find the most parsimonious, biologically motivated model that could reproduce the trial data.

We ran a sensitivity analysis on the choice of the prior normal distribution of the enhancement parameter ${L}_{{ck}}$ centered and truncated at 0, with standard deviations {0.25, 0.5, 0.75, 1.00, 1.25, 1.50, 1.75, 2.00}. We assessed the impact of the choice of the prior distribution on the log-likelihood, posterior estimate of ${L}_{{ck}}$, and the Bayes factor estimate comparing models with and without vaccine-associated enhancement (that is, setting ${L}_{{ck}}=0$).

We also explored the sensitivity of the VE estimates to assumptions made about: the period of heterotypic cross-immunity, which we fixed at 1 and 6 months (rather than 12 months as in our main analysis); whether multitypic individuals could be misclassified as seronegative at baseline; whether all post-secondary infections are asymptomatic; whether the titer required for 50% protection against symptomatic dengue (${n}_{50}$) in seronegative and multitypic individuals should be serotype specific; and whether the risk ratio of vaccine-associated hospitalization enhancement compared with symptomatic disease ($\tau$) should be serotype specific.

#### Simulation study to assess the model identifiability

To confirm the identifiability of the model parameters given the data, we conducted a simulation study. We sampled 20 sets of the parameters to generate simulated datasets from distributions that were wider or centered away from the prior distributions used for parameter inference. The only exception was the parameters describing the titer decay (${hs}$, ${hl}$ and ${ts}$), which were estimated directly from the observed data and were intentionally kept informative. We then simulated case data using each set of parameters. The case data were comparable to the observed data in magnitude and distribution across time, age group, serostatus and trial arm. Finally, we calibrated the survival model to the simulated case data and compared the simulated posterior distribution of each parameter to the true parameter distributions.

#### Inferential framework

The model was fitted to the baseline seropositivity data (${N}_{{{\rm{SP}}}}$) and number of VCD symptomatic (${N}_{{{\rm{symp}}}}$) and hospitalized (${N}_{{{\rm{hosp}}}}$) cases observed across the trial, stratified by baseline serostatus, age and serotype within a Bayesian framework. Parameter inference was carried out using the Hamiltonian Monte Carlo algorithm and the No-U-Turn sampler via Stan, a probabilistic programming language implemented via the cmdstanr package^50^ (version 0.8.1) in R^51^ (version 4.2.2) and RStudio (version 2023.06.1+524). Four chains were run for 10,000 iterations each, and we discarded the first 5,000 iterations as burn-in. Convergence was assessed visually and using the $\hat{R}$ statistic^52^. The estimated parameters, priors and prior sources are listed in Supplementary Table 3.

### Transmission model

To evaluate the population-level impact of Qdenga, we applied the VE estimates obtained above to a 4-serotype stochastic compartmental model of dengue transmission, a deterministic version of which was published by Ferguson et al.^4^ (Supplementary Fig. 1b). The model includes mosquito population dynamics with a seasonally varying carrying capacity. It models primary to quaternary human infections, assuming that homotypic immunity is lifelong, and heterotypic immunity is temporary (12 months). It tracks the serotype-specific infection history but not the order of past infections. The model is stratified by vaccine status, serostatus and age, with single age classes for the first 20 years and 10 years thereafter. The model incorporates realistic non-stationary human demography, calibrated to the demographic estimates for Brazil and the Philippines published in the UN World Population Prospects 2022 (ref. ^53^), which show that the Brazilian population is older than the Philippine’s population (Supplementary Fig. 29). For this study, we embedded the VE model detailed above into this transmission model. Full details of the transmission model are given below.

#### Mosquito dynamics

The transmission model accounts for mosquito population dynamics following the Ross–MacDonald model, with varying seasonal carrying capacity. Specifically, adult female mosquitoes lay eggs at rate $\Gamma$ and larvae ($L$) mature into adult mosquitos ($M$) at rate ${\rm{{\rm E}}}$. Larvae are regulated by a power density-dependent mortality rate $\mu$, with a seasonal carrying capacity $K$, ensuring that mortality increases as the larval population increases. Adult mosquitoes are born susceptible (${M}_{S}$), experience a serotype-specific FOI ${\Lambda }_{k}$, and infected mosquitoes enter an exposed but not infectious compartment (${M}_{E}$) that lasts on average for a period $\frac{1}{{\boldsymbol{\xi }}}$ days, the extrinsic incubation period. Finally, the adult mosquitoes that survive the extrinsic incubation period become infectious to humans (${M}_{I}$) until their death. Adult mosquitoes die at a rate $\Delta$. The ordinary differential equations (ODEs) governing the mosquito dynamics are given as follows:

37 $$\begin{array}{l}\begin{array}{l}\begin{array}{llc}\displaystyle\frac{{dL}}{{dt}}\,=\,\Gamma M-\left({{{\rm E}}}+\mu \right)L\\ \displaystyle\frac{d{M}_{S}}{{dt}}\,=\,{{{\rm E}}}L-\left(\Delta +\mathop{\sum}\limits_{k}{\Lambda }_{k}\right){M}_{S}\end{array}\\ \begin{array}{llc}\displaystyle\frac{d{M}_{{E}_{k}}}{{dt}}\,=\,{\Lambda }_{k}{M}_{S}-\left(\xi +\Delta \right){M}_{{E}_{k}}\\ \displaystyle\frac{d{M}_{{I}_{k}}}{{dt}}\,=\,\xi {M}_{{E}_{k}}-\Delta {{{\rm{M}}}_{{\rm{I}}}}_{k}\end{array}\end{array}\end{array}$$

The mortality rate $\mu$ of the larvae is defined as:

38 $$\begin{array}{c}\mu =\sigma \left(1+\frac{L}{{KN}}\right)\end{array}$$

where $\sigma$ is the low-density limit of larval death and $N$ is the total human population.

In turn, the seasonal carrying capacity $K$ is defined as:

39 $$\begin{array}{c}K=\bar{K}\left(1+{K}_{s}\cos \left(2\pi t* 365\right)\right)\end{array}$$

Where $\bar{K}$ is the mean carrying capacity, ${K}_{s}$ is the magnitude of the seasonal variation in carrying capacity, and $t$ is time in days. We ran model simulations across nine transmission intensities, characterized by SP9 (see the model simulation section below for more details). For each value of SP9 between 10% and 90% in 10% steps, we determined the mean carrying capacity that led to that value of SP9 across the period 2020–24. The resulting carrying capacity values gave adult female mosquito densities per person ($A$) ranging from 1 to 5, compatible with values reported by Focks et al., who estimated a maximum of 1.5 *Aedes aegypti* pupae per person for ambient air temperatures of 28 °C (ref. ^54^):

40 $$\begin{array}{c}\bar{K}={\rm{{\rm A}}}\Delta \frac{{\left({\rm{{\rm E}}}\frac{\Gamma -\Delta }{\Delta \sigma }-1\right)}^{-\frac{1}{\xi }}}{{\rm{{\rm E}}}}\end{array}$$

Values and data sources for mosquito parameters are provided in Supplementary Table 4.

The rate at which adult female mosquitoes lay eggs $\Gamma$ is assigned to give the required mosquito population reproduction number (that is, representing the reproductive capacity of the mosquito population, rather than anything to do with disease transmission):

41 $$\begin{array}{c}{{R}_{0}}_{m}=\Delta \frac{{\rm{{\rm E}}}}{{\rm{{\rm E}}}+{\rm{\mu }}}\Gamma \end{array}$$

The probability of transmission from human to mosquito ${{\rm{{\rm B}}}}_{{hm}}$ is defined to give the required dengue ${R}_{0}$ as follows:

42 $${{\rm{{B}}}}_{{hm}}=\frac{{R}_{0}}{{\kappa }^{2}{\rm{{A}}}\eta \frac{\frac{\overline{{{\rm{{B}}}}_{{mh}}}}{1+\Delta \xi }}{\Delta }}$$

Where $\eta$ is the human infectious period and $\kappa$ is the biting rate per mosquito.

Finally, the FOI experienced by mosquitoes due to dengue serotype $k$, ${\Lambda }_{k}$, is:

43 $$\begin{array}{c}{\Lambda }_{k}={{\rm{{\rm B}}}}_{{hm}}\kappa \frac{c{I}_{k}}{N}\end{array}$$

Where $c{I}_{k}$ is the human incidence of infection (defined below in equation (50)) with serotype $k$, and $N$ is the total size of the human population.

#### Human transmission dynamics

As with the cohort survival model, let subscript $\Omega$ denote the set of serotypes to which an individual has previously been exposed (with cardinality $c$). In the transmission model, we assume that individuals are born susceptible to all four serotypes ${S}_{\varnothing }$, after which they are subject to time-varying serotype-specific FOI ${\lambda }_{k}(t)$. Infection with any of the four serotypes confers long-lasting homotypic immunity and heterotypic immunity against the other serotypes for a period $1/{\rm{\vartheta }}$ (12 months) (${R}_{\Omega }$). After this, individuals are assumed to be susceptible to infection with heterotypic serotypes. In total, an individual may be infected up to four times, and we track an individual’s infection history but not the serotype-specific order of infection. The model is solved daily ($t$).

The model is additionally stratified by the age group *i* (1–20 years of single age classes, then 10 year age classes up to 80 years), and vaccination status $v$ (1, unvaccinated; 2, vaccinated), with individuals aged $f$ vaccinated with coverage ${\rm{\upsilon }}$. Aging is modeled as an annual discrete event (for example, all 1-year-olds move to the 2-year-old age group) to track the vaccinated cohort accurately up to the age of 20. We assume that vaccination also occurs once per year at the same time as the ageing process and we do not separately model the receiving of the first and second dose. The birth rate $\Psi ({\rm{t}})$ and the age-specific human mortality rate ${{\rm{\theta }}}_{{\rm{i}}}(t)$ are both calibrated to match the demography of either the Philippines or Brazil (see the model simulation section below for additional details). The ODEs governing human transmission dynamics are:

44 $$\begin{array}{l}\frac{d{S}_{\Omega {vi}}}{{dt}}={{\rm{\delta }}}_{\Omega ,{{\varnothing }}}{{\rm{\delta }}}_{{\rm{v}},1}{{\rm{\delta }}}_{{\rm{i}},0}\Psi \left(t\right){S}_{\Omega {vi}}+{{\rm{\delta }}}_{{\rm{i}},\;f}{{\upsilon }}\left[{{{\rm{\delta }}}_{{\rm{v}},2}S}_{\Omega {vi}}-{{{\rm{\delta }}}_{{\rm{v}},1}S}_{\Omega {vi}}\right]\\\qquad\quad-\left(\mathop{\sum}\limits_{k\notin \Omega }{{\rm{\lambda }}}_{{\rm{k}}}\left(t\right){{\rm{RR}}}_{{\inf}_{{cvki}}}(t)+{{\rm{\theta }}}_{{\rm{i}}}(t)\right){S}_{\Omega {vi}}\\ \frac{d{R}_{\Omega {vi}}}{{dt}}={{\rm{\delta }}}_{{\rm{i}},\;f}{\rm{\upsilon }}\left[{{{\rm{\delta }}}_{{\rm{v}},2}R}_{\Omega {vi}}-{{{\rm{\delta }}}_{{\rm{v}},1}R}_{\Omega {vi}}\right]\\\qquad\quad+\left(\mathop{\sum}\limits_{k\notin \Omega }{{\rm{\lambda }}}_{{\rm{k}}}\left(t\right){{\rm{RR}}}_{{\inf}_{cvki}}\left(t\right)\right){S}_{\Omega {vi}}-\left({\rm{\vartheta }}+{{\rm{\theta }}}_{{\rm{i}}}(t)\right){R}_{\Omega {vi}}\end{array}$$

Here ${{\rm{\delta }}}_{{\rm{x}},{\rm{y}}}$ is the Kronecker delta function (equal to 1 when ${\rm{x}}={\rm{y}}$ and 0 otherwise), the first term of $\frac{d{S}_{\Omega {vi}}}{{dt}}$ represents births, the second term represents vaccination, and the final term represents infections and deaths. The parameter ${\rm{RR}}_{{\inf}_{cvki}}\left(t\right)$ is the vaccine-associated RR of infection (see equation (51) below for details). The first term of $\frac{d{R}_{\Omega {vi}}}{{dt}}$ represents vaccination, the second term represents infection, and the third term represents waning heterotypic immunity and mortality. All transitions between compartments (ageing, infection, waning immunity, death and vaccination) are drawn from binomial distributions to introduce model stochasticity.

The FOI on humans due to serotype $k$ is:

45 $${{\rm{\lambda }}}_{{\rm{k}}}\left(t\right)={{\rm{{B}}}}_{{mh}}\kappa \frac{{M}_{{I}_{k}}}{N}$$

where ${{\rm{{\rm B}}}}_{{mh}}$ is the per bite transmission probability from mosquitoes to humans.

The incidence of individuals exposed (but not yet infectious) to serotype $k$ is given as:

46 $$\begin{array}{c}{E}_{{\rm{c}}{vki}}\left(t\right)={{\rm{\lambda }}}_{{\rm{k}}}\left(t\right){{\rm{RR}}}_{{\inf}_{{cvki}}}\left(t\right){S}_{\Omega _{\rm{\backslash k}}vi}\end{array}$$

The incidence of individuals who are infectious $I$, symptomatic $D$, or hospitalized $H$ due to serotype $k$ at time $t$ is given as:

47 $$\begin{array}{c}{I}_{{\rm{c}}{vki}}\left(t\right)=\zeta {E}_{{\rm{c}}{vki}}\end{array}$$

48 $$\begin{array}{c}{D}_{{\rm{c}}{vki}}\left(t\right)={{{\rm{RR}}}_{{{\rm{symp}}}}}_{{\rm{c}}{vki}}\left(t\right){p}_{{{\rm{sym}{p}}}_{c}}{I}_{{\rm{c}}{vki}}\left(t\right)\end{array}$$

49 $$\begin{array}{c}{H}_{{\rm{c}}{vki}}\left(t\right)={{{\rm{RR}}}_{{{\rm{hosp}}}}}_{{\rm{c}}{vki}}\left(t\right)Q{p}_{{{\rm{sym}{p}}}_{c}}{I}_{{\rm{c}}{vki}}\left(t\right)\end{array}$$

Here $1/\zeta$ is the human incubation period, ${p}_{{sym}{p}_{c}}$ is the probability that an infected individual with serostatus $c$ is symptomatic (= $\rho \gamma$ for primary infections, $\gamma$ for secondary infections and $\rho \gamma \varphi$ for post-secondary infections, see equation (12) above for parameter descriptions), $Q$ is the probability that a symptomatic individual requires hospitalization (fixed at 9% to match the average probability of hospitalization observed in Brazil and the Philippines during the phase III clinical trial), and ${{{\rm{RR}}}_{{symp}}}_{{cvki}}\left(t\right)$ and ${{{\rm{RR}}}_{{{\rm{hosp}}}}}_{{cvki}}\left(t\right)$ are the vaccine-associated RR of disease and hospitalization (defined in equations (8) and (9) above).

The cumulative incidence of individuals infectious to serotype $k$, irrespective of serostatus, vaccine status or age group, as seen in equation (43) above is therefore:

50 $$c{I}_{k}=\mathop{\sum}\limits_{c}\mathop{\sum}\limits_{v}\mathop{\sum}\limits_{i}{I}_{{cvki}}$$

#### Model parameter values

The VE parameters (${hs}$, ${hl}$, ${ts}$, $L$, ${n}_{50}$, $\alpha$, $\beta$, $w$ and $\tau$) and probabilities of symptoms ($\rho$, $\gamma$, $\varphi$) were sampled from the posterior distribution of the survival model used to reconstruct ${\rm{RR}}_{\rm{symp}}$ and ${{{\rm{RR}}}}_{{{\rm{hosp}}}}$ as described in equations (8) and (9). All other parameter values used in the stochastic compartmental model of transmission are listed in Supplementary Table 4.

#### Model simulations

We equilibrated the transmission dynamics by running the transmission model in the absence of vaccination for 175 years, starting from 1850. For the first 100 years we assumed a static 1950s demography. From 1950 onwards we assumed time-varying demographies matching the UN World Population Prospects 2022 estimates for Brazil or the Philippines^53^, to explore the sensitivity of the impact estimates to different population structures (Supplementary Figs. 29 and 30). We assumed that vaccination started in 2024.

We ran multiple scenarios to investigate the vaccines impact across: two demographies (Brazil and the Philippines); nine transmission intensity settings, defined using SP9, from 10% to 90% in steps of 10%; four vaccine coverages (20%, 40%, 60%, 80%); seven ages at vaccination (from 6 to 12 years of age); four hypotheses about the vaccine’s mechanism of action, obtained by assuming vaccine waning for either 5 years (approximately the period of the phase III trial) or 15 years post-vaccination (extrapolating beyond the trial), and that the vaccine protects against clinical disease only (VS), or also against infection (VI, see equation (51) below); and finally, with and without pre-vaccination screening of individuals’ serostatus assuming a diagnostic test with 94.7% specificity and 89.6% sensitivity^29^.

We expressed transmission intensity in terms of SP9 because the latter can be directly measured from seroprevalence surveys. Conversely, the reproduction number (R_0_) depends on additional assumptions (for example, the extent to which post-primary infections contribute to transmission, as described in the Imai et al. study^55^) and is therefore difficult to estimate empirically for dengue. However, we note that specifying one of R_0_, FOI and SP9 determines the values of the other two variables, as shown in Supplementary Fig. 30. Supplementary Fig. 31 shows model assumptions about the seasonality of dengue transmission intensity. We assumed that all serotypes have near-identical transmissibility but relaxed this assumption in a sensitivity analysis.

In the absence of serological tests able to identify the infecting serotype and infer asymptomatic infections, data on Qdenga’s efficacy against infection are limited, but there is evidence that it may offer limited protection against infection for some months^28^. In the VI modeling scenarios we therefore assumed that the probability of infection, as for clinical outcome, can be explained by the antibody titer induced by previous infection^8^ or vaccination^13^. We assumed that the titers required for protection from infection are higher than those required to protect against symptomatic disease, and that (unlike enhancement of disease) there is no enhancement of infection risk^8,13^. We therefore modeled a scenario of low–moderate VE against infection by scaling the posterior estimates for ${n}_{{50}_{{ck}3}}$(the neutralizing titer conferring 50% protection against disease in the absence of infection) in equation (8) by ${{\alpha }_{\inf }}_{c}$, equal to 12 if $c=0$ (primary infection) and 3 if $1\le c\le 3$:

51 $${{{\rm{RR}}}_{\inf }}_{{cvkj}}\left(t\right)=\frac{1}{1+{\left(\frac{{n}_{{ck}}\left(t\right)}{{{{{\alpha }_{\inf }}_{c}e}^{{{\beta }_{{{\rm{symp}}}}}_{j}}n}_{{50}_{{ck}3}}}\right)}^{{w}_{{ck}}}}\,\,\,{{\rm{if}}\,v}=2$$

The resulting ${{\rm{VE}}}_{\inf }$ curves are plotted in Supplementary Fig. 14.

For each of the modeling scenarios, we sampled the VE parameters from the posterior distributions 200 times (Supplementary Table 1). Then, for each posterior parameter set we ran the stochastic transmission model 50 times (where the probability of infection and vaccination are drawn from binomial distributions), giving 10,000 simulations in total for each scenario (Supplementary Fig. 1b).

Each model simulation was run twice: once with VE set to our estimated values (‘vaccination’ scenario) and once with VE set to zero (‘no vaccination’ scenario). This enabled us to estimate the population- and individual-level impact of vaccination. Individual impact is measured as the proportion of cases averted in the first vaccinated cohort (over a period of 10 years) and population-level impact is the proportion of cases averted in the entire population, including non-vaccinated individuals (over a period of 10 years) (Supplementary Fig. 1b):

52 $$\begin{array}{l}{\rm{prop}}_{\rm{av}}=\displaystyle \frac{\left({\rm{cases}}_{\rm{NV}}-{\rm{cases}}_{\rm{V}}\right)}{{\rm{cases}}_{\rm{NV}}}\end{array}$$

Here ${\rm{cases}}_{\rm{NV}}$ is the number of cases in the zero VE (‘no vaccination’) counterfactual scenario, and ${\rm{cases}}_{\rm{V}}$ is the number of cases in the VE > 0 (‘vaccination’) scenario.

We summarize uncertainty in two ways. The first is the overall uncertainty, calculated over the 10,000 realizations, which represents uncertainty in both the posterior parameter estimates and the stochastic simulations. The second is the parameter uncertainty, calculated as the uncertainty of the mean of the 50 stochastic simulations for each of the 200 posterior samples.

We note that the vaccination and counterfactual (that is, no vaccination) runs were matched exactly (that is, identical transmission dynamics were simulated for each such pair of simulations) up to the point of vaccine introduction. In the VS scenario (no VE against infection), transmission dynamics remained identical for the vaccination and no-vaccination scenarios following the introduction, but this was not the case if non-zero VE against infection was assumed (given that vaccination then modified transmission).

#### Transmission model sensitivity analyses

We explored the sensitivity of the population impact estimates to, first, the period of heterotypic cross-immunity, which we assumed to be 6 months (rather than 12 months as in our main analysis); second, the VE estimates obtained when assuming serotype-specific titers for 50% protection against symptomatic dengue in seronegative and multitypic individuals (rather than single seronegative and multitypic estimates used in our main analysis); third, the VE estimates obtained when we estimate a single risk ratio of vaccine-associated hospitalization enhancement compared with symptomatic disease (rather than serotype specific as in our main analysis); fourth, the lack of seasonality in mosquito carrying capacity in the transmission model; and last, the R_0_ differences between serotypes, with relative values of 1.05, 1.15, 0.95 and 0.85 for DENV1–4, respectively.

### Reporting summary

Further information on research design is available in the Nature Portfolio Reporting Summary linked to this article.

## Online content

Any methods, additional references, Nature Portfolio reporting summaries, source data, extended data, supplementary information, acknowledgements, peer review information; details of author contributions and competing interests; and statements of data and code availability are available at 10.1038/s41591-025-03771-y.

## Supplementary information

Supplementary Information Supplementary methods, Tables 1–4 and Figs. 1–31.

Reporting Summary

## Data Availability

All data used in the modeling study are available on GitHub at https://github.com/mrc-ide/qdenga_impact.

## Code availability

Code is available at https://github.com/mrc-ide/qdenga_impact.
