# Supplementary material for: Efficacy, public health impact and optimal use of the Takeda dengue vaccine
Source: Nat Med. 2025 Jun 25;31(8):2663–72. doi: 10.1038/s41591-025-03771-y (PMC12353809; doi:10.1038/s41591-025-03771-y)
Supplement: Supplementary file 1 — Supplementary methods, Tables 1–4 and Figs. 1–31. [file 41591_2025_3771_MOESM1_ESM.pdf]

nature medicine

Supplementary information https://doi.org/10.1038/s41591-025-03771-y

Efficacy, public health impact and optimal use of the Takeda dengue vaccine

In the format provided by the authors and unedited

Supplementary Information
Efficacy, public health impact and optimal use of the Takeda dengue vaccine

Bethan Cracknell Daniels[1], Neil M. Ferguson[1]*, Ilaria Dorigatti[1]*

1. MRC Centre for Global Infectious Disease Analysis and the Abdul Latif Jameel Institute for Disease and Emergency Analytics, School of Public Health, Imperial College London, London, UK

*Corresponding authors Neil Ferguson (neil.ferguson@imperial.ac.uk) and Ilaria Dorigatti (i.dorigatti@imperial.ac.uk)

Table of Contents

Supplementary tables ***2***

Supplementary figures ***6***

Supplementary tables

Supplementary Table 1: Mean and 95% CrI posterior parameter estimates of the survival model (M30), used to estimate vaccine efficacy.

Parameter			Estimate (mean and 95% CrI)
hs_c	Seronegative		1.93 (1.12 to 2.72)
	Seropositive		4.34 (3.58 to 5.11)
hl	-		72.14 (49.38 to 94.25)
ts_c	Seronegative		-2.10 (-2.93 to -1.28)
	Seropositive		0.31 (-0.52 to 1.13)
p_j	4-5yrs		0.23 (0.21 to 0.24)
	6-11yrs		0.30 (0.28 to 0.31)
	12-16yrs		0.47 (0.44 to 0.50)
γ	-		0.46 (0.34 to 0.63)
$1/\rho$	-		2.44 (2.13 to 2.79)
φ	-		0.26 (0.18 to 0.34)
δ_k	DENV1		0.19 (0.15 to 0.23)
	DENV2		0.40 (0.34 to 0.47)
	DENV3		0.17 (0.12 to 0.21)
	DENV4		0.14 (0.06 to 0.24)
L	-		0.84 (0.13 to 1.84)
τ_k	DENV1		1.31 (0.22 to 2.57)
	DENV2		1.26 (0.14 to 2.70)
	DENV3		2.18 (0.81 to 3.55)
	DENV4		1.14 (0.12 to 2.55)
w	-		2.73 (1.80 to 3.89)
$log(n_{50_{ck}})$	Seronegative		4.86 (4.46 to 5.28)
	Monotypic	DENV1	6.74 (6.54 to 6.92)
	Monotypic	DENV2	6.98 (6.58 to 7.28)
	Monotypic	DENV3	6.82 (6.61 to 7.04)
	Monotypic	DENV4	6.04 (5.48 to 6.58)
	Multitypic		6.51 (4.91 to 7.81)
α	-		0.49 (0.30 to 0.74)
β_j	4-5yrs		0.49 (0.29 to 0.76)
$sens$	-		0.91 (0.89 to 0.93)
$spec$	-		0.99 (0.98 to 1.00)
$\lambda_k\ (d\ )$	DENV1 to DENV4	1-12, 13-18, 19-24, 25-36, 37-48, and 49-54 months	Range: 6.32^{-05} (5.22^{-06} to 1.93^{-04}) - 1.15^{-02} (7.16^{-03} to 1.69^{-02})

Supplementary Table 2: Neutralising antibody titres providing 50% vaccine efficacy against symptomatic disease or hospitalisation in seronegative individuals.

Outcome	Age group	Serotype	Neutralising antibody titre
Symptomatic	4-5yrs	All	293
	6-16yrs		182
Hospitalisation	4-5yrs	DENV1	193
		DENV2	194
		DENV3	224
		DENV4	189
	6-16yrs	DENV1	122
		DENV2	122
		DENV3	140
		DENV4	119

Supplementary Table 3: Survival model parameters and their priors.

Parameter	Description	Prior distribution	Source
hs_c	Short half-life of antibody decay in seronegatives (c= 0)	Normal (1.65,0.5)	[1]
hs_c	Short half-life of antibody decay in seropositives ($c = 1 = 2 = 3$)	Normal (4.20,0.50)	[1]
hl	Long half-life of antibody decay	Normal (84.00,12.00)	[2]
ts_c	Time period switch decay in seronegatives ($c = 0$)	Normal (-2.21, 0.50)	[1]
ts_c	Time period switch decay in seropositives ($c = 1 = 2 = 3$)	Normal (0.15, 0.50)	[1]
L_{ck}	Enhancement of symptomatic disease in vaccinated seronegative individuals ($c = 0$)	Normal (0.00,1.00)	
L_{ck}	Enhancement of symptomatic disease in vaccinated seropositive individuals ($c = 1 = 2 = 3$)	Fixed at 0.00	
w_{ck}	Shape parameter	Normal (1.00,2.00)	[3]
$log(n_{50_{ckj}})$	Log neutralising antibody titre against each serotype which provides 50% protection against symptomatic disease (in the absence of enhancement) for seronegative individuals in age group 3 ($c = 0, j = 3$)	Normal (4.50,1.00)	[1,4–7]
$log(n_{50_{ckj}})$	Log neutralising antibody titre against each serotype which provides 50% protection against symptomatic disease (in the absence of enhancement) for seropositive individuals in age group 3 ($c = 1 = 2 = 3, j = 3$)	Normal (6.50,1.00)	[1,4–7]
τ_k	Risk ratio of enhancement of hospitalisation compared to enhancement of symptomatic disease	Normal (1.00,1.00)	
α_{ck}	Difference in neutralising antibody titre required for 50% protection from hospitalisation compared to symptomatic disease	Normal (0.00,2.00)	
β_{symp_j}	Difference in neutralising antibody titre required for 50% protection from symptomatic disease in age groups 1 and 2 ($j = 1, 2$), compared to age group 3	Normal (0.00,2.00)	

β_{hosp_j}	Difference in neutralising antibody titre required for 50% protection from hospitalisation in age groups 1 and 2 ($j = 1,2$), compared to age group 3	Normal (0.00,2.00)	
p_j	Probability of exposure in age groups 1 and 2 ($j = 1,2$)	Beta (3.00,5.00)	
p_{kj}	Probability of exposure to serotype k in age group 3 ($j = 3$)	Beta ($shape1, shape2$)	
$\lambda_k(d)$	Serotype-specific force of infection during the trial	Lognormal (-7.00,2.00)	
γ	Probability a secondary infection is symptomatic	Normal (0.85,0.20)	[8]
$1/\rho_k$	Risk ratio of disease in primary infections compared to secondary infections	Normal (1.97,0.40)	[8]
φ	Probability that a post-secondary infection is symptomatic compared to a primary infection	Normal (0.25,0.05)	[8]
δ_k	Probability a symptomatic case is hospitalised	Normal (0.25,0.10)	[9]
ϵ	Risk ratio of hospitalisation in secondary cases compared to primary or post-secondary cases	Normal (1.00,1.00)	
$sens$	Sensitivity of the microneutralisation test used to classify serostatus at baseline	Normal (0.90,0.05)	[10]
$spec$	Specificity of the microneutralisation test used to classify serostatus at baseline	Normal (0.995,0.01)	[10]

Supplementary Table 4: Transmission model parameters.

Parameter	Description	Value	Source
Γ	Rate at which adult females produce female larvae	Calculated to give the required R_{0_m}	
$\bar{K}$	Mean larval mosquito carrying capacity	Calculated to match the required adult wild-type female mosquito density per person	
μ	Larval mosquito mortality rate	Derived from the larvae dependence in eq. 38	
ξ	Extrinsic incubation period	8 days	[8]
Δ	Adult mosquito death rate	0.1/day	[11]
E	Rate larvae develop into adult mosquitoes	Assigned 1/19 days, to match R_{0_m}	[8]
σ	Low-density limit of larval death	0.025/day	[12]
K_s	Magnitude of the seasonal variation in carrying capacity	0.3	[8]
A	Adult wild-type female mosquito density per person	Varied in range 1 to 5 to reproduce required mean dengue transmission intensity (dengue reproduction number/force of infection)	[13]
R_{0_m}	Mosquito population reproduction number	2.69 based on the estimate of female fecundity of 0.269/day and adult mortality rate of 0.1/day	[14]
B_{mh}	Probability of transmission from mosquito to human	1.0	[8]
B_{hm}	Probability of transmission from human to mosquito	Calculated to give the required reproduction number in eq. 42 of the main text	

R_0	Reproduction number of dengue	Varied from ~1.1 to ~5 to match required equilibrium pre-vaccination seropositivity in 9-year-olds of 10-90%.	
η	Human infectious period	4 days	[15–18]
κ	Biting rate per mosquito per day	0.6/day	[8,19]
ϑ	Period of heterotypic immunity	12 months	[20,21]
$\theta_i(t)$	Human mortality rate	Fitted to either Brazilian or Philippines demography from the UN World Population Prospects	[22]
$\Psi(t)$	Human birth rate	Scaled estimates from the UN World Population Prospects corresponding to Brazilian or Philippines to give a population of 5 million in 1950	[22]
ζ	Intrinsic incubation period	5 days	[8]
υ	Vaccination coverage	Varied: 20%, 40%, 60%, 80%	
Q	Proportion of symptomatic infections which require hospitalisation in Brazil or the Philippines	Set as 0.09 to match the average probability of hospitalisation observed in Brazil and the Philippines during the phase III clinical trial	[4]

Supplementary figures

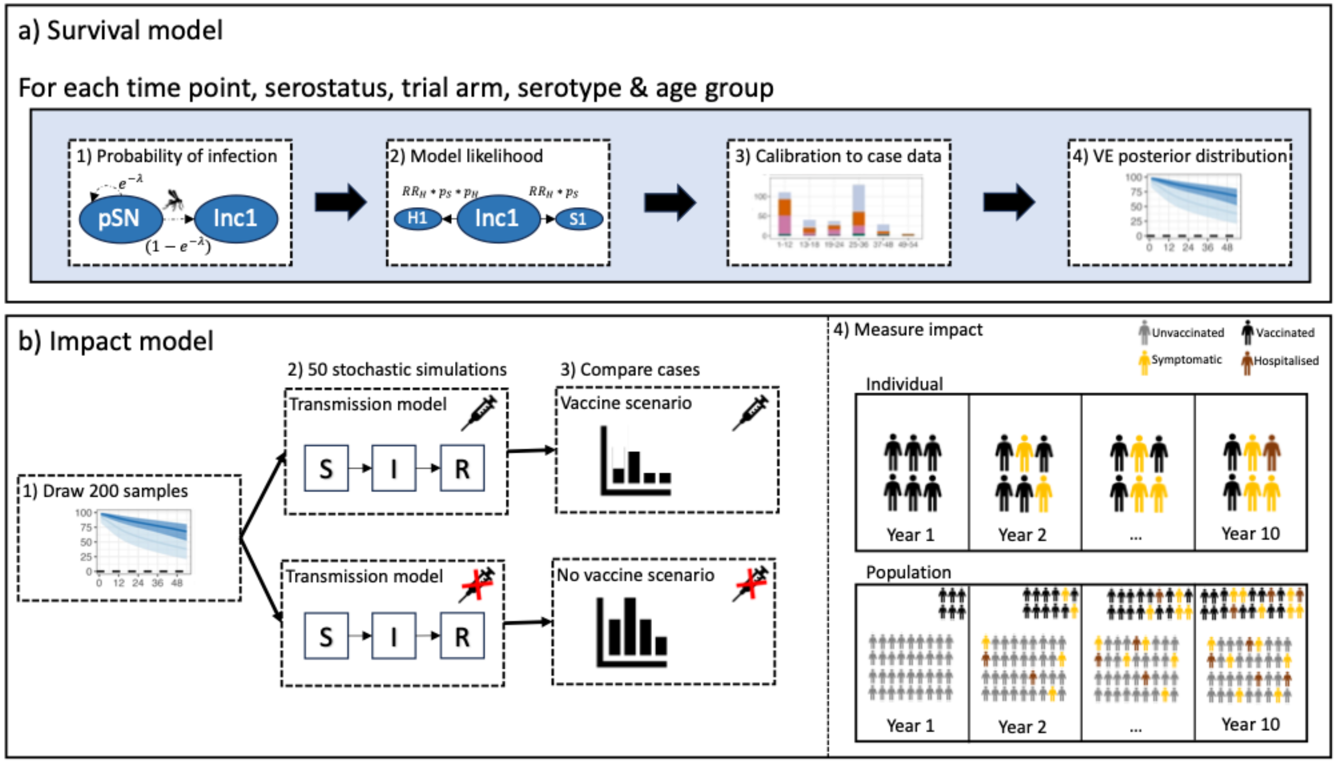

Supplementary Figure 1: Conceptual figure of the modelling approach using in the study. (a) Overview of the Bayesian cohort survival model used to reproduce the phase III clinical trial case data and estimate vaccine efficacy, using a seronegative individual as an example and outlining how the model keeps track of (1) the probability that seronegative individuals (pSN) are infected and (2) the incidences of primary symptomatic disease (S1) and hospitalisation (H1) are reconstructed from the incidence of primary infection (Inc1) through the vaccine-associated risk ratios of symptomatic disease and hospitalisation RR_S and RR_H , respectively and (3) the calibration of the survival model to the published case data, which allows (4) the estimation of the vaccine efficacy (VE) over-time. **(b)** Overview of the modelling framework used to estimate the impact of routine vaccination with the compartmental model. For each scenario of routine vaccination, we drew 200 posterior samples of the VE parameters and for each sample, we (2) run 50 simulations of the compartment transmission model with (top) and without (bottom) vaccination, to output (3) the expected number of cases and thus (4) assess the impact of vaccination in two ways: at the individual-level (top) impact is calculated as the proportion of cases averted in the first vaccinated cohort over ten years; at the population-level (bottom) impact is calculated as the proportion of cases averted in the entire population (including the non-vaccinated population) over ten years of routine vaccination.

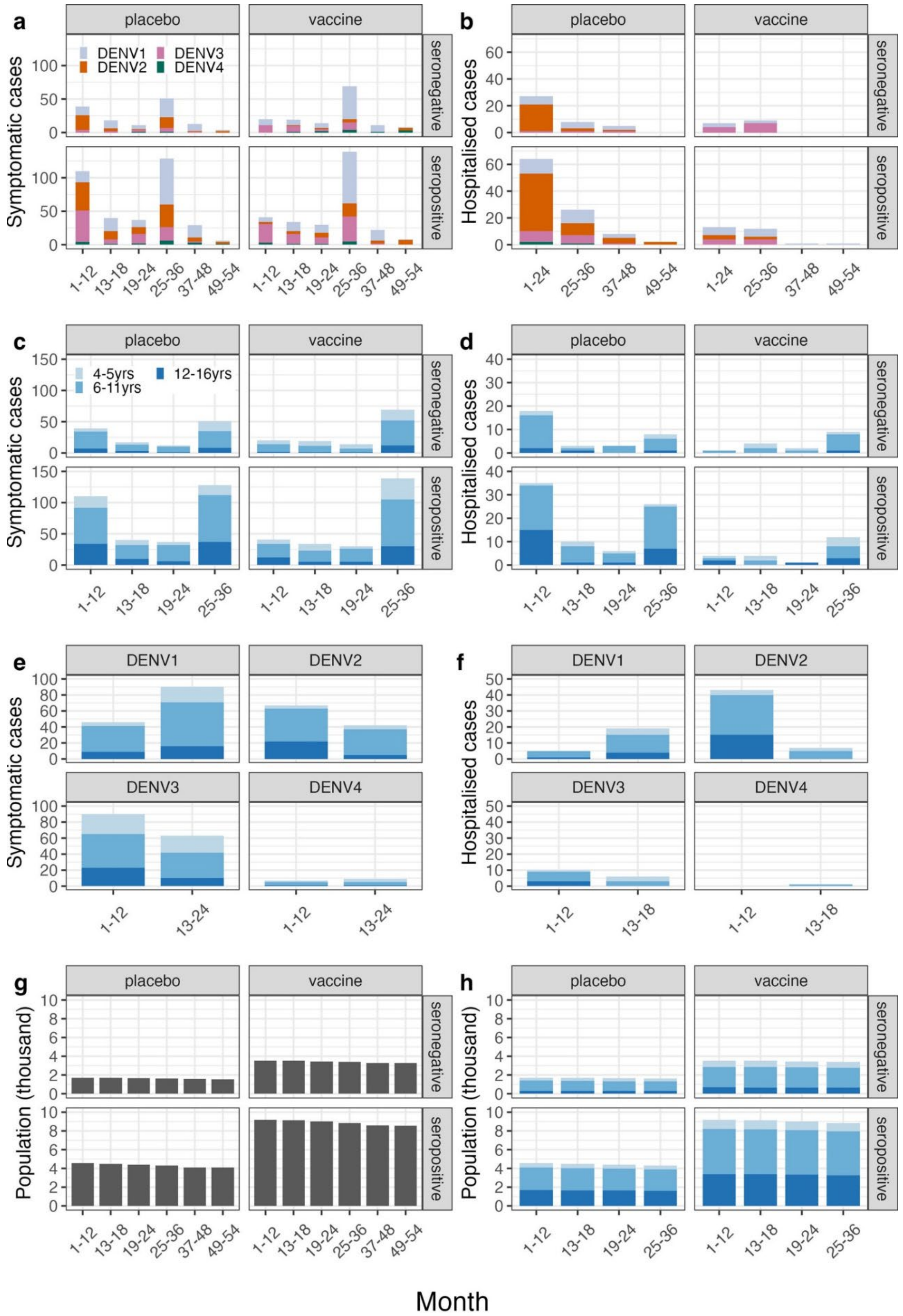

Supplementary Figure 2: Summary of the trial data used to calibrate the model. (a, c, e) Symptomatic cases. (b, d, f) Hospitalised cases. (g-h) Population sizes. Note the 2:1 randomisation of individuals to receive the vaccine vs. the placebo. Data are from the per-protocol population (individuals without any major protocol violations, including not receiving both doses of the correct assignment of Qdenga or placebo) except for the serotype-specific hospitalisation case data in months 1-24 (**b**) which is from the safety population (individuals who received at least one dose of the vaccine or placebo).

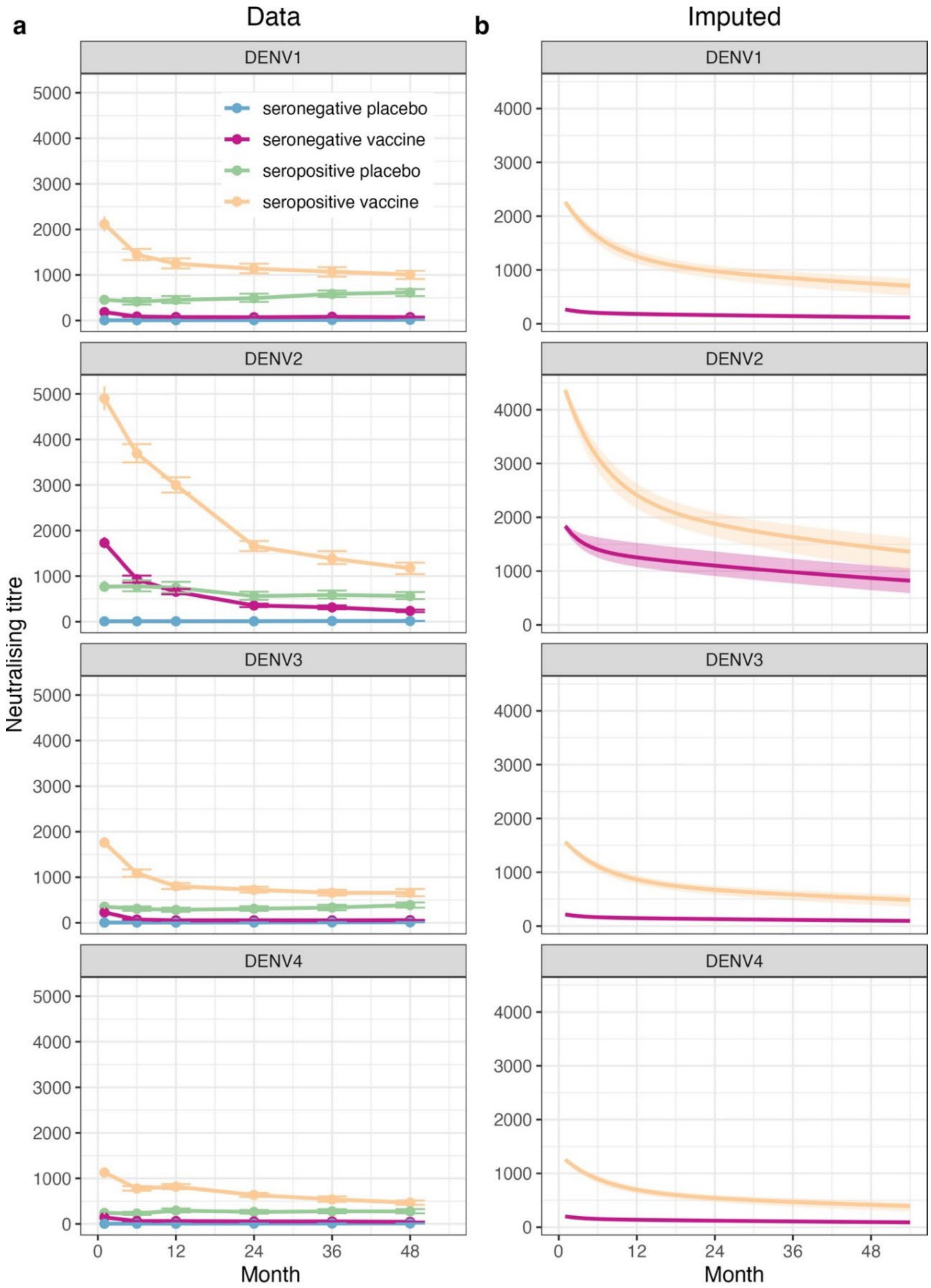

Supplementary Figure 3: Neutralising antibody titres. (a) Observed mean (point) and 95% confidence interval (error bar) neutralising antibody titres by serostatus and trial arm (colours) against each serotype (rows). **(b)** Imputed mean (solid line) and 95% credible interval (shaded region) of the posterior distribution (n=20,000 samples) of the neutralising antibody titres in the vaccine trial arm by serostatus (colours) and serotype (rows).

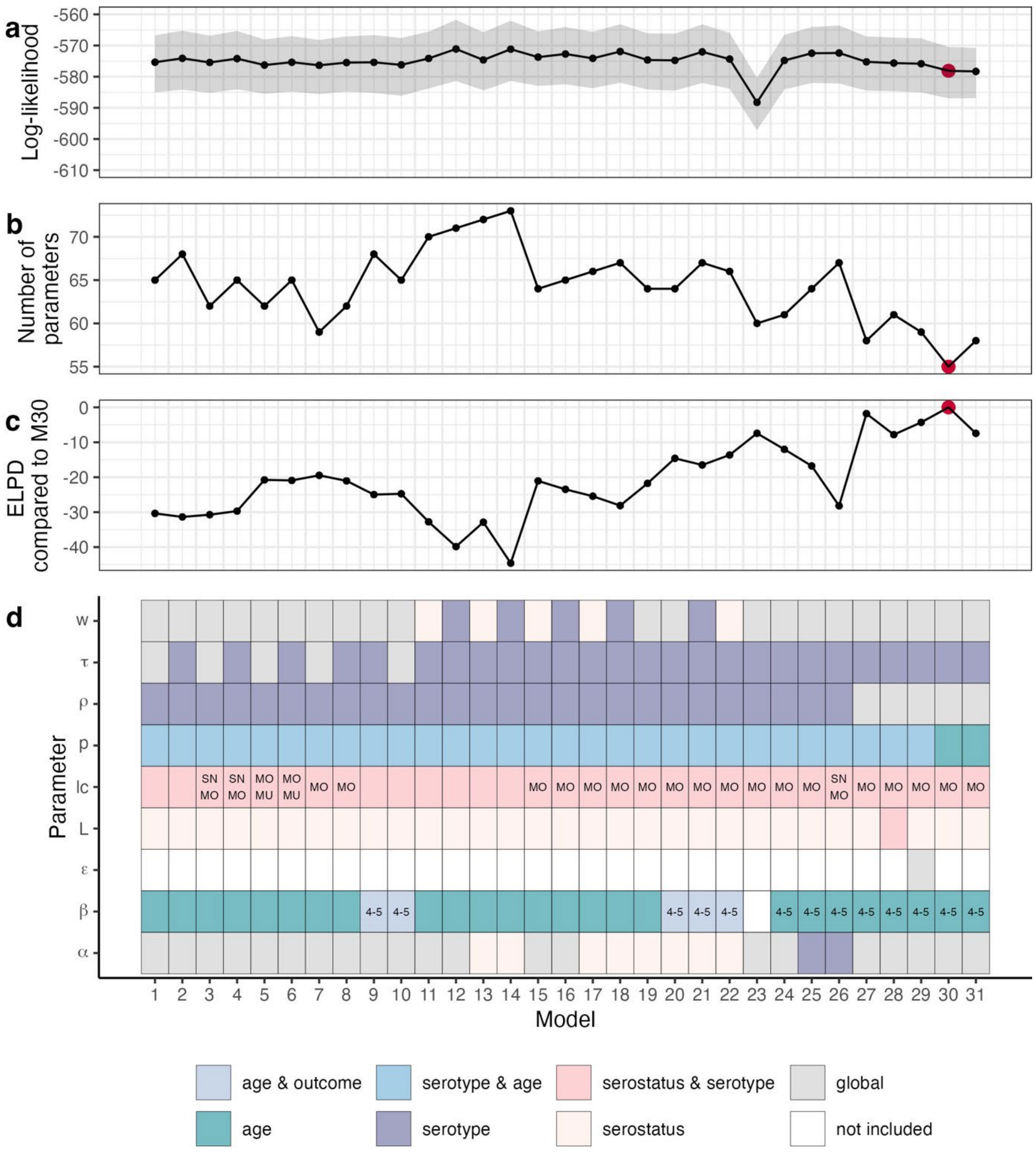

Supplementary Figure 4: (a) Log-likelihood, (b) number of parameters, (c) expected log predictive density (ELPD) compared to the main model and (d) visual description of the estimated parameters by model variant. See Supplementary Table 3 for a full description of the model parameters. The final model (30) is shown in red.

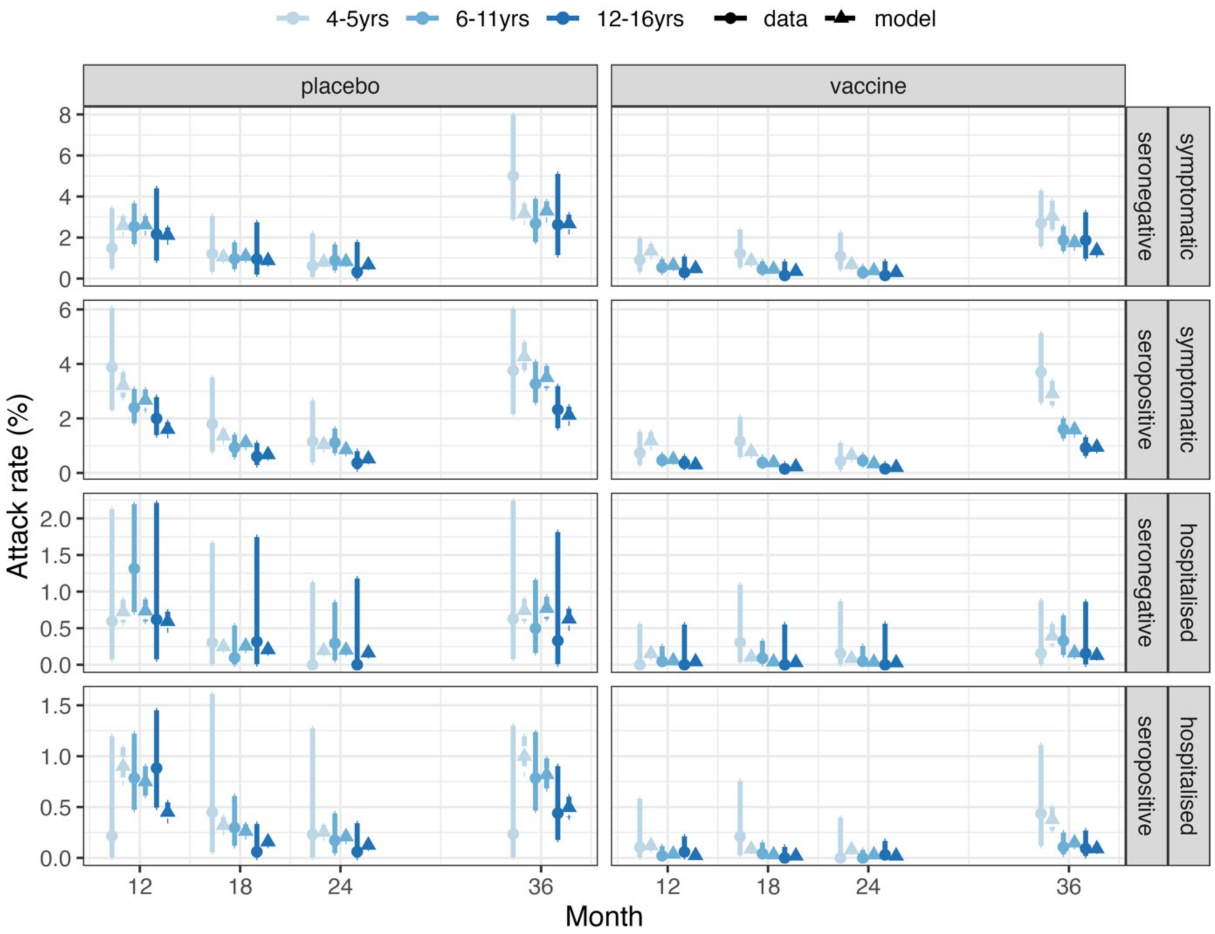

Supplementary Figure 5: Model calibration to age-specific case data. Observed and estimated symptomatic and hospitalised attack rates during the phase III clinical trial by trial arm, serostatus, age group, and time. The modelled attack rates show the mean (triangle) and 95% credible interval (dashed line) of the posterior distribution (n=20,000 samples). The observed attack rates show the mean (circle) and 95% exact binomial confidence interval (solid line).

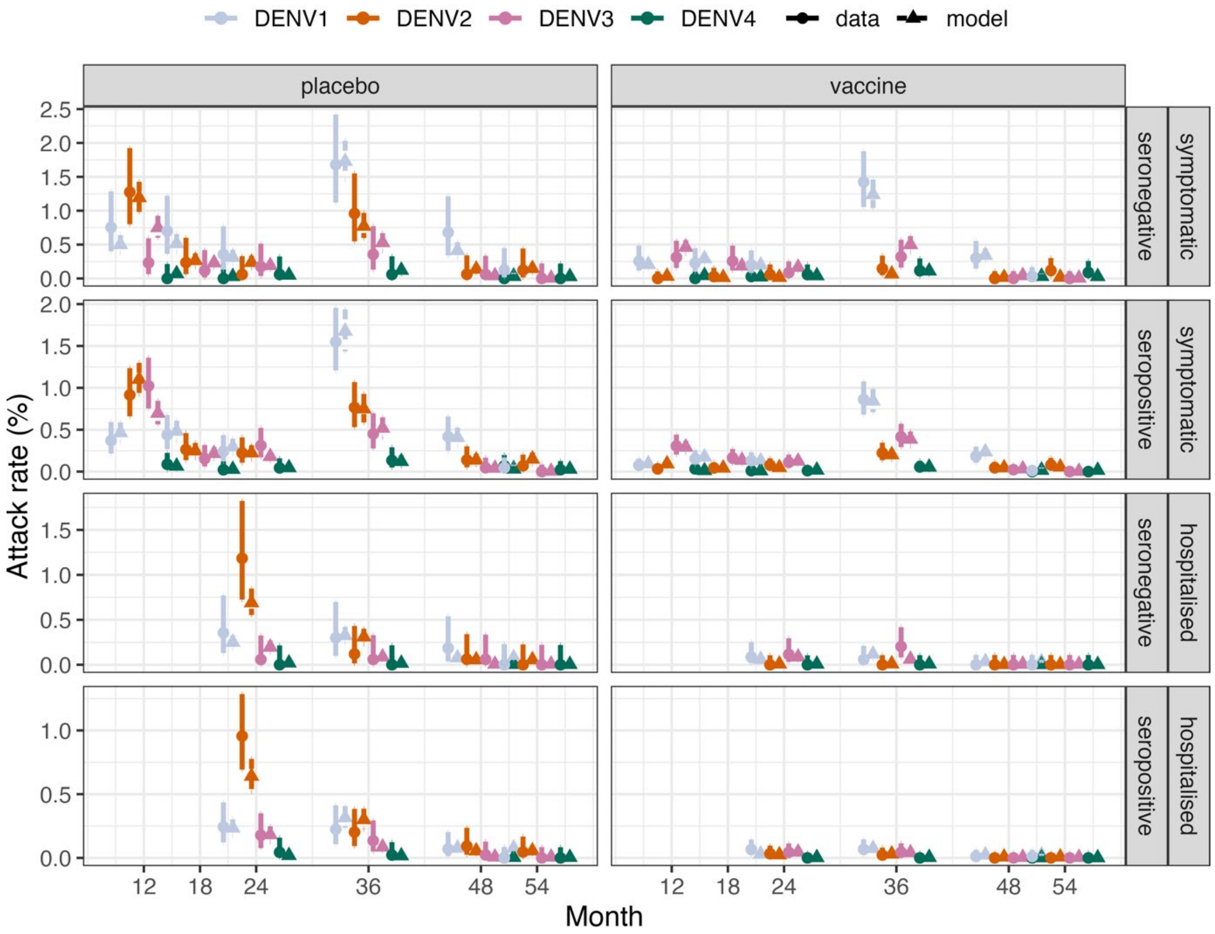

Supplementary Figure 6: Model calibration to serotype-specific case data. Observed and estimated symptomatic and hospitalised attack rates during the phase III clinical trial by serostatus, trial arm, serotype, and time. The modelled attack rates show the mean (triangle) and 95% credible interval (dashed line) of the posterior distribution (n=20,000 samples). The observed attack rates show the mean (circle) and 95% exact binomial confidence interval (solid line).

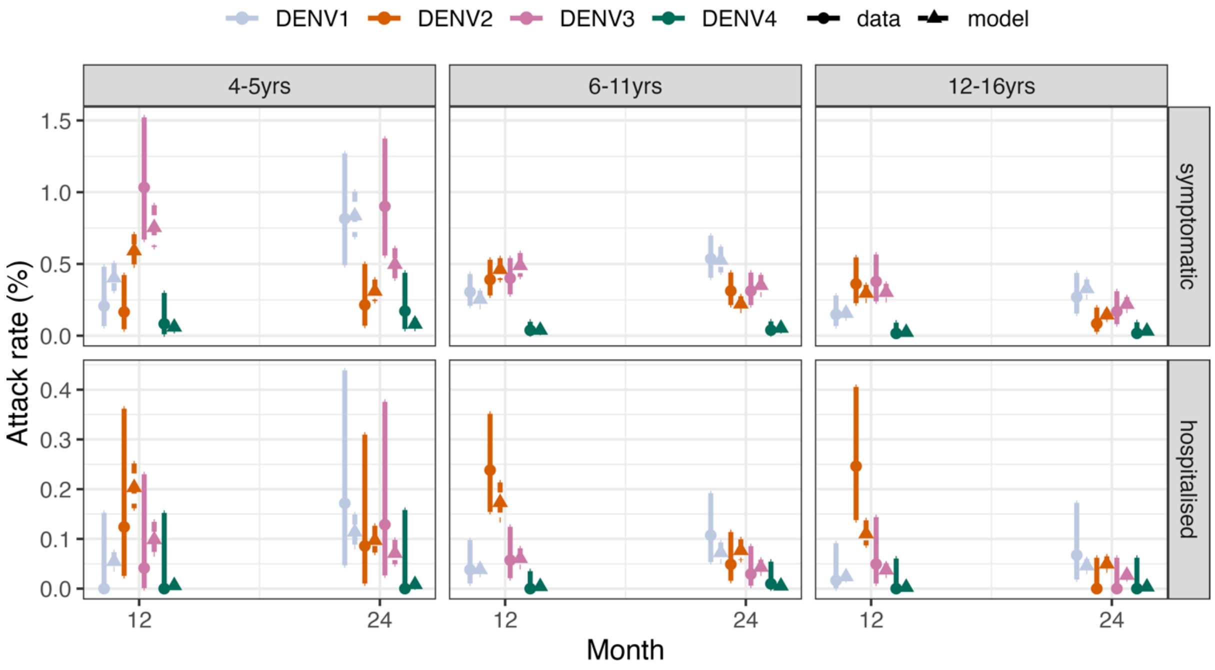

Supplementary Figure 7: Model calibration to age- and serotype-specific case data. Observed and estimated symptomatic and hospitalised attack rates during the phase III clinical trial by serotype, age group, and time. The modelled attack rates show the mean (triangle) and 95% credible interval (dashed line) of the posterior distribution (n=20,000 samples). The observed attack rates show the mean (circle) and 95% exact binomial confidence interval (solid line).

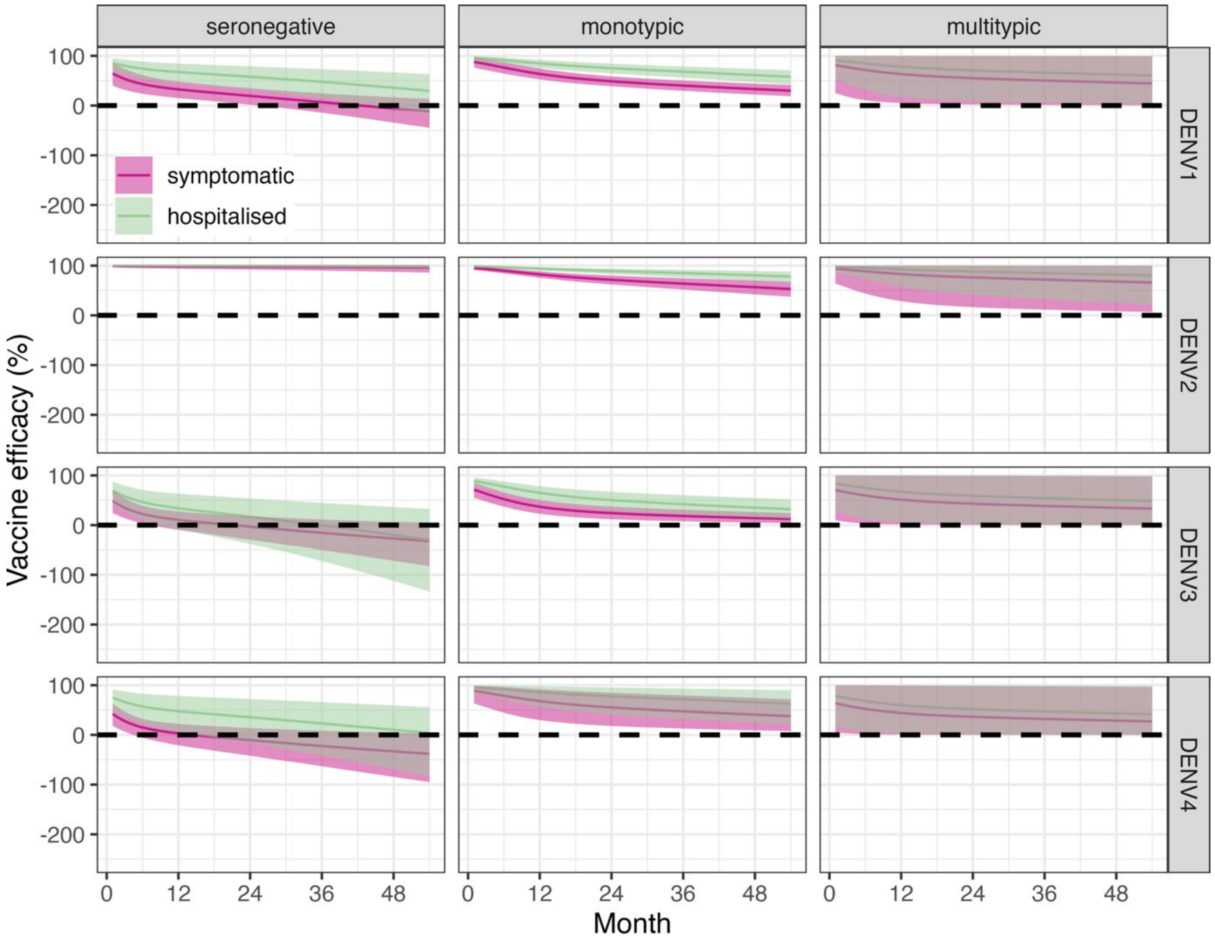

Supplementary Figure 8: Vaccine efficacy estimates. Estimated vaccine efficacy by serostatus (columns) and serotype (rows), against symptomatic disease and hospitalisation (colours). The solid line represents the mean efficacy and the shaded area represents the 95% credible interval of the posterior distribution (n=20,000 samples). The dashed horizontal line marks 0 efficacy.

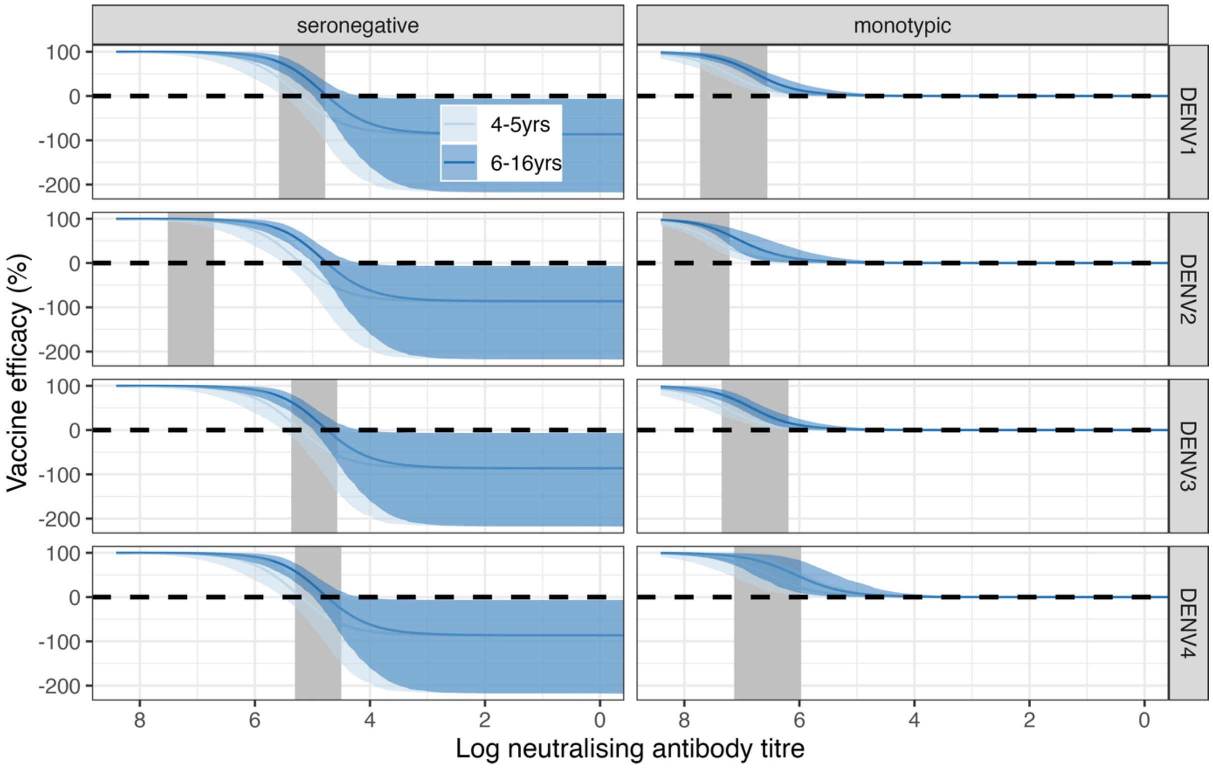

Supplementary Figure 9: Relationship between vaccine efficacy and log neutralising antibody titre. Vaccine efficacy by serostatus (columns), serotype (rows) and age (colours) against symptomatic disease. The solid line represents the mean efficacy, and the shaded area represents the 95% credible interval of the posterior distribution (n=1,000 samples). The dashed horizontal line marks 0 efficacy. The grey shaded regions indicate the range of titres and corresponding vaccine efficacy estimated during the trial duration.

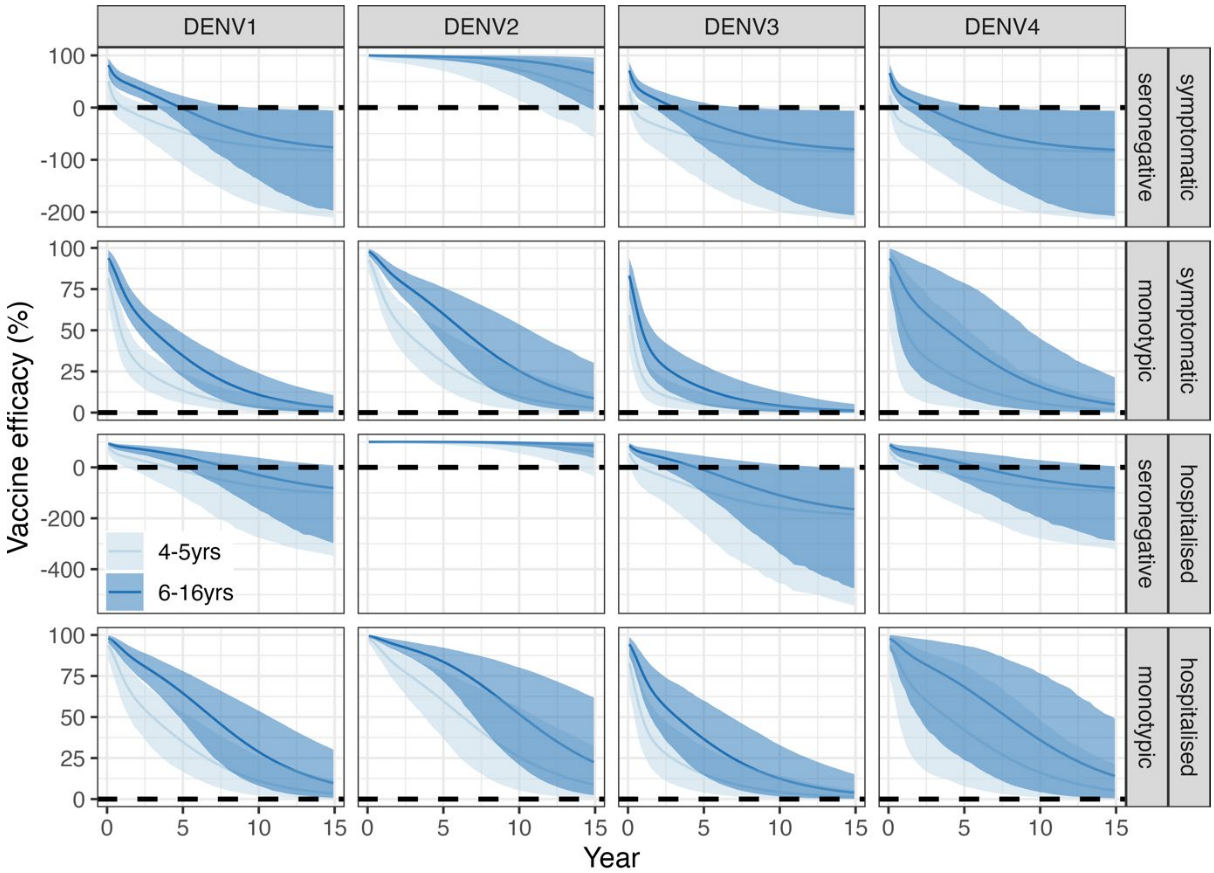

Supplementary Figure 10: Vaccine efficacy estimates projected up to 15 years post-vaccination. Vaccine efficacy by serotype (columns), serostatus (rows) and age (colours) against symptomatic disease and hospitalisation (rows). The solid line represents the mean efficacy and the shaded area represents the 95% credible interval of the posterior distribution (n=1,000 samples). The dashed horizontal line marks 0 efficacy. Efficacy estimates assume the same decay rate observed during the phase III clinical trial. Note the different y-axis scales.

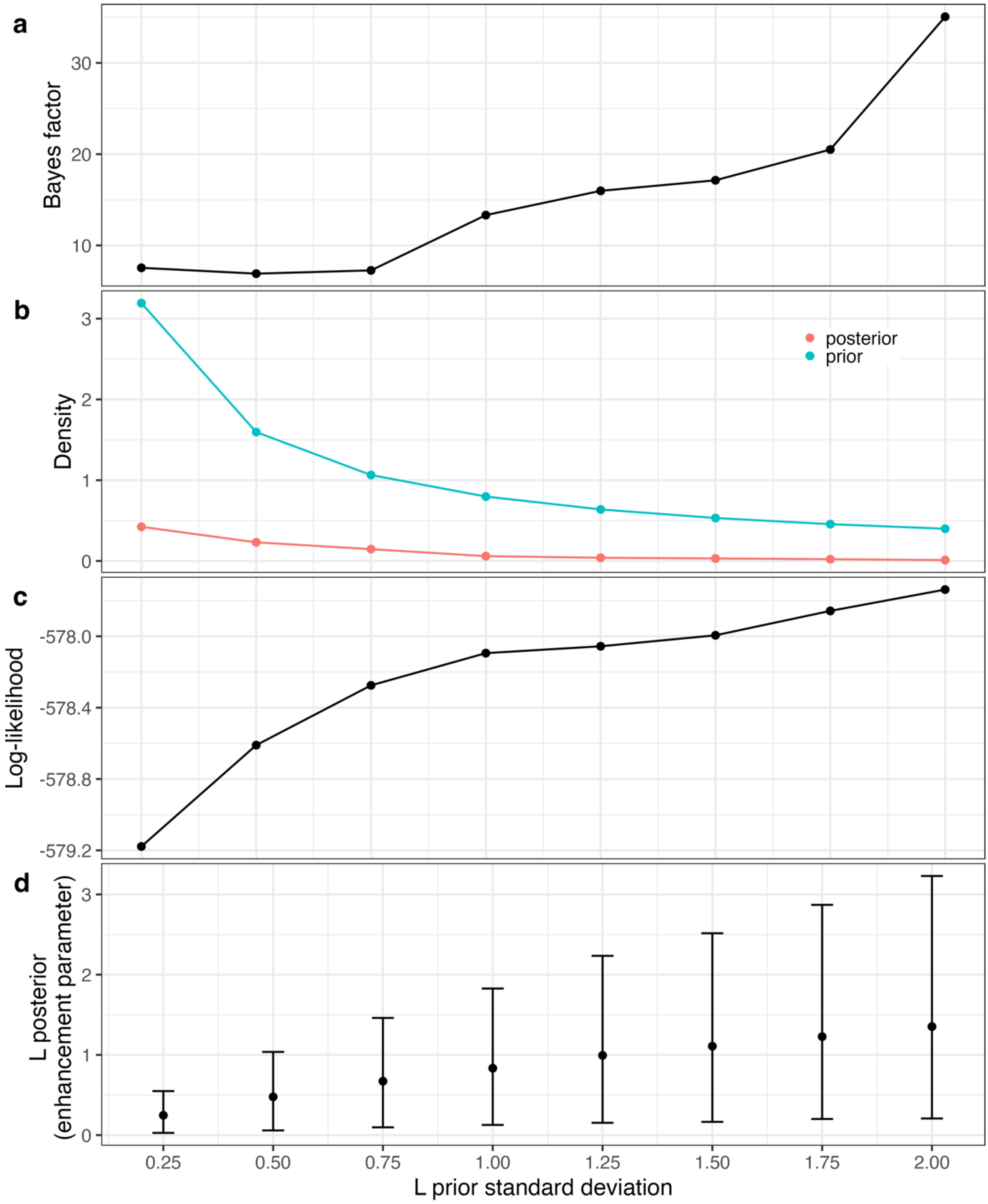

Supplementary Figure 11: Sensitivity analysis on the impact of the choice of the prior distribution of the enhancement parameter (L) on the model support for vaccine-associated enhancement. All prior distributions are truncated normal, centred on 0 (no enhancement). Standard deviations of the prior ranged from 0.25 to 2.00. **(a)** Bayes factor estimates comparing the vaccine efficacy model with and without enhancement (L fixed at 0). **(b)** Mean of the prior and posterior densities at 0. **(c)** Mean log-likelihood of the model with enhancement. **(d)** Posterior mean (point) and 95% credible interval (error bar) of the enhancement parameter L (n = 20,000 samples).

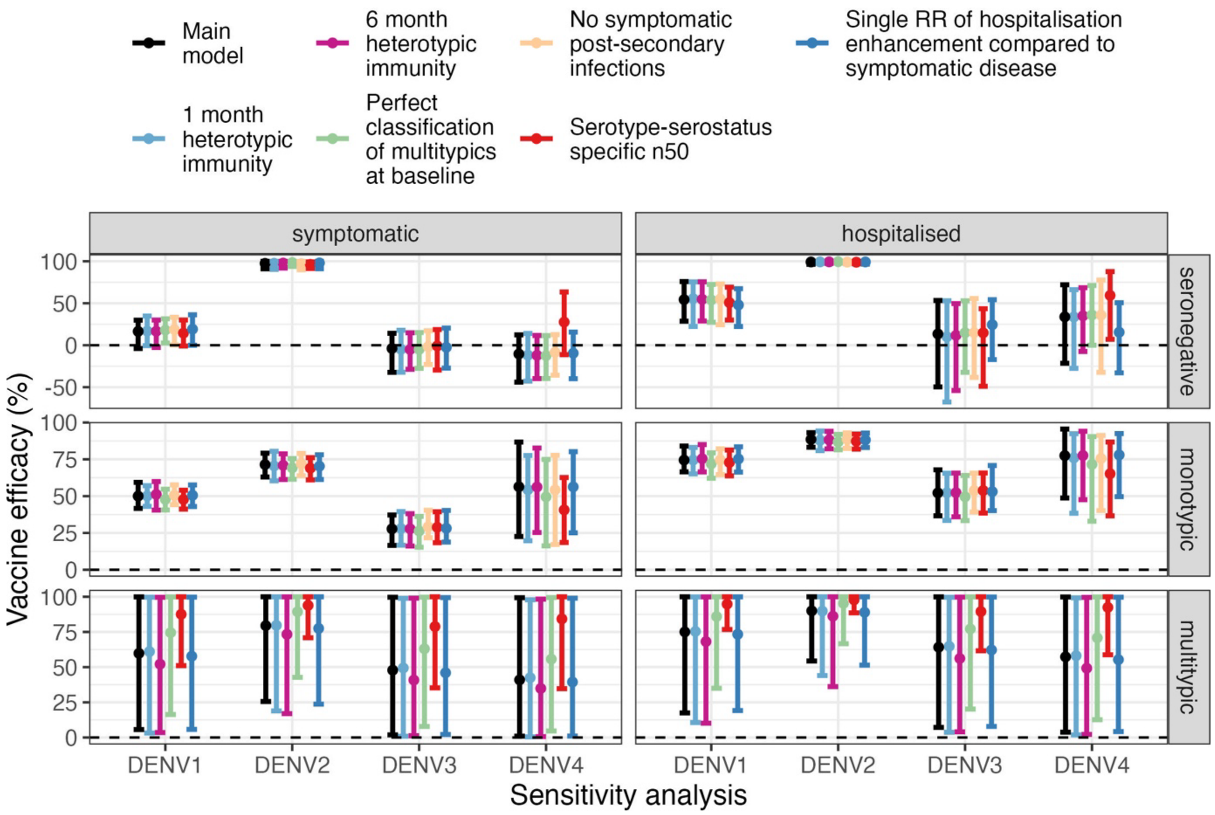

Supplementary Figure 12: **Vaccine efficacy model sensitivity analysis**. Vaccine efficacy estimates for each sensitivity analysis (colours) by serotype (x-axis), serostatus (rows), against symptomatic disease and hospitalisation (columns). The main model assumes that heterotypic immunity lasts 12 months, multitypic individuals can be misclassified as seronegative at baseline, post-secondary infections can be symptomatic, single estimates for the titres required for 50% protection against symptomatic dengue (n_{50}) in seronegative and multitypic individuals but serotype-specific n_{50} values in monotypic individuals, and a serotype-specific risk ratio (RR) of vaccine-associated hospitalisation enhancement compared to symptomatic disease. Point and error bars are respectively the mean and 95% credible interval of n= 100 posterior distribution samples. The dashed horizontal line marks 0 efficacy.

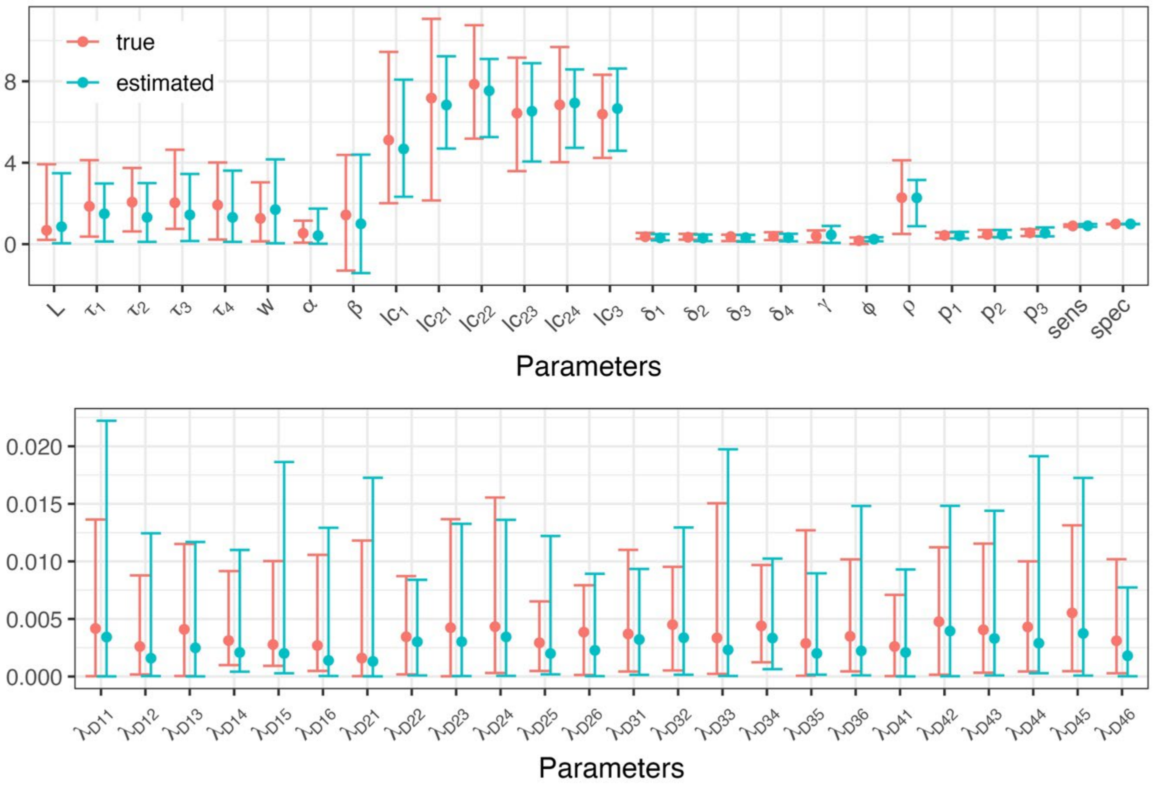

Supplementary Figure 13: **Model validation to simulated case data.** Red point and error bars represent respectively the median and 95% confidence interval of 20 true parameter sets used to simulate the case data. Blue point and error bars are respectively the median and 95% credible interval of the posterior distribution obtained by fitting the model to the simulated case data (n=20). See **Supplementary Table 3** for a full description of the parameters used.

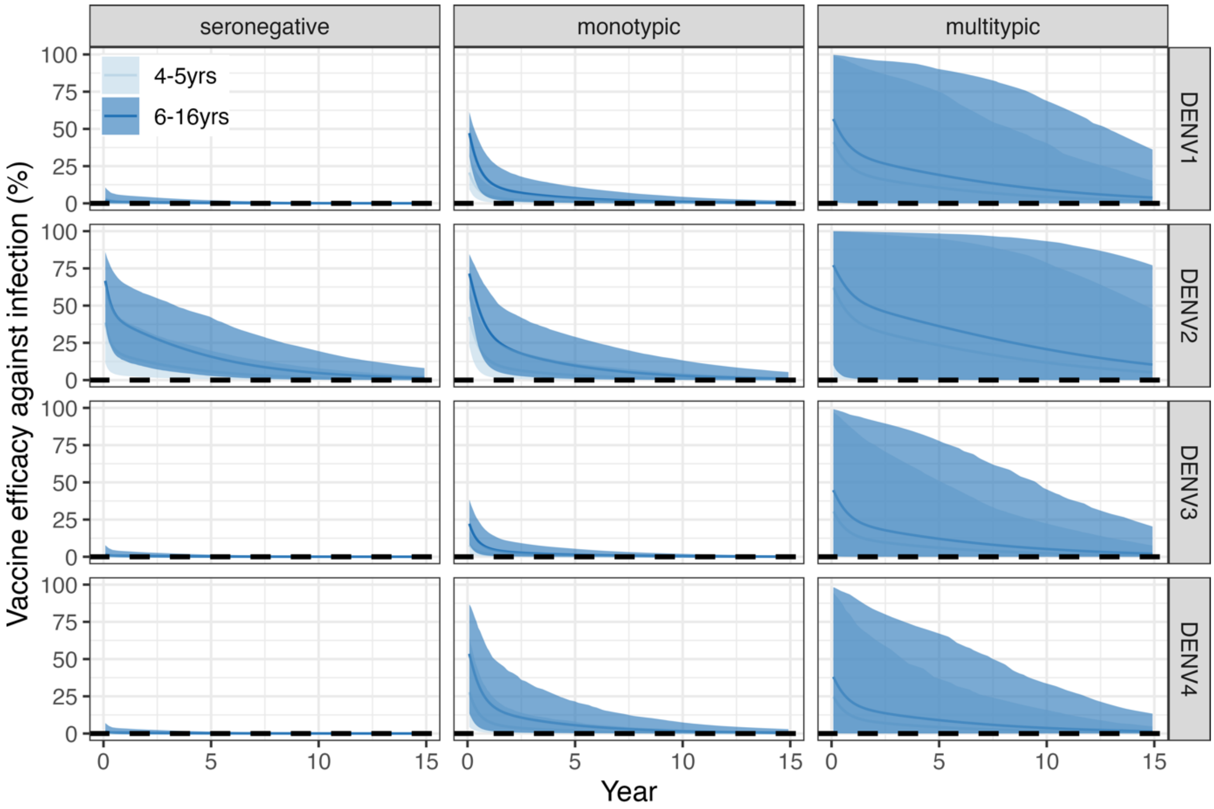

Supplementary Figure 14: Vaccine efficacy against infection scenario. Assumed VE against infection by age (colours), serotype (rows) and serostatus (columns). The solid line represents the mean and the shaded area represents the 95% CrI of the posterior distribution (n=1,000 samples). The dashed horizontal line marks 0 efficacy.

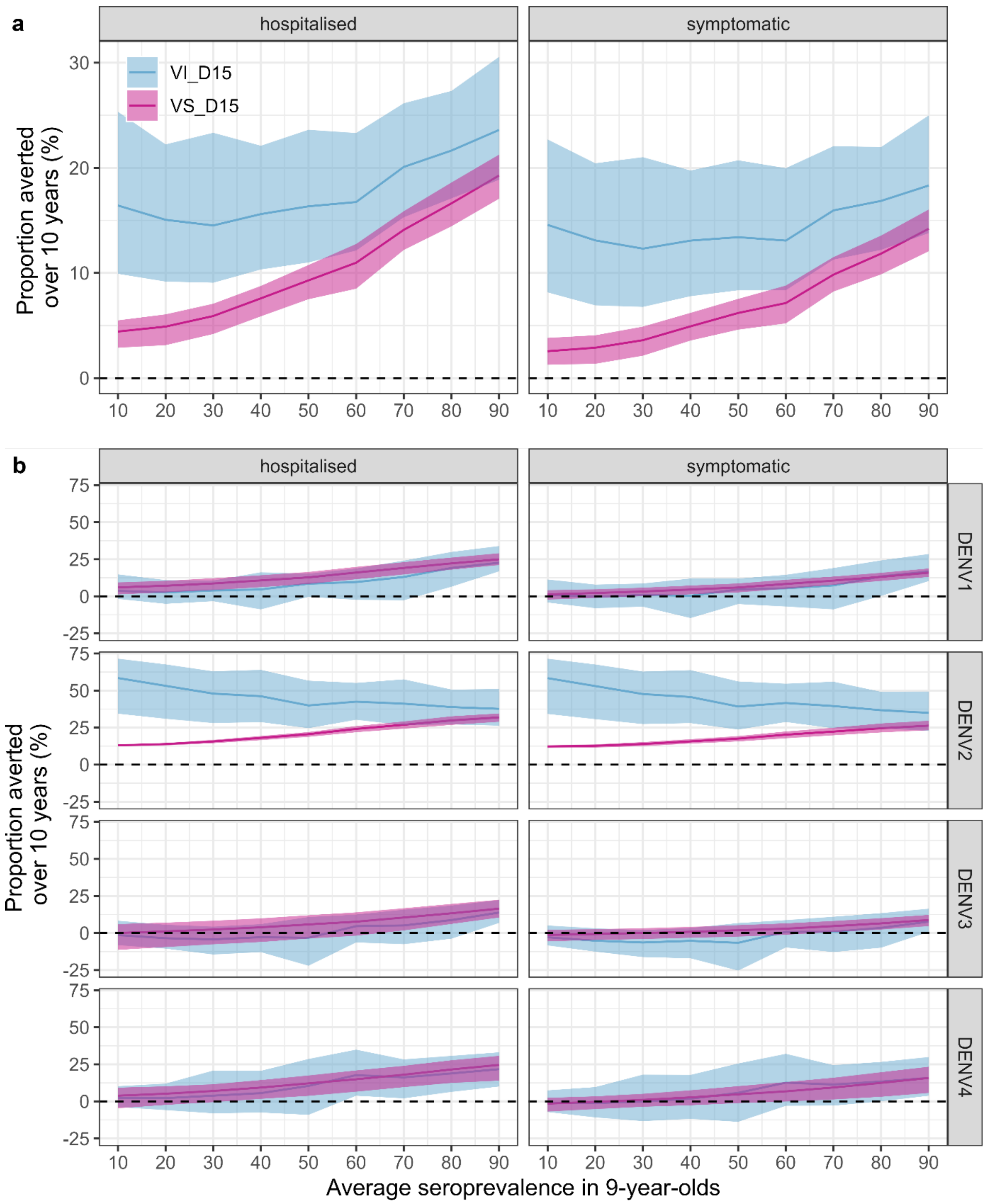

Supplementary Figure 15: Population-level impact of vaccination in the Philippines. Cumulative proportion of hospitalised and symptomatic cases averted (y-axis) by transmission setting (x-axis, expressed as the average seroprevalence in 9-year-olds), assuming efficacy against infection and disease (VI, blue) or only disease (VS, pink) decaying for 15 years (D15), using 80% coverage across ten years and the Philippines demography **(a)** over all serotypes and **(b)** by serotype. The solid line represents the mean, and the shaded regions represent the overall uncertainty (95% CrI) derived from n=10,000 simulations (= 200 posterior distribution samples x 50 stochastic simulations per sample, see Methods for details)

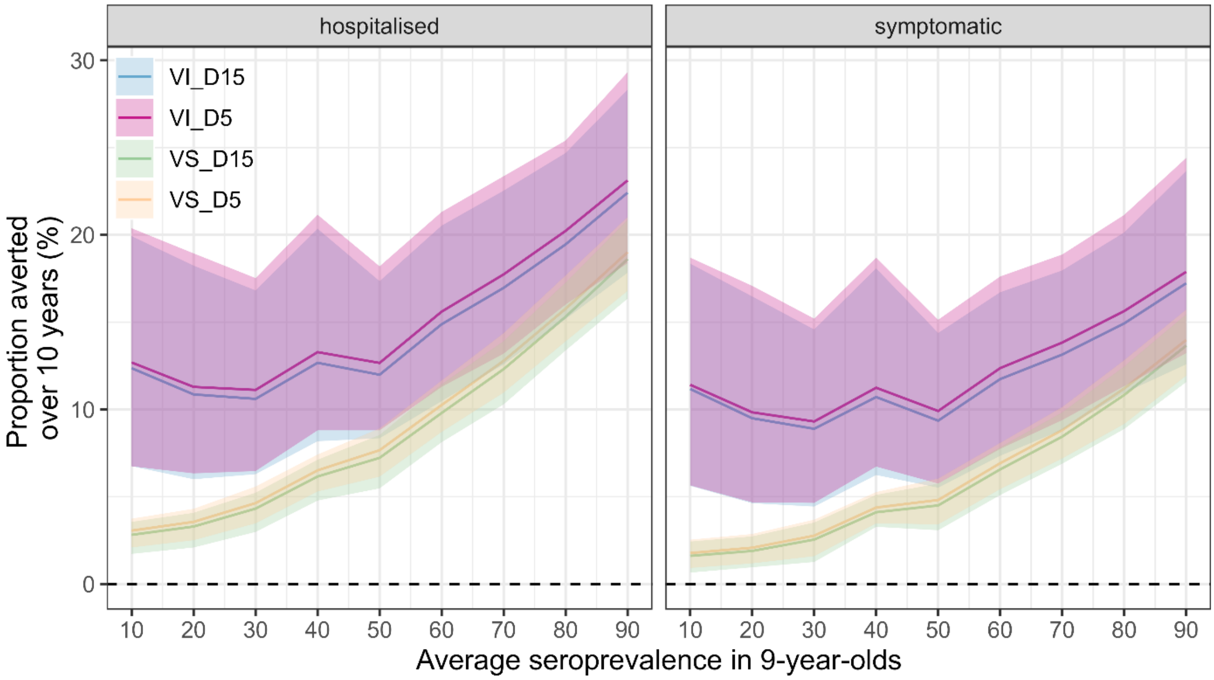

Supplementary Figure 16: Population-level impact of vaccination in Brazil by the different scenarios of vaccine mechanism of action. Cumulative proportion of hospitalised and symptomatic cases averted (y-axis) by transmission setting (x-axis, expressed as the average seroprevalence in 9-year-olds), assuming 80% coverage across ten years, assuming efficacy against infection and disease decaying for 5 years (VI_D5, pink), infection and disease decaying for 15 years (VI_D15, blue), only disease decaying for 5 years (VS_D5, yellow), only disease decaying for 15 years (VS_D15, green). The solid line represents the mean, and the shaded regions represent the overall uncertainty (95% CrI) derived from n=10,000 simulations (= 200 posterior distribution samples x 50 stochastic simulations per sample, see Methods for details)

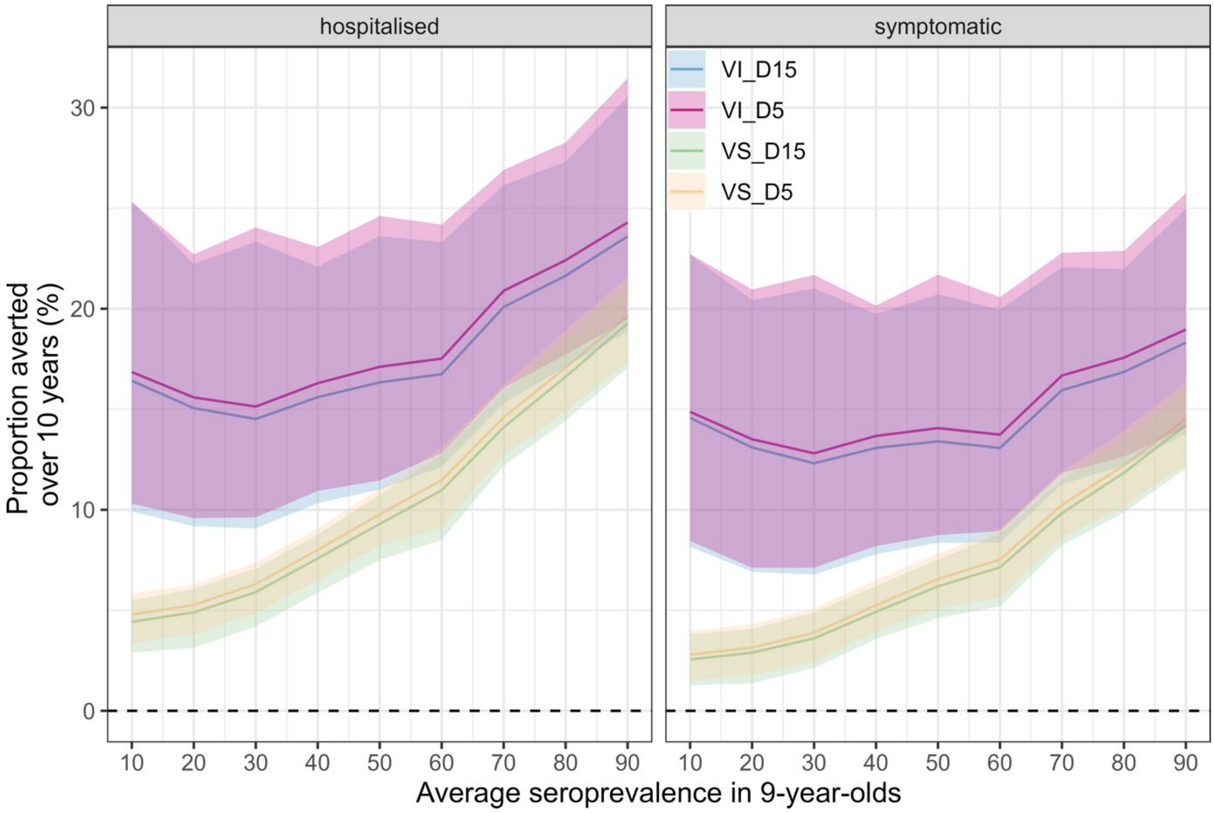

Supplementary Figure 17: Population-level impact of vaccination in the Philippines by the different scenarios of vaccine mechanism of action. Cumulative proportion of hospitalised and symptomatic cases averted (y-axis) by transmission setting (x-axis, expressed as the average seroprevalence in 9-year-olds), assuming 80% coverage across ten years, assuming efficacy against infection and disease decaying for 5 years (VI_D5, pink), infection and disease decaying for 15 years (VI_D15, blue), only disease decaying for 5 years (VS_D5, yellow), only disease decaying for 15 years (VS_D15, green). The solid line represents the mean, and the shaded regions represent the overall uncertainty (95% CrI) derived from n=10,000 simulations (= 200 posterior distribution samples x 50 stochastic simulations per sample, see Methods for details)

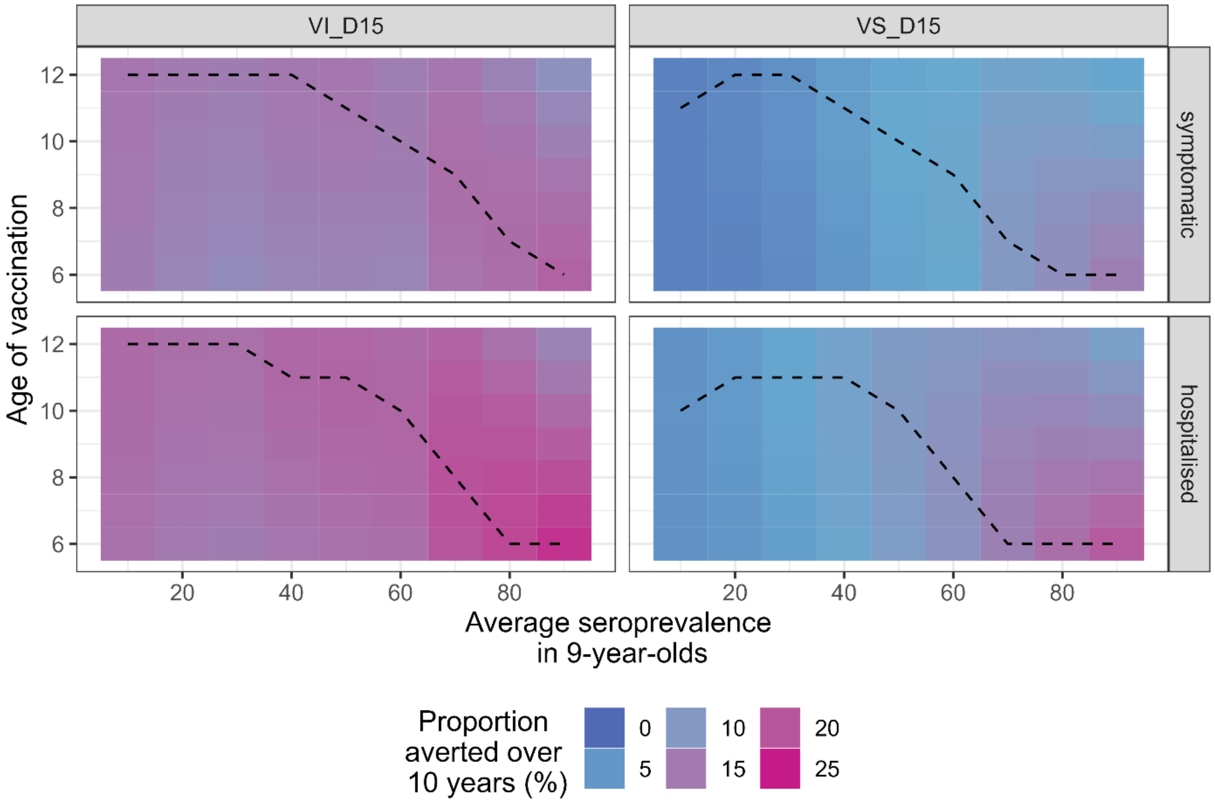

Supplementary Figure 18: Impact of the age at vaccination on the population-level impact in the Philippines by transmission setting. Cumulative proportion of hospitalised and symptomatic cases averted (rows) by transmission setting (x-axis, expressed as the average seroprevalence in 9-year-olds), and vaccine mechanism (columns) assuming vaccination of ages 6-12 (y-axis) and the Philippines demography, over ten years. VI_D15: scenario assuming efficacy against infection and disease decaying for 15 years post vaccination. VS_D15: scenario assuming efficacy against disease decaying for 15 years post vaccination.

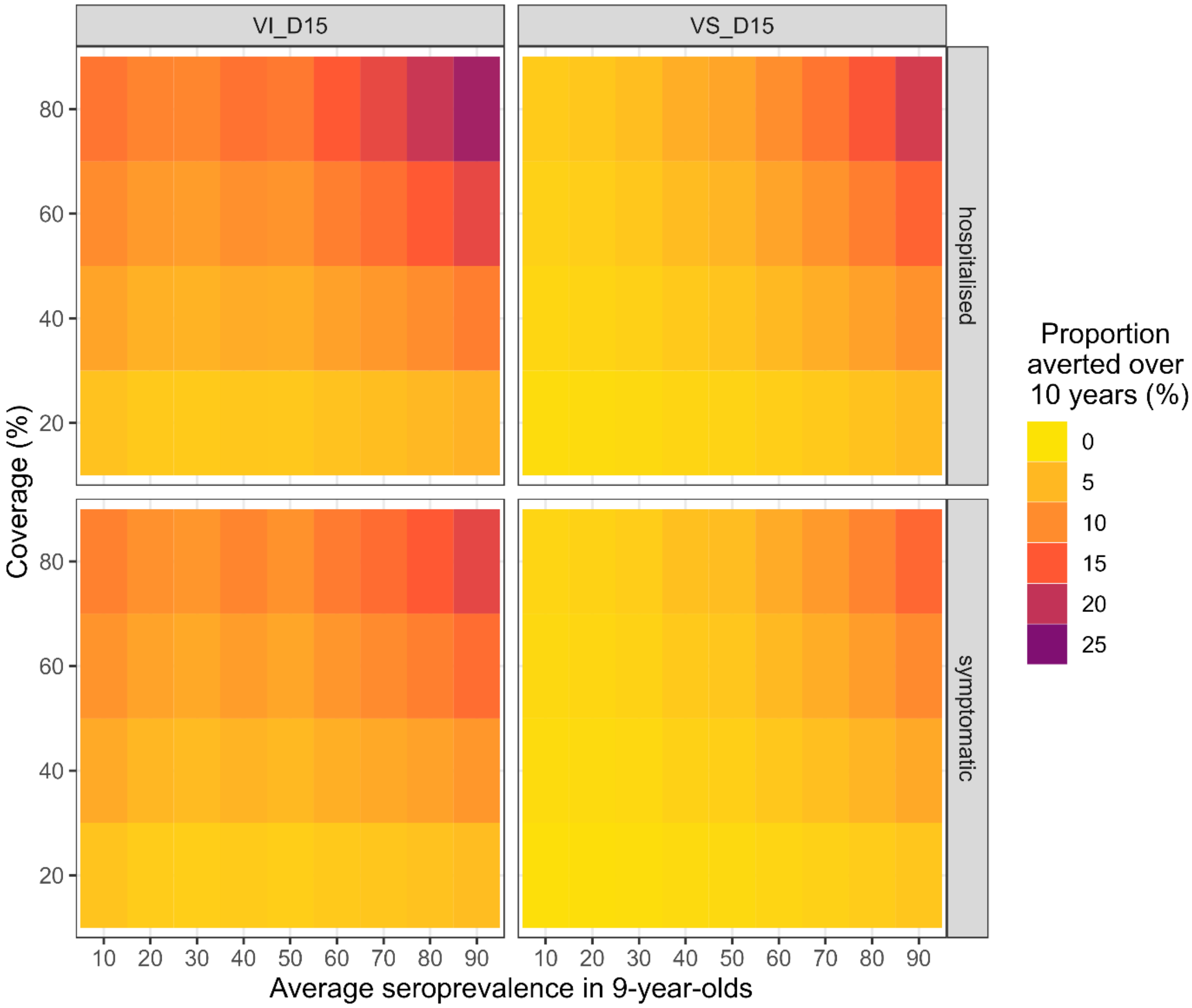

Supplementary Figure 19: Effect of vaccination coverage on the population-level impact in Brazil. Cumulative proportion of hospitalised and symptomatic cases averted (rows) by transmission setting (x-axis, expressed as the average seroprevalence in 9-year-olds), and vaccine mechanism (columns) assuming 20-80% coverage (y-axis) and the Brazilian demography, over ten years.

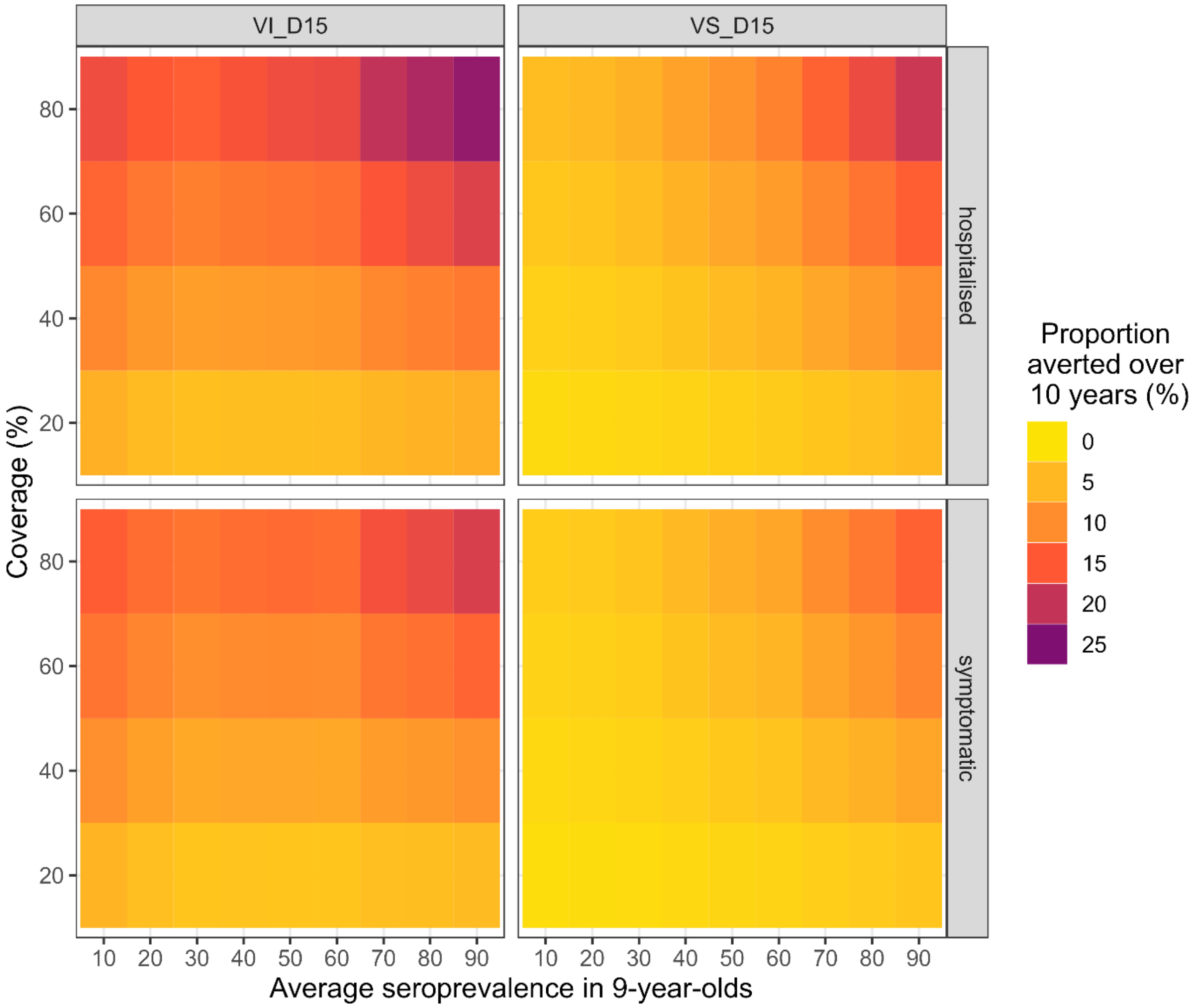

Supplementary Figure 20: Effect of vaccination coverage on the population-level impact in the Philippines. Cumulative proportion of hospitalised and symptomatic cases averted (rows) by transmission setting (x-axis, expressed as the average seroprevalence in 9-year-olds), and vaccine mechanism (columns) assuming 20-80% coverage (y-axis) and the Philippines demography, over ten years.

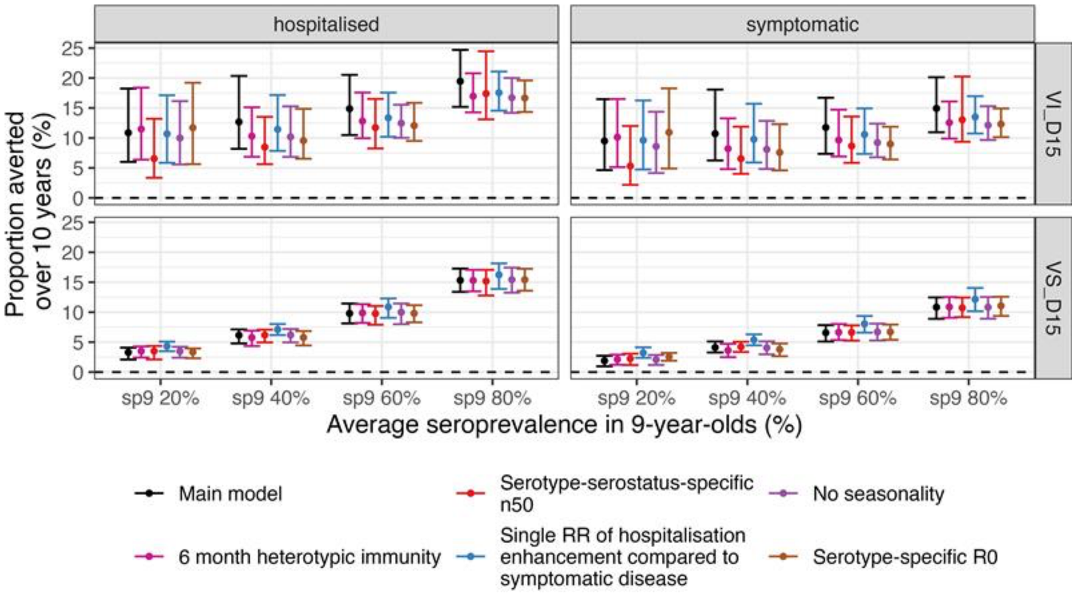

Supplementary Figure 21: Transmission model sensitivity analysis. Proportion of hospitalised and symptomatic cases averted (columns) for each sensitivity analysis (colours) in the first vaccinated cohort of 6-year-olds over ten years by transmission setting, expressed as the average seroprevalence in 9-year-olds (x-axis) assuming a vaccination coverage of 80%, scenarios VS_D15 or VI_D15 (rows) and Brazilian demography. The main model assumes 12-month heterotypic immunity, seasonal dengue transmission, equal transmissibility of all four serotypes, single estimates for the titres required for 50% protection against symptomatic dengue in seronegative and multitypic individuals but serotype-specific values in monotypic individuals, and a serotype-specific risk ratio (RR) of vaccine-associated hospitalisation enhancement compared to symptomatic disease. The points represent the mean and error bars represents the overall uncertainty (95% CrI) derived from n=10,000 simulations (= 200 posterior distribution samples x 50 stochastic simulations per sample, see Methods for details).

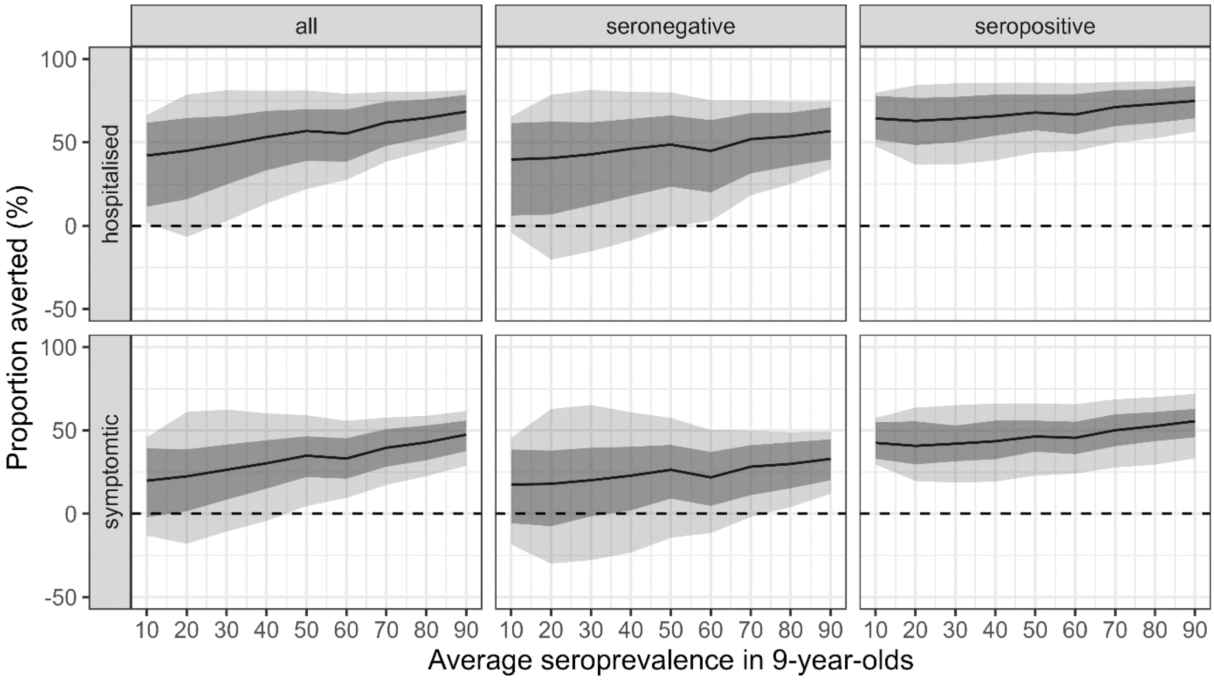

Supplementary Figure 22: Individual-level impact of vaccination in the Philippines assuming efficacy against disease only (VS_D15). Proportion of hospitalised and symptomatic cases averted (rows) in the first vaccinated cohort of 6-year-olds over ten years by transmission setting, expressed as the average seroprevalence in 9-year-olds (x-axis) assuming a vaccination coverage of 80% using model VS_D15 and the Philippines demography. The impact is shown overall (all) and among baseline seropositive and seronegative vaccinees (columns). The solid lines represent the mean, light shading represents the overall uncertainty (95% CrI), derived from n=10,000 simulations (= 200 posterior distribution samples x 50 stochastic simulations per sample, see Methods for details), and the dark shading represents the parameter uncertainty (95% CrI), derived from 200 posterior distribution samples.

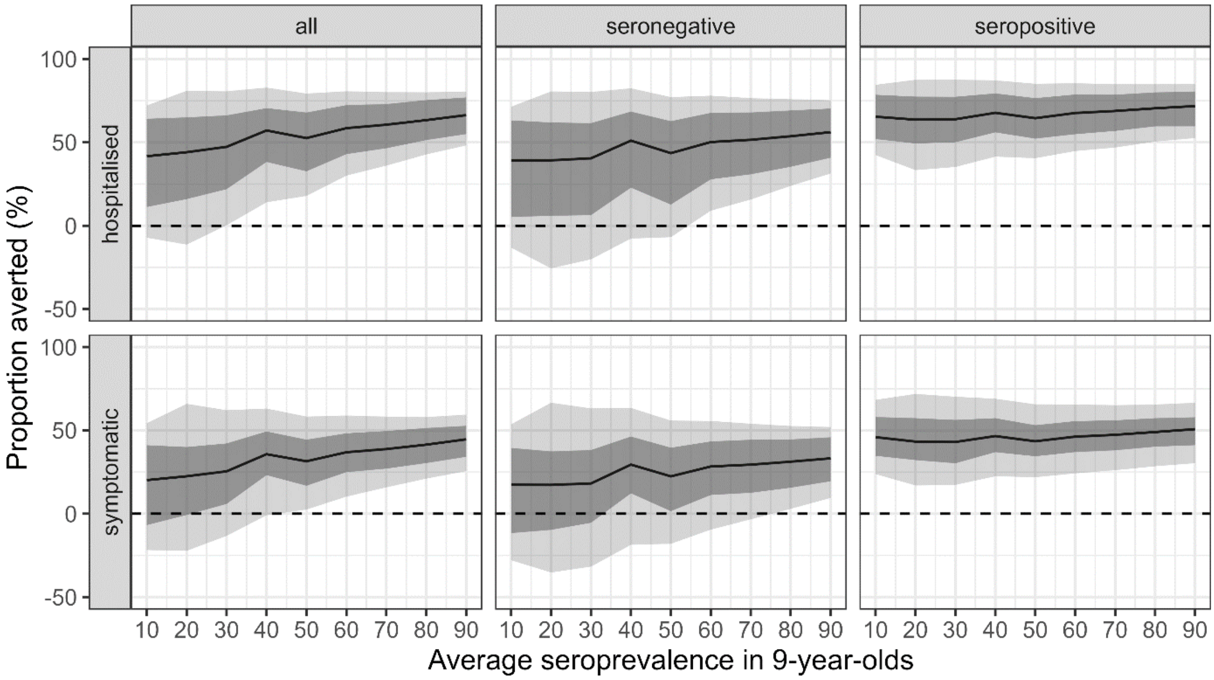

Supplementary Figure 23: Individual-level impact of vaccination in Brazil assuming vaccine efficacy against infection and disease (VI_D15). Proportion of hospitalised and symptomatic cases averted (rows) in the first vaccinated cohort of 6-year-olds over ten years by transmission setting, expressed as the average seroprevalence in 9-year-olds (x-axis) assuming a vaccination coverage of 80% using model VI_D15 and the Brazil demography. The impact is shown overall (all) and among baseline seropositive and seronegative vaccinees (columns). The solid lines represent the mean, light shading represents the overall uncertainty (95% CrI), derived from n=10,000 simulations (= 200 posterior distribution samples x 50 stochastic simulations per sample, see Methods for details), and the dark shading represents the parameter uncertainty (95% CrI), derived from 200 posterior distribution samples.

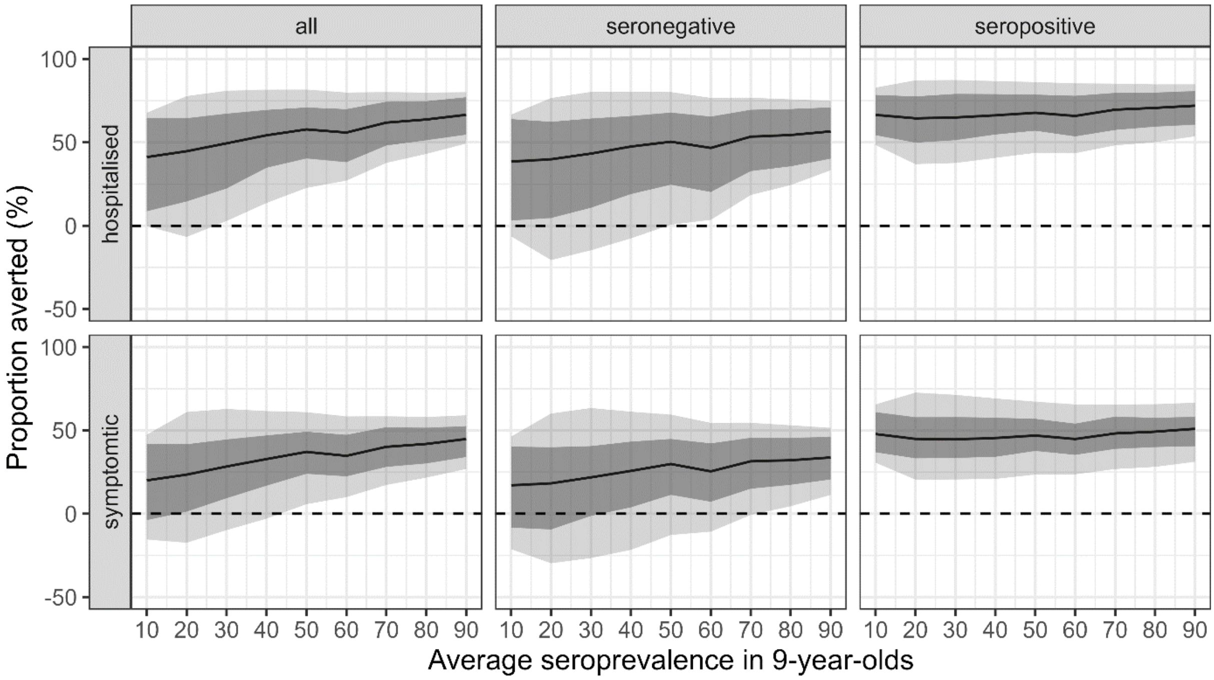

Supplementary Figure 24: Individual-level impact of vaccination in the Philippines assuming vaccine efficacy against infection and disease (VI_D15). Proportion of hospitalised and symptomatic cases averted (rows) in the first vaccinated cohort of 6-year-olds over ten years by transmission setting, expressed as the average seroprevalence in9-year-olds (x-axis) assuming a vaccination coverage of 80% using model VI_D15 and the Philippines demography. The impact is shown overall (all) and among baseline seropositive and seronegative vaccinees (columns). The solid lines represent the mean, light shading represents the overall uncertainty (95% CrI), derived from n=10,000 simulations (= 200 posterior distribution samples x 50 stochastic simulations per sample, see Methods for details), and the dark shading represents the parameter uncertainty (95% CrI), derived from 200 posterior distribution samples.

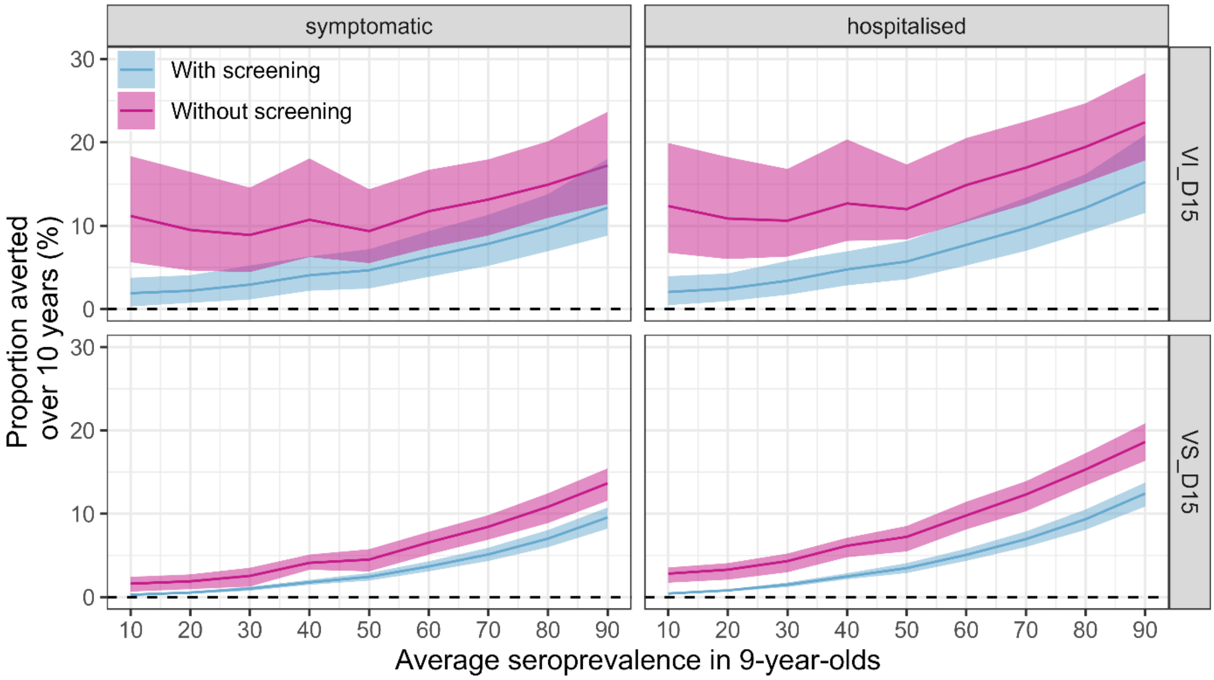

Supplementary Figure 25: Population impact of pre-vaccination screening in Brazil. Proportion of symptomatic cases and hospitalisations averted (y-axis) with (blue) and without (pink) pre-vaccination screening, over 10 years since the start of routine vaccination in the entire population by transmission setting (x-axis, expressed as the average seroprevalence in9-year-olds), assuming 80% coverage and scenario VS_D15 or VI_D15 (rows). The solid lines represent the mean and shaded region represents the overall uncertainty (95% CrI) derived from n=10,000 simulations (= 200 posterior distribution samples x 50 stochastic simulations per sample, see Methods for details).

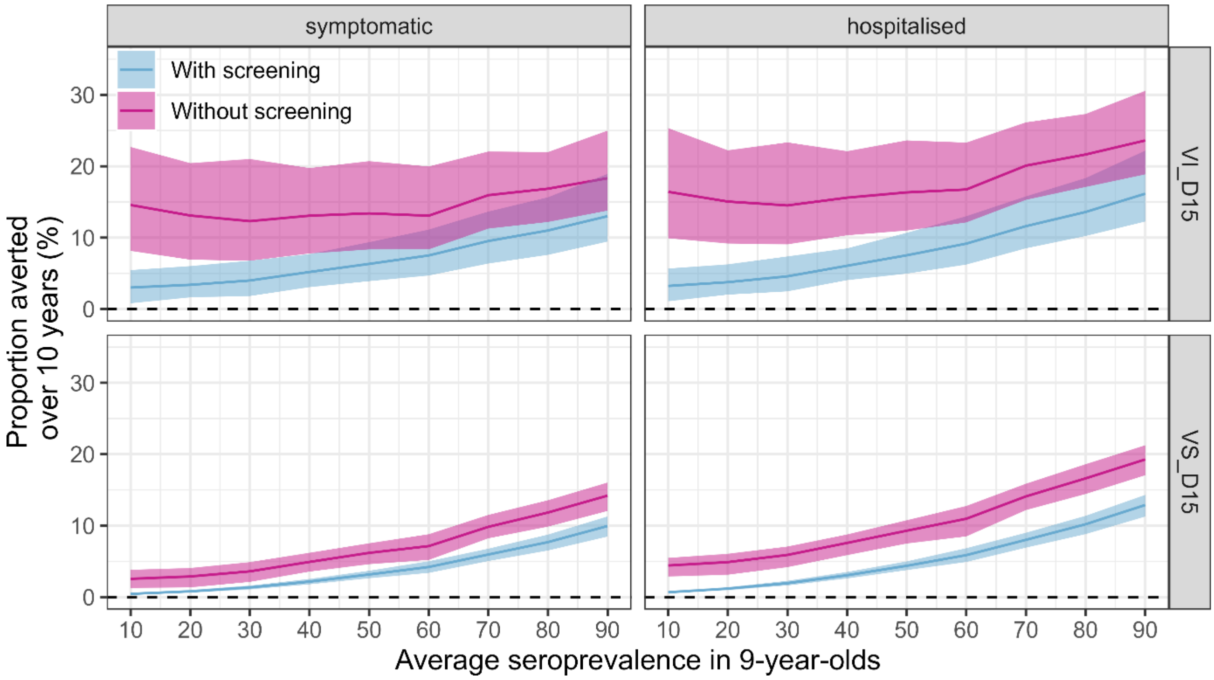

Supplementary Figure 26: Population impact of pre-vaccination screening in the Philippines. Proportion of symptomatic cases and hospitalisations averted (y-axis) with (blue) and without (pink) pre-vaccination screening, over 10 years since the start of routine vaccination in the entire population by transmission setting (x-axis, expressed as the average seroprevalence in 9-year-olds), assuming 80% coverage and scenario VS_D15 or VI_D15 (rows). The solid lines represent the mean and shaded region represents the overall uncertainty (95% CrI) derived from n=10,000 simulations (= 200 posterior distribution samples x 50 stochastic simulations per sample, see Methods for details).

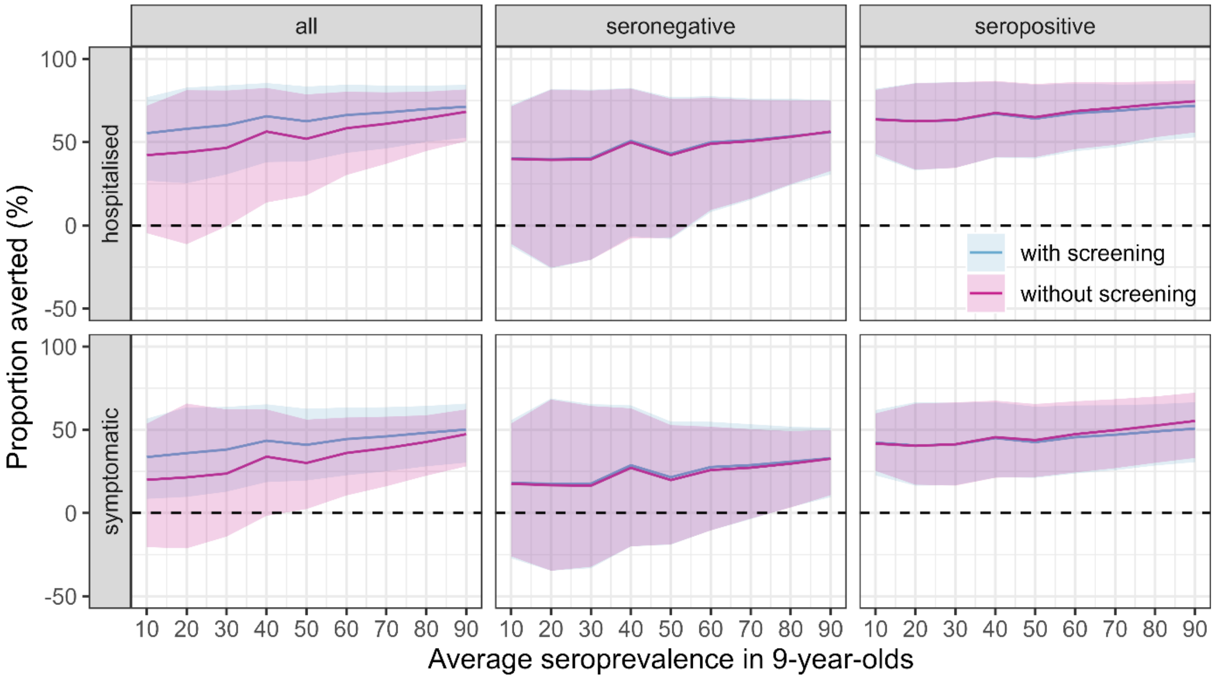

Supplementary Figure 27: Individual-level impact of pre-vaccination screening in Brazil. Proportion of hospitalised and symptomatic cases averted (rows) in the first vaccinated cohort of 6-year-olds over ten years by transmission setting, expressed as the average seroprevalence in9-year-olds (x-axis) assuming a vaccination coverage of 80% using model VS_D15 and the Brazil demography. The impact is shown overall (all) and among baseline seropositive and seronegative vaccinees (columns). The solid lines represent the mean and shaded region represents the overall uncertainty (95% CrI) derived from n=10,000 simulations (= 200 posterior distribution samples x 50 stochastic simulations per sample, see Methods for details).

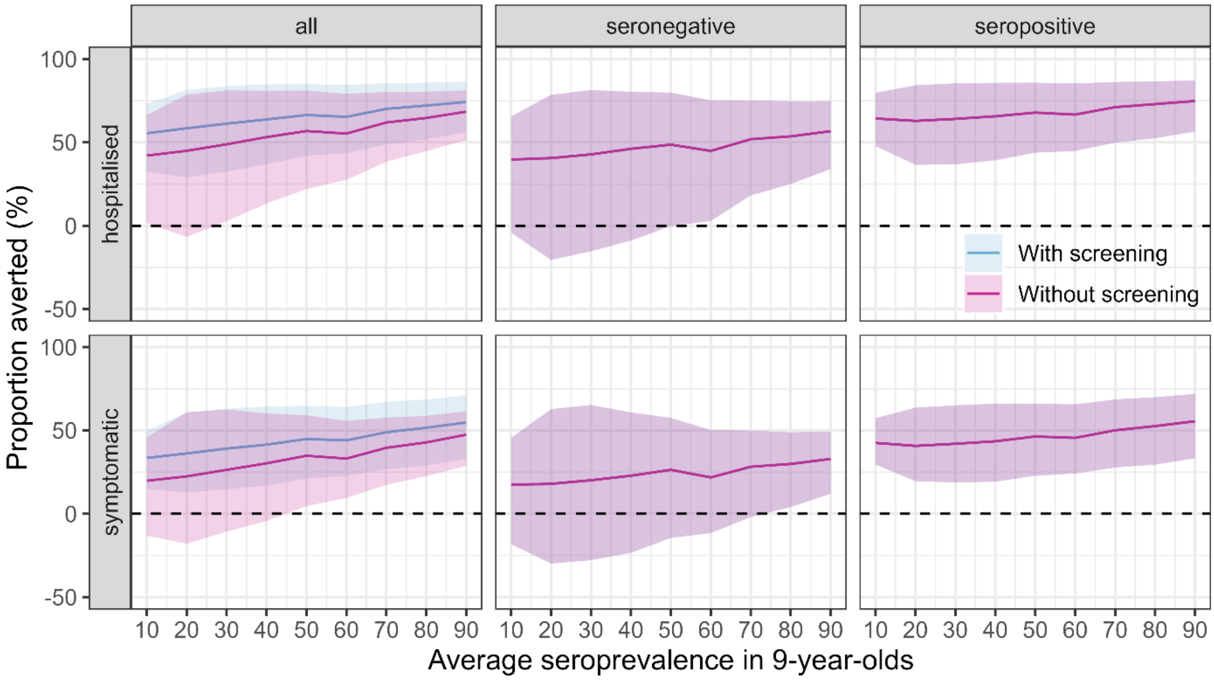

Supplementary Figure 28: Individual-level impact of pre-vaccination screening in the Philippines. Proportion of hospitalised and symptomatic cases averted (rows) in the first vaccinated cohort of 6-year-olds over ten years by transmission setting, expressed as the average seroprevalence in 9-year-olds (x-axis) assuming a vaccination coverage of 80% using model VS_D15 and the Philippines demography. The impact is shown overall (all) and among baseline seropositive and seronegative vaccinees (columns). The solid lines represent the mean and shaded region represents the overall uncertainty (95% CrI) derived from n=10,000 simulations (= 200 posterior distribution samples x 50 stochastic simulations per sample, see Methods for details).

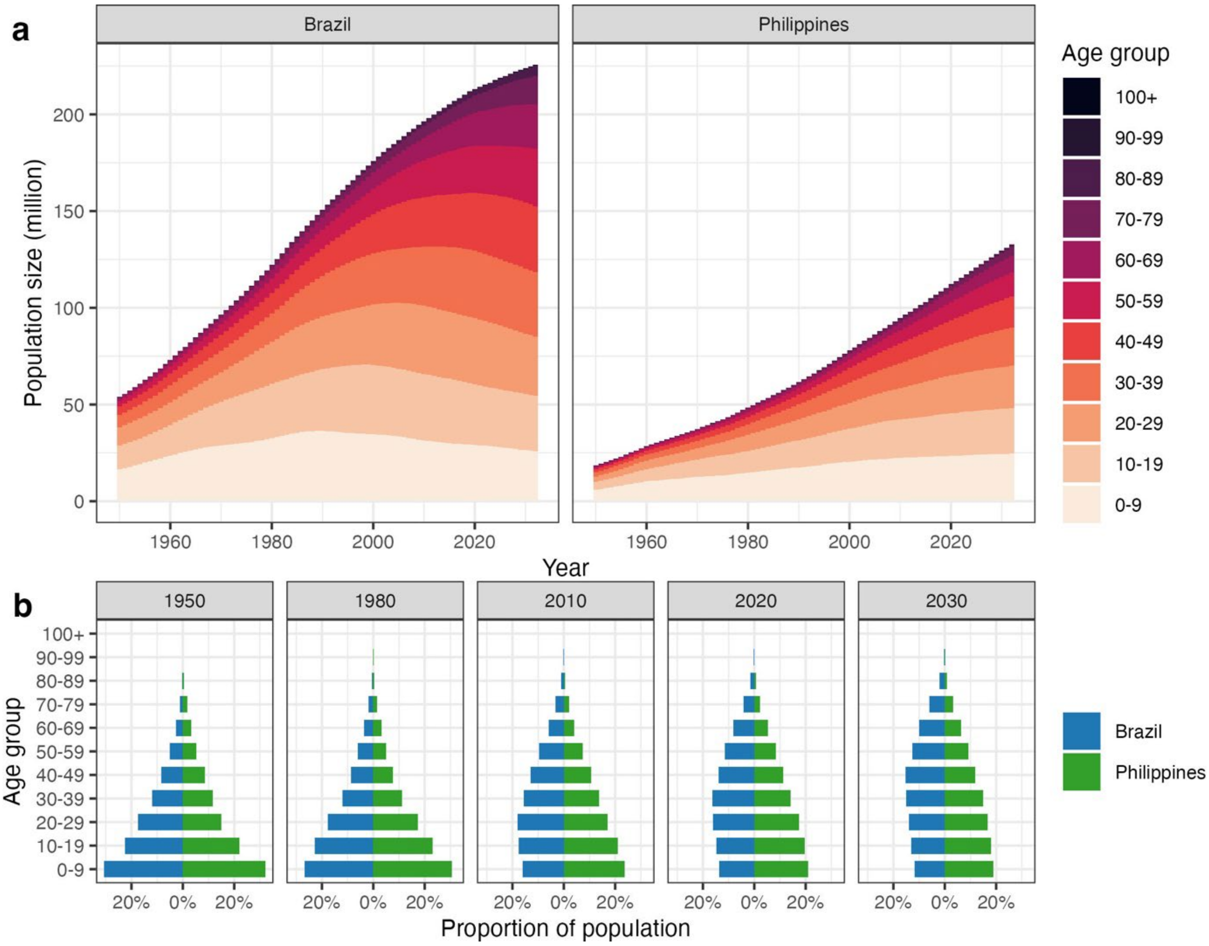

Supplementary Figure 29: Demographies of Brazil and the Philippines used in the transmission model as case studies. a) Population size (y-axis) by age group (colours) from 1950 to 2033 (x-axis) in Brazil and the Philippines (columns). **b)** Age-distribution of by year (columns) for Brazil and the Philippines (colours). Vaccination is assumed to occur from 2024-2033. Demographic estimates are taken from the UN World Population Prospects 2022[22].

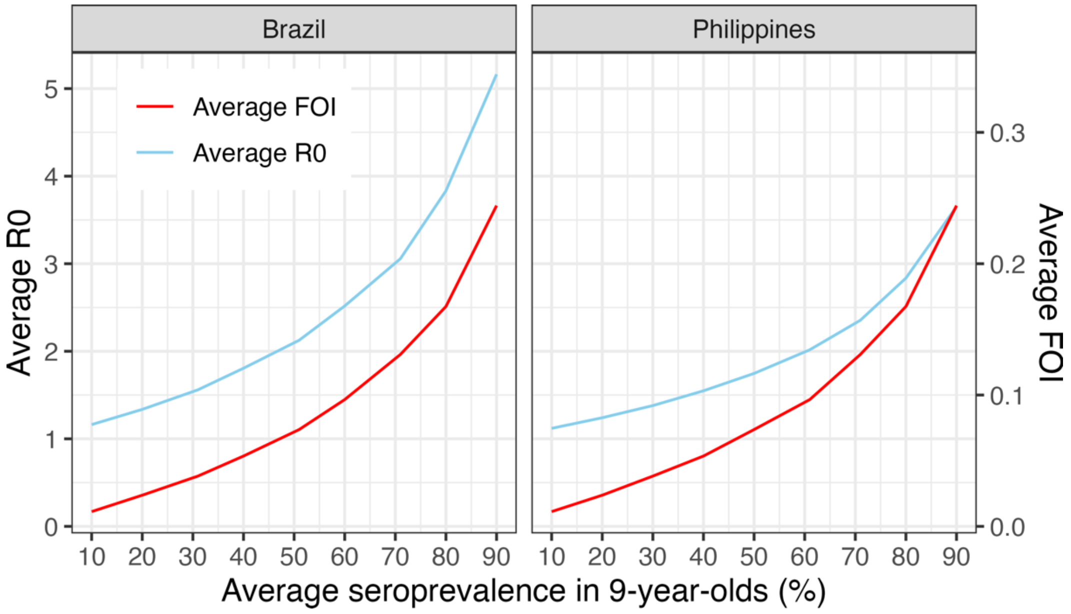

Supplementary Figure 30: Relationship between the mean seroprevalence in 9-year-olds, mean reproduction number (R_0) and the mean total force of infection (FOI) in the transmission model. Model output covering 2020-24 was used for this calibration. The R_0 (blue, y-axis) and the FOI (red, second y-axis) are assigned to match the required equilibrium pre-vaccination mean seroprevalence in 9-year-olds (x-axis), given the demographics of Brazil (left panel) or the Philippines (right panel).

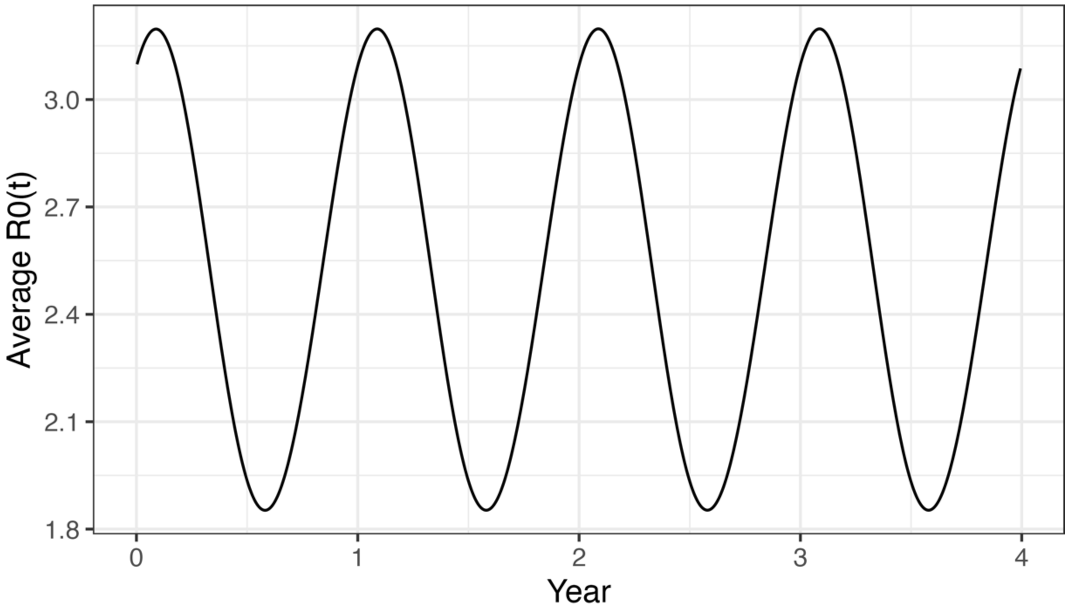

Supplementary Figure 31: Example of the transmission model reproduction number over time R_0(t). Plotted R_0(t) assumes SP9=60% and Brazilian demography.

References

1. Tricou, V. *et al.* Long-term efficacy and safety of a tetravalent dengue vaccine (TAK-003): 4·5-year results from a phase 3, randomised, double-blind, placebo-controlled trial. *The Lancet Global Health* **12**, e257–e270 (2024).
2. Wu, Q. *et al.* Kinetics of IgG antibodies in previous cases of dengue fever-A longitudinal serological survey. *Int J Environ Res Public Health* **17**, 6580 (2020).
3. Salje, H. *et al.* Reconstruction of antibody dynamics and infection histories to evaluate dengue risk. *Nature* **557**, 719–723 (2018).
4. Biswal, S. *et al.* Efficacy of a tetravalent dengue vaccine in healthy children and adolescents. *New England Journal of Medicine* **381**, 2009–2019 (2019).
5. Biswal, S. *et al.* Efficacy of a tetravalent dengue vaccine in healthy children aged 4–16 years: a randomised, placebo-controlled, phase 3 trial. *The Lancet* **395**, 1423–1433 (2020).
6. Rivera, L. *et al.* Three-year efficacy and safety of Takeda's dengue vaccine candidate (TAK-003). *Clinical Infectious Diseases* **75**, 107–117 (2022).
7. López-Medina, E. *et al.* Efficacy of a dengue vaccine candidate (TAK-003) in healthy children and adolescents 2 years after vaccination. *The Journal of Infectious Diseases* **225**, 1521–1532 (2022).
8. Ferguson, N. M. *et al.* Benefits and risks of the Sanofi-Pasteur dengue vaccine: Modeling optimal deployment. *Science* **353**, 1033–1036 (2016).
9. Fonseca-Portilla, R., Martínez-Gil, M. & Morgenstern-Kaplan, D. Risk factors for hospitalization and mortality due to dengue fever in a Mexican population: a retrospective cohort study. *Int J Infect Dis* **110**, 332–336 (2021).
10. Medina, F. A. *et al.* Comparison of the sensitivity and specificity of commercial anti-dengue virus IgG tests to identify persons eligible for dengue vaccination. https://www.medrxiv.org/content/10.1101/2024.04.19.24306097v1 (2024).
11. Sheppard, P. M., Macdonald, W. W., Tonn, R. J. & Grab, B. The dynamics of an adult population of Aedes aegypti in relation to dengue haemorrhagic fever in Bangkok. *Journal of Animal Ecology* **38**, 661–702 (1969).
12. Otero, M., Solari, H. G. & Schweigmann, N. A stochastic population dynamics model for Aedes aegypti: formulation and application to a city with temperate climate. *Bull Math Biol* **68**, 1945–1974 (2006).
13. Focks, D., Brenner, R., Hayes, J. & Daniels, E. Transmission threshold for dengue in terms of Aedes aegypti pupae per person with discussion of their utility in source reduction efforts. *The American journal of tropical medicine and hygiene* **62**, 11–8 (2000).
14. Turelli, M. Cytoplasmic incompatibility in populations with overlapping generations. *Evolution* **64**, 232–241 (2010).
15. WHO. Dengue and severe dengue. https://www.who.int/news-room/fact-sheets/detail/dengue-and-severe-dengue (2024).
16. Gubler, D. J., Suharyono, W., Tan, R., Abidin, M. & Sie, A. Viraemia in patients with naturally acquired dengue infection. *Bull World Health Organ* **59**, 623–630 (1981).
17. Murgue, B., Roche, C., Chungue, E. & Deparis, X. Prospective study of the duration and magnitude of viraemia in children hospitalised during the 1996-1997 dengue-2 outbreak in French Polynesia. *J Med Virol* **60**, 432–438 (2000).
18. Duong, V. *et al.* Asymptomatic humans transmit dengue virus to mosquitoes. *Proc Natl Acad Sci U S A* **112**, 14688–14693 (2015).

19. Christiansen-Jucht, C. D., Parham, P. E., Saddler, A., Koella, J. C. & Basáñez, M.-G. Larval and adult environmental temperatures influence the adult reproductive traits of Anopheles gambiae s.s. *Parasit Vectors* **8**, 456 (2015).
20. Sabin, A. B. Research on dengue during World War II. *The American Journal of Tropical Medicine and Hygiene* **1**, 30–50 (1952).
21. Reich, N. G. *et al.* Interactions between serotypes of dengue highlight epidemiological impact of cross-immunity. *Journal of the Royal Society Interface* **10**, (2013).
22. United Nation.,. World Population Prospects. https://population.un.org/wpp/ (2022).
