# Supplementary material for: Efficacy, public health impact and optimal use of the Takeda dengue vaccine
Source: Nat Med. 2025 Jun 25;31(8):2663–72. doi: 10.1038/s41591-025-03771-y (PMC12353809; doi:10.1038/s41591-025-03771-y)
Supplement: Supplementary file 2 — Reporting Summary [file 41591_2025_3771_MOESM2_ESM.pdf]

nature portfolio

Corresponding author(s): Ilaria Dorigatti

Last updated by author(s): 2025/03/31

Reporting Summary

Nature Portfolio wishes to improve the reproducibility of the work that we publish. This form provides structure for consistency and transparency in reporting. For further information on Nature Portfolio policies, see our Editorial Policies and the Editorial Policy Checklist.

Statistics

For all statistical analyses, confirm that the following items are present in the figure legend, table legend, main text, or Methods section.

n/a	Confirmed	
☐	☒	The exact sample size (*n*) for each experimental group/condition, given as a discrete number and unit of measurement
☐	☒	A statement on whether measurements were taken from distinct samples or whether the same sample was measured repeatedly
☒	☐	The statistical test(s) used AND whether they are one- or two-sided<br>*Only common tests should be described solely by name; describe more complex techniques in the Methods section.*
☐	☒	A description of all covariates tested
☒	☐	A description of any assumptions or corrections, such as tests of normality and adjustment for multiple comparisons
☐	☒	A full description of the statistical parameters including central tendency (e.g. means) or other basic estimates (e.g. regression coefficient) AND variation (e.g. standard deviation) or associated estimates of uncertainty (e.g. confidence intervals)
☒	☐	For null hypothesis testing, the test statistic (e.g. *F*, *t*, *r*) with confidence intervals, effect sizes, degrees of freedom and *P* value noted<br>*Give P values as exact values whenever suitable.*
☐	☒	For Bayesian analysis, information on the choice of priors and Markov chain Monte Carlo settings
☒	☐	For hierarchical and complex designs, identification of the appropriate level for tests and full reporting of outcomes
☒	☐	Estimates of effect sizes (e.g. Cohen's *d*, Pearson's *r*), indicating how they were calculated

Our web collection on statistics for biologists contains articles on many of the points above.

Software and code

Policy information about availability of computer code

Data collection	Data were collated in Excel (version 16.75) from published clinical trials.
Data analysis	Mathematical modelling was performed using R (version 4.4.2), RStudio (version 2023.06.1+524) and cmdstanr (version 0.8.1). Code is available at https://github.com/mrc-ide/qdenga_impact.

For manuscripts utilizing custom algorithms or software that are central to the research but not yet described in published literature, software must be made available to editors and reviewers. We strongly encourage code deposition in a community repository (e.g. GitHub). See the Nature Portfolio guidelines for submitting code & software for further information.

Data

Policy information about availability of data

All manuscripts must include a data availability statement. This statement should provide the following information, where applicable:

- Accession codes, unique identifiers, or web links for publicly available datasets
- A description of any restrictions on data availability
- For clinical datasets or third party data, please ensure that the statement adheres to our policy

Data used for model calibration is available in full at https://github.com/mrc-ide/qdenga_impact.

Research involving human participants, their data, or biological material

Policy information about studies with human participants or human data. See also policy information about sex, gender (identity/presentation), and sexual orientation and race, ethnicity and racism.

Reporting on sex and gender	We did not collate any information on sex or gender.
Reporting on race, ethnicity, or other socially relevant groupings	We did not collate any information on race, ethnicity or other socially relevant groupings.
Population characteristics	The collated case data was stratified by dengue exposure, infecting serotype, age group (4-5 years, 6-11 years, 12-16 years), and trial arm. The study characteristics of the original phase III clinical trial are available at Biswal, Lancet, 2020 (DOI: https://doi.org/10.1016/S0140-6736(20)30414-1)
Recruitment	See Biswal, Lancet, 2020 (DOI: https://doi.org/10.1016/S0140-6736(20)30414-1)
Ethics oversight	See Biswal, Lancet, 2020 (DOI: https://doi.org/10.1016/S0140-6736(20)30414-1)

Note that full information on the approval of the study protocol must also be provided in the manuscript.

Field-specific reporting

Please select the one below that is the best fit for your research. If you are not sure, read the appropriate sections before making your selection.

☒ Life sciences ☐ Behavioural & social sciences ☐ Ecological, evolutionary & environmental sciences

For a reference copy of the document with all sections, see nature.com/documents/nr-reporting-summary-flat.pdf

Life sciences study design

All studies must disclose on these points even when the disclosure is negative.

Sample size	We did not determine sample sizes for this study, as we used all publicly available clinical incidence data collated from published phase III clinical results of the Qdenga vaccine.
Data exclusions	No data were excluded during data collation.
Replication	The data used in this study come from published clinical trail data, which are not available in replicates.
Randomization	Children who met the entry criteria for the phase III clinical trial were randomly assigned 2:1 to receive two doses of TAK-003 or two doses of placebo, 3 months apart. Randomization was stratified according to region (Asia Pacific region or Latin America) and age (4 to 5 years, 6 to 11 years, or 12 to 16 years).All details are available at Biswal, Lancet, 2020 (DOI: https://doi.org/10.1016/S0140-6736(20)30414-1).
Blinding	Details on blinding are provided in Biswal, Lancet, 2020 (DOI: https://doi.org/10.1016/S0140-6736(20)30414-1). This paper describes that "Investigators, participants, and their parents or guardians, and sponsor representatives advising on trial conduct were unaware of trial group assignments. One or more designated pharmacists or vaccine administrators were unmasked at each site, but had no role in the collection or assessment of participant safety data. These individuals accessed randomisation information through a web portal. To maintain masking, medical writers and some sponsor-affiliated authors had access to group and anonymised individual-level study data. Other authors had access only to the data presented in this report. An independent data monitoring committee with responsibility for safety oversight had access to unmasked data on request."

Reporting for specific materials, systems and methods

We require information from authors about some types of materials, experimental systems and methods used in many studies. Here, indicate whether each material, system or method listed is relevant to your study. If you are not sure if a list item applies to your research, read the appropriate section before selecting a response.

Materials & experimental systems

n/a	Involved in the study
☒	☐ Antibodies
☒	☐ Eukaryotic cell lines
☒	☐ Palaeontology and archaeology
☒	☐ Animals and other organisms
☒	☐ Clinical data
☒	☐ Dual use research of concern
☒	☐ Plants

Methods

n/a	Involved in the study
☒	☐ ChIP-seq
☒	☐ Flow cytometry
☒	☐ MRI-based neuroimaging

Plants

Seed stocks	*Report on the source of all seed stocks or other plant material used. If applicable, state the seed stock centre and catalogue number. If plant specimens were collected from the field, describe the collection location, date and sampling procedures.*
Novel plant genotypes	*Describe the methods by which all novel plant genotypes were produced. This includes those generated by transgenic approaches, gene editing, chemical/radiation-based mutagenesis and hybridization. For transgenic lines, describe the transformation method, the number of independent lines analyzed and the generation upon which experiments were performed. For gene-edited lines, describe the editor used, the endogenous sequence targeted for editing, the targeting guide RNA sequence (if applicable) and how the editor was applied.*
Authentication	*Describe any authentication procedures for each seed stock used or novel genotype generated. Describe any experiments used to assess the effect of a mutation and, where applicable, how potential secondary effects (e.g. second site T-DNA insertions, mosiacism, off-target gene editing) were examined.*
